# Enhancement and contextual modulation of visuospatial processing by thalamocollicular projections from ventral lateral geniculate nucleus

**DOI:** 10.1038/s41467-023-43147-9

**Published:** 2023-11-10

**Authors:** Zhong Li, Bo Peng, Junxiang J. Huang, Yuan Zhang, Michelle B. Seo, Qi Fang, Guang-Wei Zhang, Xiaohui Zhang, Li I. Zhang, Huizhong Whit Tao

**Affiliations:** 1 Center for Neural Circuits and Sensory Processing Disorders, Zilkha Neurogenetic Institute, Keck School of Medicine, University of Southern California, Los Angeles, CA 90033 USA; 2 Graduate Program in Neuroscience, University of Southern California, Los Angeles, CA 90033 USA; 3 Graduate Program in Biological and Biomedical Sciences, Keck School of Medicine, University of Southern California, Los Angeles, CA 90033 USA; 4 State Key Laboratory of Cognitive Neuroscience and Learning, Beijing Normal University, Beijing, China; 5 Department of Physiology and Neuroscience, Keck School of Medicine, University of Southern California, Los Angeles, CA 90033 USA

**Keywords:** Thalamus, Sensory processing, Object vision

## Abstract

In the mammalian visual system, the ventral lateral geniculate nucleus (vLGN) of the thalamus receives salient visual input from the retina and sends prominent GABAergic axons to the superior colliculus (SC). However, whether and how vLGN contributes to fundamental visual information processing remains largely unclear. Here, we report in mice that vLGN facilitates visually-guided approaching behavior mediated by the lateral SC and enhances the sensitivity of visual object detection. This can be attributed to the extremely broad spatial integration of vLGN neurons, as reflected in their much lower preferred spatial frequencies and broader spatial receptive fields than SC neurons. Through GABAergic thalamocollicular projections, vLGN specifically exerts prominent surround suppression of visuospatial processing in SC, leading to a fine tuning of SC preferences to higher spatial frequencies and smaller objects in a context-dependent manner. Thus, as an essential component of the central visual processing pathway, vLGN serves to refine and contextually modulate visuospatial processing in SC-mediated visuomotor behaviors via visually-driven long-range feedforward inhibition.

## Abstract (web summary)

Here the authors show that inhibitory projections from the ventral geniculate nucleus to superior colliculus facilitate visual detection of small objects in a visually guided approaching behaviour by providing broad surround suppression to SC neurons.

## Introduction

In the mammalian brain, visual information is propagated throughout subcortical and cortical visual pathways^1,2^. These interconnected visual systems enable the processing of all kinds of visual information encountered by the animal in a natural environment. While the greatest attention has been given to the cortical system, the visual information processing along subcortical visual pathways has been less extensively studied. The dorsal division of the lateral geniculate nucleus (dLGN) of the thalamus is the critical gateway for relaying visual information from the retina into the cortex to generate visual perception^3,4^. One of its adjacent structures, the ventral division of the LGN (vLGN) also receives considerable light-derived signals from the retina^5,6^. However, compared to the dLGN, the functional role of the vLGN has been understudied and poorly understood. Unlike the dLGN, the vLGN is predominantly populated by GABAergic neurons^7–9^ and does not project to the visual cortex^7^. Instead, it sends long-range inhibitory projections to subcortical visual and visuomotor areas in the thalamus and midbrain including the superior colliculus (SC), midbrain reticular nucleus, periaqueductal gray (PAG) and nucleus reuniens (Re)^7,10–12^. Therefore, it has long been thought to play a role in visuomotor processing^13,14^. However, how it plays a role has remained largely unclear.

Recent studies have begun to elucidate the mystery of the vLGN. As sets of non-image-forming retinal ganglion cells (RGCs) such as classes of melanopsin-expressing intrinsically photosensitive RGCs are found to innervate vLGN^15,16^, it has been suggested that vLGN plays a role primarily in non-image-forming functions. Consistent with this idea, vLGN neurons have been shown to be involved in bright light-dependent anti-depressive and anti-nociceptive effects and promotion of spatial memory via their projections to the lateral habenula, PAG and RE, respectively^11,12,17^. In addition, vLGN may be able to encode internal states and serve as a key regulator for adjusting defensive behaviors according to prior experience and the level of perceived visual threat via its projections to SC and RE^18,19^. While imaging of ensemble calcium signals has suggested that vLGN neuron activity reflects the general illumination level of the environment^18^, electrophysiological recording data indicate that vLGN neurons may be able to encode richer feature-specific information^2,6^, especially when considering that vLGN receives inputs from multiple types of RGCs as well as from the visual cortex and SC^2,10^. Therefore, it remains possible that vLGN can also play a role in image-forming functions and shape visual information processing in its target structures such as SC, which is a crucial center for many visuomotor behaviors^20,21^.

In this study, we demonstrated that vLGN played an important role in low-visual-field-dependent visuomotor behaviors such as approaching to a small moving object. Our behavioral tests indicated that vLGN input could improve the animal’s detection ability in the SC-dependent approaching behavior at least partially by enhancing the sensitivity to smaller objects. By systematically characterizing the visual response properties of vLGN neurons with in vivo electrophysiological recording, we found that both light increment and decrement information was processed in vLGN with a moderate bias toward light increments. The vLGN neurons exhibited very broad visual spatial receptive fields (RFs) and a preference to much lower spatial frequencies as compared to other visual structures such as the dLGN, SC and primary visual cortex (V1). This feature allows vLGN neurons to provide surround suppression and to reduce the optimal size of SC neurons, an effect that could be simulated by a “center-surround” model. Furthermore, we demonstrated that the vLGN modulation of SC processing was dependent on visual contexts. Thus, the long-range inhibitory thalamocollicular projection via vLGN may powerfully shape visuospatial processing and facilitate detection of small targets in SC-mediated visuomotor behaviors.

## Results

### The vLGN plays an important role in visually guided approaching behavior

**Fig. 1:**
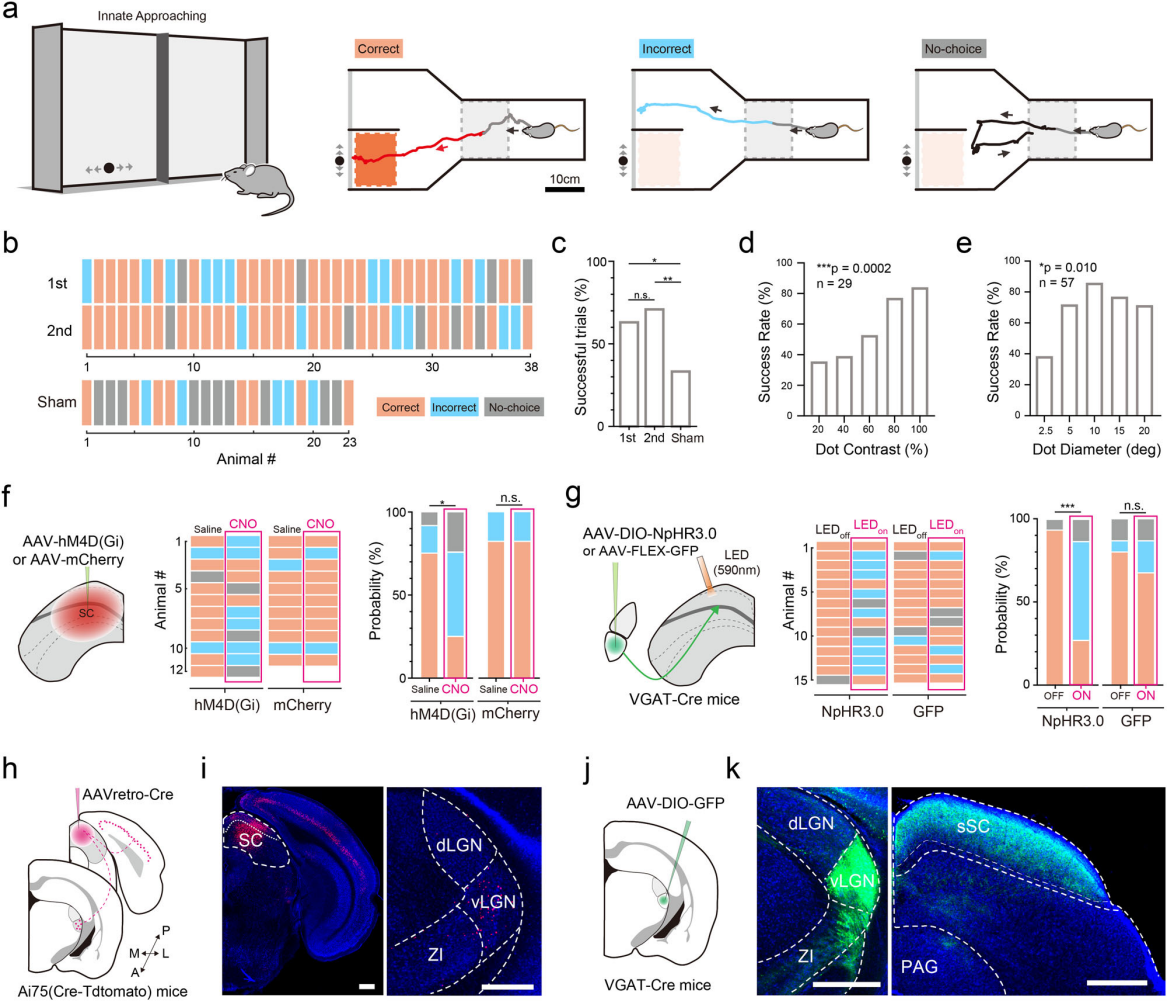
The role of vLGN in SC-dependent innate approaching behavior. **a** Left, schematic of behavioral test. A moving dot was presented on one of two screens. Right, movement tracks of a naïve animal in a correct (red), incorrect (blue) and no-choice (black) trial. Gray, tracks before the onset of dot stimulation. Gray dash box, initiation zone. **b** Top, choices of 38 naïve animals in the first two trials (≥5 min apart). Bottom, choices of 23 naïve animals in sham (no dot) trials. **c** Percentage of success trials. First trial vs. second trial, *p* = 0.6258; first vs. sham, *p = 0.0362; second vs. sham, **p = 0.0045, two-tailed Fisher exact test. **d** Impact of stimulus contrast (at 5° dot size) on the success rate. *n* = 29 mice, ***p = 0.0002, Chi square test. **e** Impact of dot size (at 100% contrast) on the success rate. *n* = 57 mice in total, *p = 0.010, two-tailed chi square test. **f** Left, schematic of chemogenetic silencing of SC. Middle and right, approaching performance for saline and CNO injections in hM4D (*n* = 12) and mCherry (*n* = 11) expressing mice. *p = 0.0391, “n.s.”, not significant, two-tailed Fisher exact test. **g** Left, schematic of optogenetic silencing of vLGN VGAT+ axon terminals in SC. Middle and right, performance for LED-Off and LED-On conditions in NpHR3.0 (*n* = 15) and GFP (*n* = 15) expressing mice. ***p = 0.0005, “n.s.”, not significant, *p* > 0.05, two-tailed Fisher exact test. **h** Schematic of viral injection for retrograde tracing of SC-targeting neurons. **i** Left, fluorescence expression at the injection site. Right, retrogradely labeled neurons in vLGN. Scale bar: 500 μm. ZI zona incerta, dLGN dorsal lateral geniculate nucleus. *n* = 3 animals. **j** Schematic of viral injection for anterograde tracing of vLGN VGAT+ axons. **k** Left, fluorescence expression at the injection site. Note that the fluorescence signal in ZI was largely attributed to axons from vLGN. Right, GFP-labeled axons in SC. Scale bar, 500 μm. PAG periaqueductal gray. *n* = 5 animals. Source data are provided as a Source Data file.

We first investigated whether vLGN could play a role in SC-dependent visuomotor functions. Previous studies have shown that vLGN input to SC could suppress defensive reactions induced by looming stimuli presented in the high visual field^18,19^. Here, we focused on low-visual-field relevant visuomotor behaviors such as approaching in prey capture^22–24^. We designed a behavioral paradigm in which the mouse could choose between one of the two end zones in a Y-shaped maze for approaching (Fig. 1a). A black dot moving back and forth horizontally in the low visual field was presented on the monitor in one randomly chosen end zone, at a time when the mouse entered the initiation zone and was facing the monitors (see “Methods”). Such moving dot was attractive to a naïve mouse so that it guided the animal to enter the end zone and approach the screen where the dot was presented with a high success rate, while in the sham condition the animal mostly did not choose either end zone or randomly entered either end zone (Fig. 1b, c). Either reducing the contrast or changing the size of the dot could reduce the success rate (Fig. 1d, e), indicating that this innate behavior is visually dependent. That is, it depends on the visibility and visual feature of the stimulus.

We then tested whether SC and vLGN were involved in this visually guided approaching behavior. First, we chemogenetically disrupted SC activity by injecting adeno-associated virus (AAV) expressing an inhibitory DREADD receptor (AAV-hSyn-hMD4(Gi)) (Supplementary Fig. 1a). The virus would infect both excitatory and inhibitory neurons in SC, with the latter recently found to be more dominant in particular in superficial layers^25,26^. We found that the disruption of SC activity with CNO administration impaired the performance of animals by increasing the percentage of both incorrect and no-choice trials, whereas CNO had no effect in mCherry control animals (Fig. 1f). This result supports the notion that the visuomotor behavior is SC-dependent^20,22,27^.

To silence the vLGN, we injected AAV encoding Cre-dependent halorhodopsin (NpHR3.0) in VGAT-Cre mice (Supplementary Fig. 1b, c), as previous studies have shown that vLGN is predominantly populated by GABAergic neurons^7–9^ which on the other hand are very sparse in dLGN^28^. Similar to the SC silencing, the optical inhibition of vLGN VGAT+ neurons by amber light (590 nm) throughout the test trial greatly reduced the success rate, while the same optical stimulation had no effect in GFP control animals (Supplementary Fig. 1d). By placing the optic fiber above SC, we optically silenced the vLGN→SC axon terminals and found that this also reduced the success rate (Fig. 1g). These results demonstrate that the vLGN plays an important role in the SC-dependent visually guided approaching behavior through at least partially its projection to SC. The negative effect of silencing vLGN or the vLGN→SC projection on the performance was unlikely attributed to negative emotion-related aversion, as a two-chamber real-time place preference (RTPP) test showed that neither the silencing nor the activation significantly changed the time spent in the chamber associated with photo-stimulation or the locomotion speed in that chamber as compared to control conditions (Supplementary Fig. 2a–f). The optogenetic manipulations did not affect the speed of approaching either (Supplementary Fig. 2g–i). These data suggest that activity of vLGN does not increase arousal or motivation for exploration, unlike its neighboring structure, the zonal incerta (ZI)^29^. Together, our results suggest that vLGN activity likely influences the approaching behavior by affecting visual processing in SC.

We thus examined the anatomical connection between vLGN and SC. Injection of a retrograde tracer, AAVretro-Cre, into SC of Ai75 (Cre-dependent nucleus-localized tdTomato) mice (Fig. 1h) revealed abundant SC-projecting neurons in vLGN, but not in its neighboring region dLGN (Fig. 1i). Anterograde tracing of vLGN axons by injecting AAV encoding Cre-dependent GFP in VGAT-Cre mice (Fig. 1j) revealed that they were projected to the SC and ZI (Fig. 1k), in line with previous reports^10,30–32^. In particular, the superficial layers of SC (sSC, the visual SC) appeared to be a major targeting area of vLGN axons, while the axons in the intermediate and deep layers of SC (i.e., the motor SC) were sparser (Fig. 1k). This result appears different from previous studies showing strong vLGN projections to the motor SC and dorsal PAG^18,19^, possibly due to different experimental parameters. Our anatomical result raises the possibility that vLGN neurons may be able modulate visually evoked activity in SC serving the low visual field^27,33^.

### The vLGN activity facilitates detection of small visual objects

**Fig. 2:**
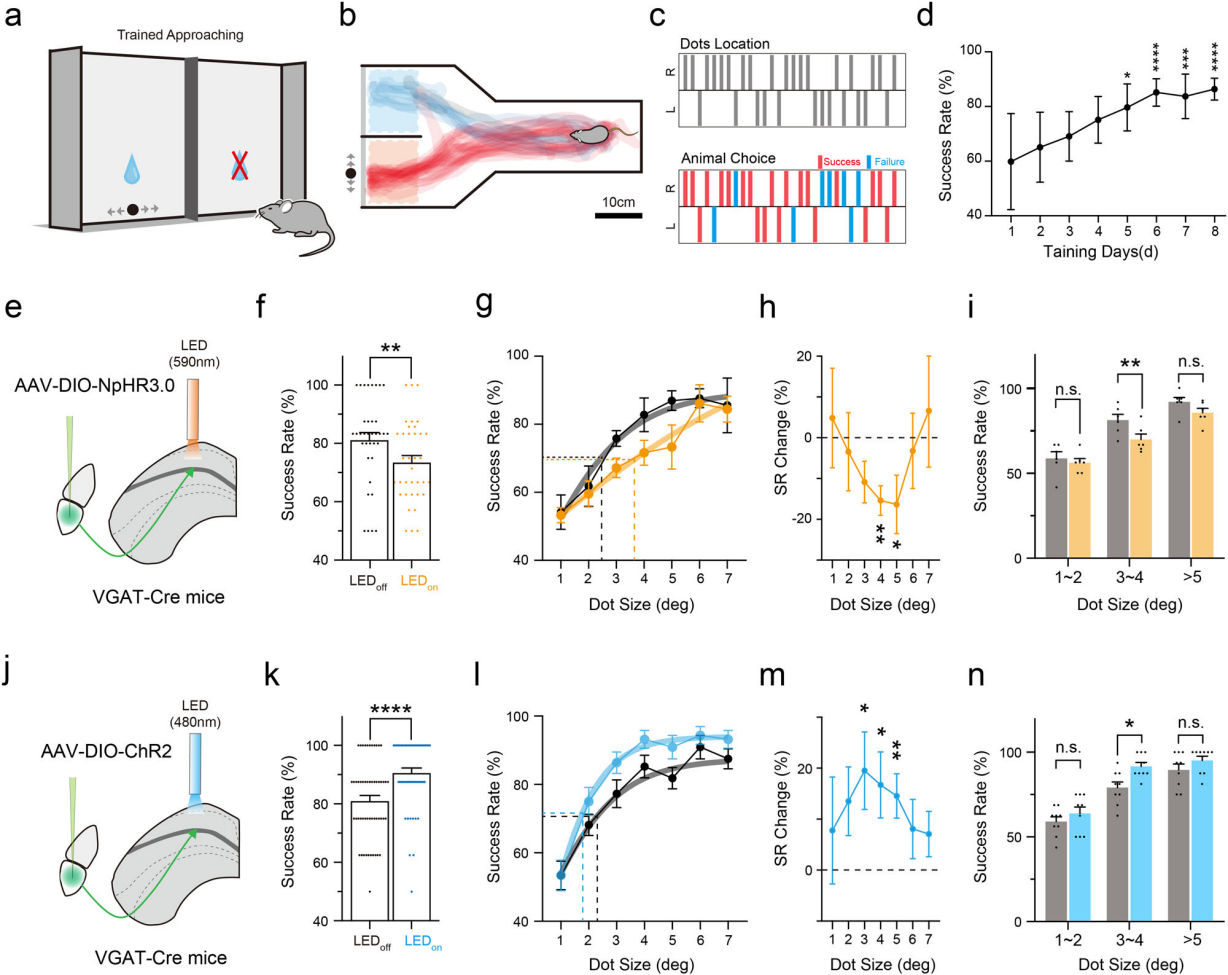
Influence of vLGN on the performance of learned approaching behavior. **a** Schematic of a learned visually guided approaching task. The water-restricted animal was trained to approach the presented moving dot to receive water reward. **b** Superimposed movement tracks of all trials during the 8-day training for an example animal. The side of dot presentation was randomly chosen for each trial but was aligned in this plot for better illustration. **c** Dot locations (upper) and behavioral choices (lower) for an example task session. Red, correct; blue, incorrect. **d** Average success rate over training days (*n* = 11 mice). Compared to the first day, d5, *p = 0.0183; d6, ****p = 0.84 × 10^−4^; d7, ***p = 0.0007; d8, ****p = 0.11 × 10^−4^, Kruskal–Wallis test and Dunn’s multiple-comparisons test. **e** Schematic of viral injection for optogenetic silencing of vLGN VGAT+ axon terminals in SC. **f** Overall success rate (all sessions with >1° dot size were included) without (gray) and with (orange) photo-inhibition. LED_Off_ vs. LED_On_, 81% ± 3% vs. 74% ± 2%, *n* = 36 sessions from 6 mice, **p = 0.0099, two-tailed Wilcoxon matched-pairs signed rank test. **g** Average success rate at different dot sizes without (gray) and with (orange) photo-inhibition. Curves after fitting are shown. Dashed vertical lines indicate dot size for 50% of peak performance. *n* = 6 mice. **h** Relative changes in success rate (%) after photo-inhibition at different dot sizes. **p = 0.002, *p = 0.046, two-tailed paired *t*-test. **i** Average success rates for different dot size ranges in LED-Off (gray) LED-On (orange) conditions. Bar represents s.e.m. **p = 0.0015; n.s., *p* > 0.05, two-tailed paired *t-*test. **j**–**n** Similar to (**e**–**i**) but for optogenetic activation of vLGN VGAT+ axons in SC. Statistics: (**k**) LED_Off_ vs. LED_On_, *n* = 54 sessions from 9 mice, ****p < 0.0001, two-tailed Wilcoxon matched-pairs signed rank test; (**m**) *p = 0.021, *p = 0.019, **p = 0.004, two-tailed paired *t-*test; (**n**) **p = 0.0011, two-tailed paired *t-*test. Data are presented as mean ± s.e.m. in (**d**, **f**–**i** and **k**–**n**). Source data are provided as a Source Data file.

We next wondered how vLGN would influence the approaching behavior at different stimulus features, such as different dot sizes. It is however difficult to test different dot sizes with the innate behavior because the mouse would quickly become adapted to the dot stimulation and neglect it (Supplementary Fig. 1e). To resolve this issue, we redesigned the experiment by training water-deprived mice to approach the moving dot to receive water reward (Fig. 2a–c). In our experiments, it took about 1 week of training for the animal to achieve stable high performance (Fig. 2d). More than 50 trials could be tested each day so that we could measure the approaching performance under visual stimuli of varying sizes. Consistent with the innate behavior, optogenetic suppression of the vLGN→SC axons by expressing halorhodopsin (NpHR3.0) in VGAT-Cre mice overall decreased the success rate (Fig. 2e, f). Furthermore, we observed a shift of the dot size at which performance reached 50% of maximum level (DS50%) toward a larger value (Fig. 2g), suggesting a reduced sensitivity to smaller dot sizes. When comparing relative changes in the success rate across different dot sizes, we found that the strongest effect occurred within a range of 3°‒5° dot sizes (Fig. 2h, i). Conversely, activation of vLGN→SC axons increased the overall success rate (Fig. 2j, k) and shifted the DS50% toward a smaller value (Fig. 2l), suggesting enhanced sensitivity to smaller stimulus sizes. The strongest effect on the success rate also occurred within a range of 3°‒5° dot sizes (Fig. 2m, n). GFP control mice did not show such an effect (Supplementary Fig. 3a–f). The changes in performance could not be explained by changes in motivation or arousal, since no obvious difference in the approaching speed or inter-trial-interval was observed between LED-OFF and LED-ON conditions (Supplementary Fig. 3g, h). Together, our results suggest that vLGN input can facilitate the visually guided approaching behavior by modulating the visual sensitivity to stimuli of specifically small sizes.

### Visual functional properties of vLGN neurons

**Fig. 3:**
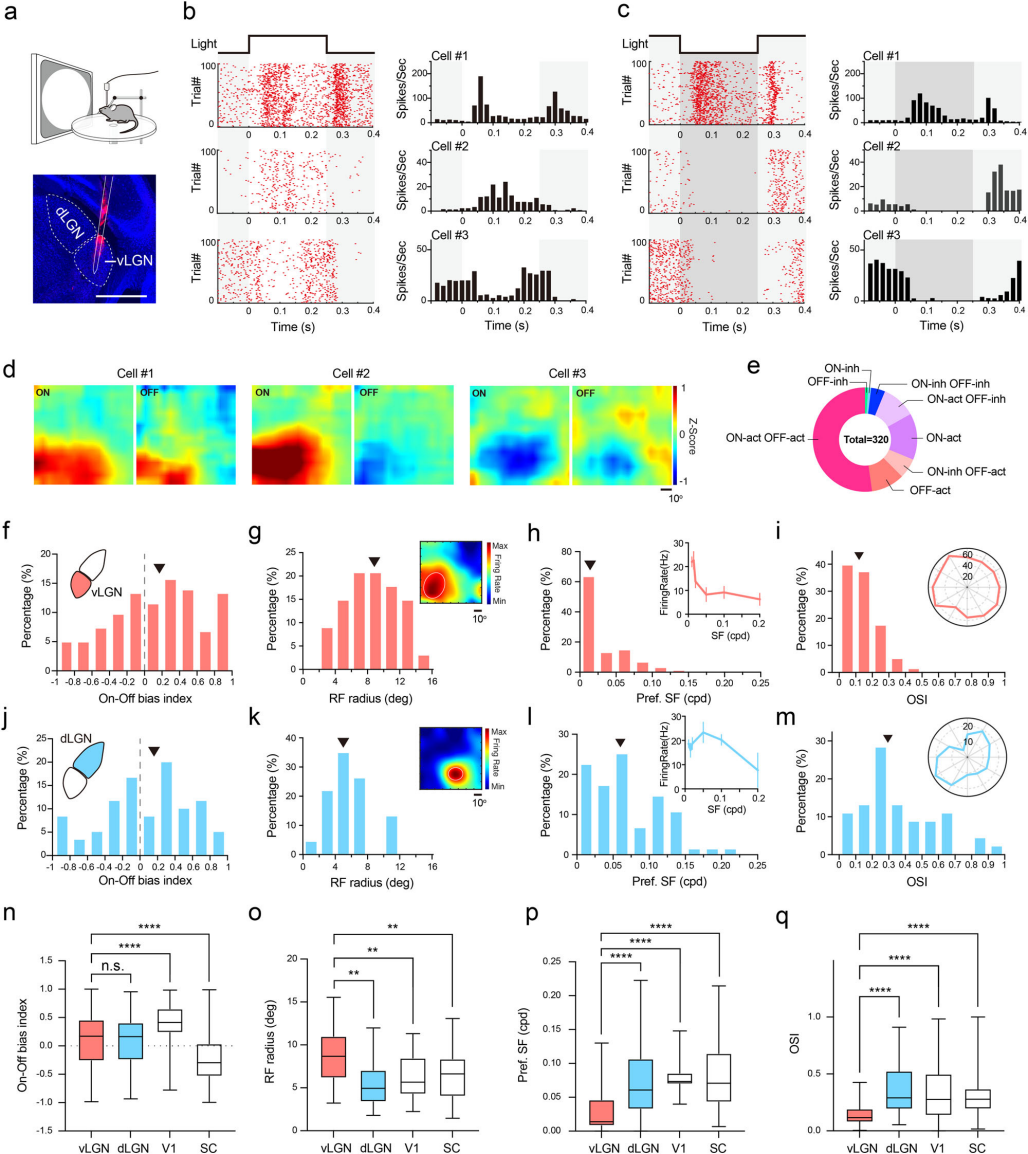
Visual response properties of vLGN neurons. **a** Top, schematic of electrophysiological recording. Bottom, example electrode track marked by DiI. Scale bar, 500 µm. Raster plot (left) and peri-stimulus spike-time histogram (PSTH) (right) for responses of three example cells to a white (**b**) or black (**c**) flashing disk (duration marked by white or gray shade). **d** ON and OFF receptive fields (RFs) for the same cells. Scale bar, 10°. **e** Proportions of functional classes based on ON/OFF response properties. *n* = 320 neurons from 7 mice. **f** Distribution of ON-OFF bias indices. Black arrowhead indicates the median. *n* = 167 neurons. **g** Distribution of RF radiuses (after fitting). Arrowhead marks the median. *n* = 34 neurons. Inset, receptive field of an example vLGN neuron. Only the response to the preferred contrast is shown. Scale bar, 10°. **h** Distribution of preferred spatial frequencies (SFs). *n* = 111 neurons. Inset, SF tuning curve of an example neuron (mean ± s.e.m.). **i** Distribution of orientation selectivity indices (OSIs). *n* = 81 neurons. Inset, polar plot of orientation tuning for an example cell. Axis indicates firing rate (Hz). **j**–**m** Similar to (**f**–**i**) but for dLGN neurons. **n** Summary of On-Off bias index. *n* = 167, 60, 117 and 537 neurons from 7, 3, 2, 10 mice for vLGN, dLGN, V1 and SC, respectively. Left-to-right, n.s., *p* > 0.99, ****p = 7.3 × 10^−7^, 5.0 × 10^−12^, Kruskal–Wallis test and Dunn’s multiple comparison test. **o** Summary of RF radius. *n* = 34, 23, 51 and 96 neurons, respectively. Left-to-right, **p = 0.0017, 0.0016, 0.0037, Kruskal–Wallis test and Dunn’s multiple comparison test. **p** Summary of Pref. SF. *n* = 111, 76, 99 and 204 neurons, respectively. Left-to-right, ****p = 3.7 × 10^−8^, <1.0 × 10^−15^, <1.0 × 10^−15^, Kruskal–Wallis test and Dunn’s multiple comparison test. **q** Summary of OSI. *n* = 81, 46, 193 and 143 neurons, respectively. Left-to-right, ****p = 1.8 × 10^−8^, 1.6 × 10^−10^, 8.6 × 10^−11^, Kruskal–Wallis test and Dunn’s multiple comparison test. **n**–**q** Center lines indicate the median, limits indicate the upper/lower quartiles, whiskers represent the minimum/maximum. Source data are provided as a Source Data file.

To understand how vLGN could play a role in modulating SC processing, we systematically characterized the visual response properties of vLGN neurons themselves. Using multi-channel probe recording in awake head-fixed mice (Supplementary Fig. 4a), we recorded neuronal responses to flash visual stimuli, which were a 90°-diameter white (ON) or black (OFF) disc presented over gray background (Fig. 3a). In our recorded vLGN neuron population, 77% (320 of 416 units) had significant responses to at least one of the two flash stimuli. The great majority (300 of 320) of these responsive neurons showed an excitatory response to at least one of the two stimuli, while a minority (74 of 320) showed a suppressive response to either the ON or OFF stimulus. Three example cells are shown in Fig. 3b, c. The first one exhibited an excitatory response to both ON and OFF stimuli, the second one was activated by ON but suppressed by OFF stimulus, and the third one was suppressed by both ON and OFF stimuli. We also used sparse-noise stimuli and spike triggered averaging to map the spatial ON and OFF receptive fields (RFs) of vLGN neurons (Fig. 3d), which revealed the same response polarities as those revealed by the large discs. Based on the ON/OFF response patterns, we categorized the vLGN neurons into different classes (Fig. 3e). More than half (52%, 167 of 320) of the neurons could be activated by both ON and OFF stimuli (i.e., ON-act OFF-act). Some only responded to one contrast (either ON or OFF) but not to the other (ON-act, *n* = 46; OFF-act, *n* = 33). Some had opposite responses (activation and inhibition) to the opposite contrasts (ON-act OFF-inh, *n* = 34; OFF-act ON-inh, *n* = 20). A few neurons exhibited only a suppressive response to either ON or OFF stimulus (ON-inh, *n* = 3; OFF-inh, *n* = 3; ON-inh OFF-inh, *n* = 14). While the complexity of the visual response properties in vLGN as we observed here is largely in line with previous studies^6,34^, a striking observation here is that most vLGN neurons can be activated by both ON and OFF stimuli, indicating that they can encode both light increments and decrements and thus likely a rich repertoire of visual information.

Nevertheless, there was an overall response bias toward light increments, which was also observed in several previous studies^18,19,35^. More neurons were activated by ON than OFF stimulus (*n* = 247 vs. 220). For the neurons responding to both ON and OFF stimuli, they had a relatively larger response to ON than OFF stimulus, as shown by the skewed distribution of the ON-OFF bias index (Fig. 3f). This ON bias was similar to that in dLGN (Fig. 3j) but was weaker than that in V1 (ON-OFF bias index: vLGN, 0.10 ± 0.04; dLGN, 0.06 ± 0.06; V1, 0.43 ± 0.03; SC, −0.21 ± 0.02; mean ± s.e.m.) (Fig. 3n and Supplementary Fig. 4c). On the contrary, neurons in SC had a strong bias toward light decrements (Fig. 3n and Supplementary Fig. 4f).

There are distinct features observed in the vLGN neuronal responses. Consistent with previous studies^6,35–37^, we found that RFs of vLGN neurons were larger than dLGN neurons (Fig. 3g, k, o) as well as V1 and SC neurons (RF radius: vLGN, 8.7° ± 0.5°; dLGN, 5.7° ± 0.6°; V1, 6.2° ± 0.3°; SC, 6.4° ± 0.3°; mean ± s.e.m.) (Fig. 3o and Supplementary Fig. 4d, g). We also applied drifting gratings of different spatial frequencies (SFs) to measure SF tuning and found that the preferred spatial frequency (Pref. SF) of vLGN neurons was lower than dLGN, V1 and SC neurons (Pref. SF: vLGN, 0.029 ± 0.003 cycle/s; dLGN, 0.067 ± 0.006 cycle/s; V1, 0.081 ± 0.002 cycle/s; SC, 0.075 ± 0.003 cycle/s; mean ± s.e.m.) (Fig. 3h, l, p and Supplementary Fig. 4e, h). Moreover, we examined orientation and direction tuning and found that both orientation and direction selectivity of vLGN neurons was weak. The orientation selectivity index (OSI) of vLGN neurons was lower than dLGN neurons as well as V1 and SC neurons (OSI: vLGN, 0.14 ± 0.01; dLGN, 0.36 ± 0.03; V1, 0.33 ± 0.02; SC, 0.32 ± 0.01; mean ± s.e.m.) (Fig. 3i, m, q). The direction selectivity index (DSI) was similarly low in the two LGN areas (DSI: vLGN, 0.23 ± 0.02; dLGN, 0.27 ± 0.03; mean ± s.e.m.) (Supplementary Fig. 4i).

In a short summary, we found that most vLGN neurons responded with excitatory responses to both light increments and decrements but with a moderate bias toward light increments. They had very large spatial RFs, very low Pref. SFs as well as poor orientation and direction selectivity.

### Depth-dependent inhibitory modulation of SC responses by vLGN input

**Fig. 4:**
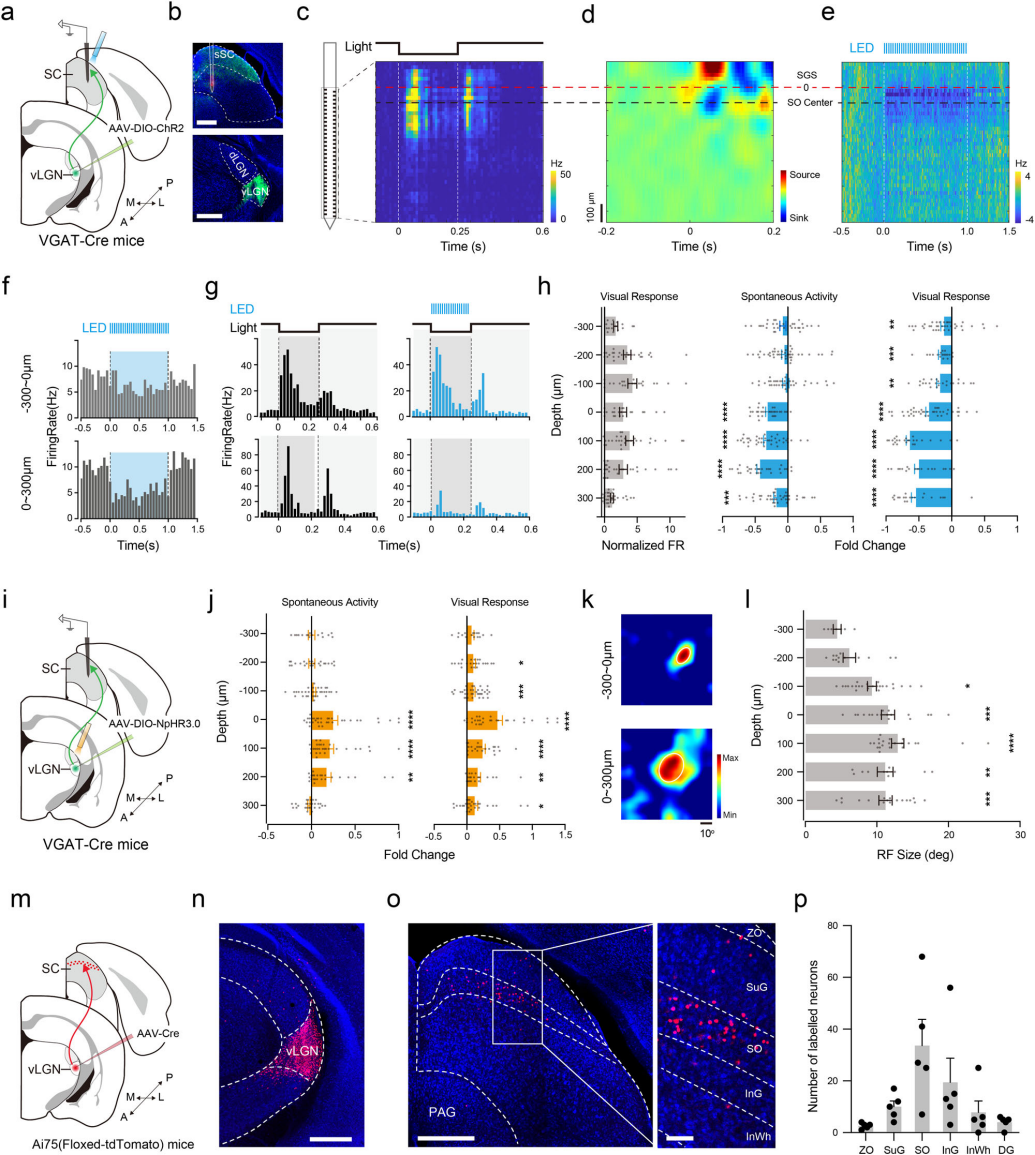
Depth-dependent inhibitory effects of vLGN input to SC. **a** Schematic of experimental setup. **b** Example electrode track (top) and expression of ChR2-EYFP in vLGN (bottom). Scale bar, 500 μm. *n* = 3 replicates. **c** Example heatmap for SC responses to a flashing dark disk (top inset) across different channels. **d** Heatmap for current source density in the same recording. Red dash line marks the boundary between SGS and SO. **e** Spontaneous spikes responding to the optogenetic activation alone. **f** PSTHs for spontaneous spikes responding to the optogenetic activation in two example neurons. **g** PSTHs for spike responses responding to a flash dark disk without (left) and with (right) LED light pulses in the same neurons shown in (**f**). **h** Normalized visually evoked firing rate (FR) (left), and fold changes in spontaneous (middle) and evoked (right) spike rates by activating vLGN axons, at different SC depths. ****p < 0.0001, ***p < 0.001, **p < 0.01, two-tailed one sample *t*-test. Left-to-right, *n* = 176, 252, 182 neurons. **i** Schematic of recording in SC while photo-inhibiting vLGN neurons. **j** Fold changes in spontaneous (left) and evoked (right) spike rates at different depths. ****p < 0.0001, ***p < 0.001, **p < 0.01, *p < 0.05, two-tailed one sample *t*-test. Left-to-right, *n* = 185, 177 neurons. **k** RFs of two example neurons at different SC depths. **l** Average RF radius at different SC depths. ****p < 0.0001, ***p < 0.001, **p < 0.01, *p < 0.05, Kruskal–Wallis test and Dunn’s multiple comparison test. *n* = 112 neurons. **m** Schematic anterograde transsynaptic labeling of vLGN-recipient SC neurons. **n** TdTomato expression at the injection site. Scale bar, 500 μm. **o** Labeled vLGN-recipient neurons in SC. Scale bar, 500 μm. Right, enlarged boxed area. Scale bar, 100 μm. ZO zonal, SuG superficial gray, SO stratum opticum, InG intermediate gray, InWh intermediate white, DG deep gray. **p** Numbers of anterogradely labeled neurons in different SC sublayers. *n* = 5 sections from 3 mice. Data are presented as mean ± s.e.m. in (**h**, **j**, **l** and **p**). Source data are provided as a Source Data file.

The anatomical data (Fig. 1k) suggest that vLGN input might modulate SC visual activity. To understand the degree of this modulation, we performed electrophysiological recording in both the medial and lateral parts of SC using a 64-channel silicon probe (Supplementary Fig. 4b) while optogenetically activating ChR2-expressing vLGN VGAT+ axons in SC (Fig. 4a, b). Consistent with previous reports^38–40^, robust responses to a flash dark stimulus were evoked across different depths mainly in the dorsal half of SC (Fig. 4c). Based on the current source density (CSD) analysis of local field potentials (LFPs), we identified the upper boundary of the stratum opticum (SO) (Fig. 4d, set as “0”), to which recording depths were aligned^40,41^ (see “Methods”). The optogenetic activation of vLGN VGAT+ axons suppressed the spontaneous firing rate (Fig. 4e, f) and evoked firing rate (Fig. 4g) of SC neurons in a depth-dependent manner. The strongest effect was observed within SO and immediately below it (Fig. 4h), corresponding to the lower portion of sSC and upper portion of the intermediate gray layer of SC (InG), reminiscent of previous slice recording results^19^. Such inhibitory effect was present in both the lateral and medial parts of SC (fold change: lateral, −0.48 ± 0.03, *p* < 0.0001, *n* = 104; medial, −0.31 ± 0.03, *p* < 0.0001, *n* = 40, one sample *t*-test). Conversely, optogenetic inhibition of vLGN VGAT+ neurons expressing NpHR3.0 (Fig. 4i) increased spontaneous and evoked firing rates of SC neurons (Fig. 4j). Again, the strongest effect was observed within SO and immediately below it. We then focused on these depths in later experiments to examine how vLGN input modulates visually evoked activity of SC neurons. SC neurons at these depths exhibited on average a larger visual RF than those more superficial (Fig. 4k, l), reminiscent of a previous in vivo electrophysiological study^40^. Using an anterograde transsynaptic labeling approach^42,43^, we examined the distribution of SC neurons receiving direct vLGN input by injecting AAV1-Cre into vLGN of Ai75 mice (Fig. 4m, n). Consistent with the electrophysiological data, most of the vLGN-recipient SC neurons were located in SO and to a lesser degree in InG (Fig. 4o, p).

### The vLGN input shapes visuospatial processing in SC

**Fig. 5:**
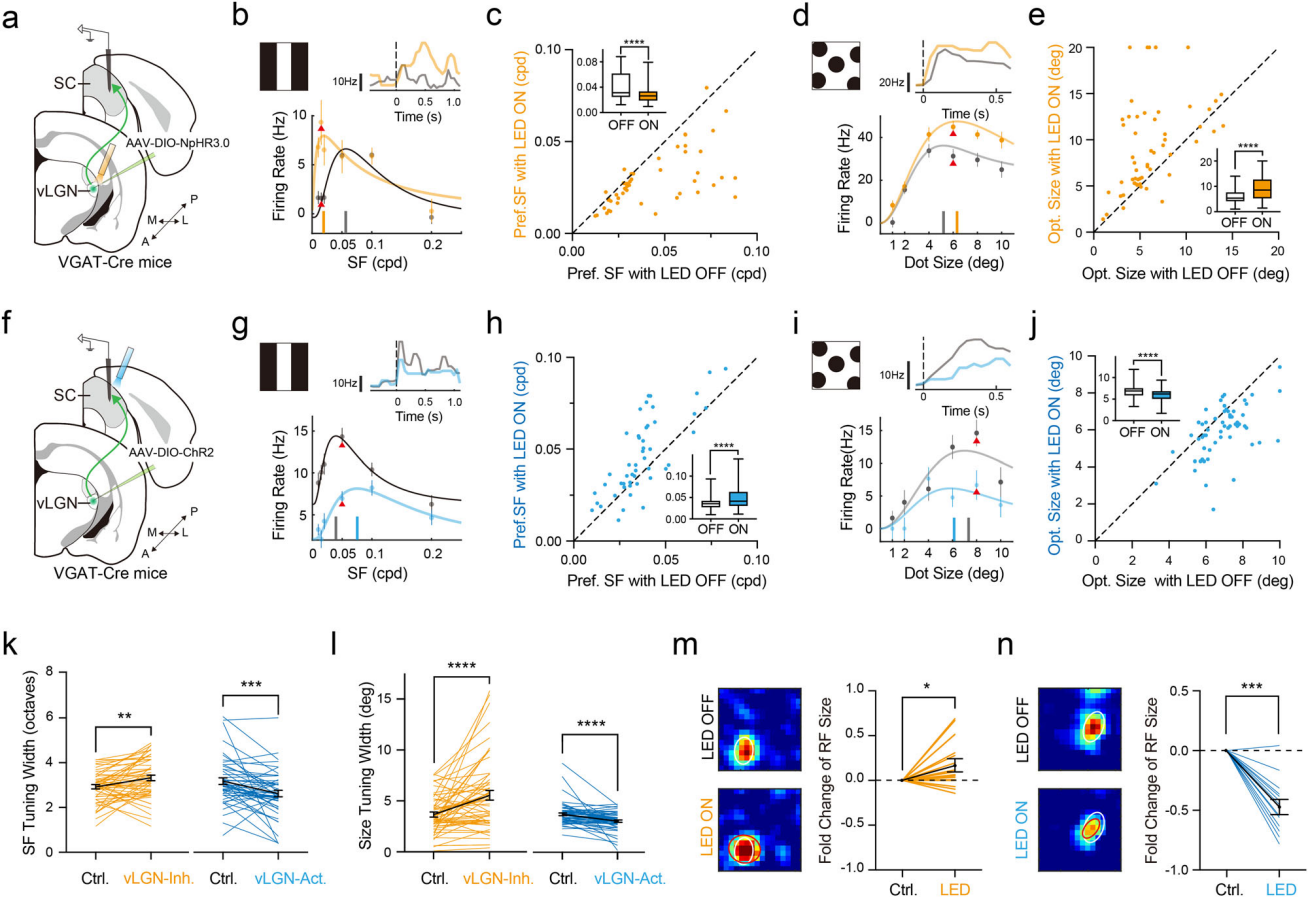
Modulation of spatial processing of SC neurons by vLGN input. **a** Experimental setup. **b** Spatial frequency tuning of an example SC neuron without (gray) and with (orange) photo-inhibition of vLGN VGAT+ neurons. Vertical bar marks the preferred SF. *n* = 10 trials. Inset, PSTHs of spike responses at the SF marked by red arrowhead. **c** Preferred SF with vs. without photo-inhibition. Dashed line is the unity line. Inset, Pref. SFs in LED-OFF and LED-ON conditions. Center line indicates median, limit indicates upper/lower quartile and whisker indicates minimum/maximum for all box plots. *n* = 51 neurons, ****p = 1.4 × 10^−6^, two-tailed paired *t*-test. **d** Size tuning of an example SC neuron. *n* = 20 trials. Vertical bar marks the optimal size. **e** Optimal size with vs. without optogenetic inhibition. Inset, optimal sizes in LED-OFF and LED-ON conditions. *n* = 58 neurons, ****p = 2.3 × 10^−6^, two-tailed paired *t*-test. **f**–**j** Similar to (**a**–**e**) but for optogenetic activation (blue) of vLGN VGAT+ axons in SC. **g** *n* = 10 trials; **h** ****p = 1.6 × 10^−5^, two-tailed paired *t*-test, *n* = 51 neurons; **i** *n* = 20 trials; **j** ****p = 3.7 × 10^−7^, two-tailed paired *t*-test. *n* = 65 neurons. **k** SF tuning bandwidth in LED-OFF vs. LED-ON condition. **p = 0.002, ***p = 0.0009, two-tailed paired *t*-test. *n* = 51 (orange) and 51 (blue) neurons. **l** Size tuning bandwidth in LED-OFF vs. LED-ON condition. ****p = 6.2 × 10^−5^ (orange), 4.2 × 10^−5^ (blue), two-tailed paired *t*-test. *n* = 58 (orange) and 65 (blue) neurons. **m** Left, RFs of an example SC neuron in LED-OFF (top) and LED-ON (bottom) conditions. White and red ovals represent RF fittings for LED-OFF and LED-On conditions, respectively. Right, fold change in RF size after suppressing vLGN. *p = 0.011, two-tailed Wilcoxon matched-pairs signed rank test. *n* = 18 neurons. **n** Similar to (**m**) but for activation of vLGN axons in SC. ***p = 0.0005, two-tailed Wilcoxon matched-pairs signed rank test. *n* = 13 neurons. Data are presented as mean ± s.d. in (**b**, **d**, **g**, **i**); as mean ± s.e.m. in (**k**–**n**). Source data are provided as a Source Data file.

As the SF preference of vLGN is distinct from SC (Fig. 3p), we wondered whether vLGN could play a role in shaping the SF tuning of SC neurons. To address this question, we recorded responses of SC neurons to drifting gratings of different SFs without and with optogenetically manipulating vLGN input in awake head-fixed mice (Fig. 5a, f). In these experiments, we made recordings mostly from the lateral part of SC (Supplementary Fig. 4b). Our data showed that photo-inhibition of vLGN VGAT+ neurons increased firing rate responses mostly to low-SF stimuli (Supplementary Fig. 5a) and shifted the Pref. SF of SC neurons toward a smaller value (Fig. 5b, c), meaning that without the vLGN input the SC neurons prefer wider gratings. On the contrary, photo-activation of vLGN axons in SC decreased firing rate responses mostly to low-SF stimuli (Supplementary Fig. 5b) and shifted the Pref. SF of SC neurons toward a larger value (Fig. 5g, h). The manipulations also affected the sharpness of SF tuning, with the suppression of vLGN neurons broadening the tuning bandwidth while activating their axons in SC reducing the tuning bandwidth (Fig. 5k).

Considering a broader spatial integration in vLGN than SC neurons as indicated by their different RF sizes and SF preferences, we wondered whether vLGN could affect the preferred stimulus size of SC neurons. To test this idea, we measured the size tuning of vLGN and SC neurons by presenting a set of moving discs (in the same direction and speed) of various diameters. The optimal size is defined as the size of the discs to which the neuronal response reaches the maximum value. Consistent with their larger RFs and lower Pref. SFs, the optimal size of vLGN neurons was significantly larger than SC neurons (Supplementary Fig. 6). The optimal size of SC neurons was enlarged when vLGN VGAT+ neurons were inhibited (Fig. 5d, e and Supplementary Fig. 5c) while reduced when their axons in SC were activated (Fig. 5i, j and Supplementary Fig. 5d). The sharpness of the size tuning was also affected: inhibiting the vLGN neurons broadened the tuning while activating their axons in SC sharpened the tuning (Fig. 5l). Consistent with the effects on size preferences, inhibiting the vLGN neurons broadened the spatial RF of SC neurons, increasing the RF size by 18.0% ± 6.1% (Fig. 5m), whereas activating their axons in SC sharpened the SC neuron’s RF, decreasing the RF size by 46.0% ± 6.4% (Fig. 5n). In addition, we found that orientation tuning was also affected by the activity manipulations. Inhibiting the vLGN neurons weakened while activating their axons in SC enhanced orientation selectivity of SC neurons, without significantly affecting the preferred orientation (Supplementary Fig. 7a–f). Furthermore, inhibiting vLGN VGAT+ axons in SC recapitulated the effects on SF tuning, size tuning and orientation tuning observed in experiments of inhibiting vLGN VGAT+ neurons (Supplementary Fig. 7g–o). Altogether, our data suggest that vLGN can profoundly shape the spatial processing properties of SC neurons via its GABAergic projection to SC.

### The vLGN input modulates the SC optimal size through a surround suppression mechanism

**Fig. 6:**
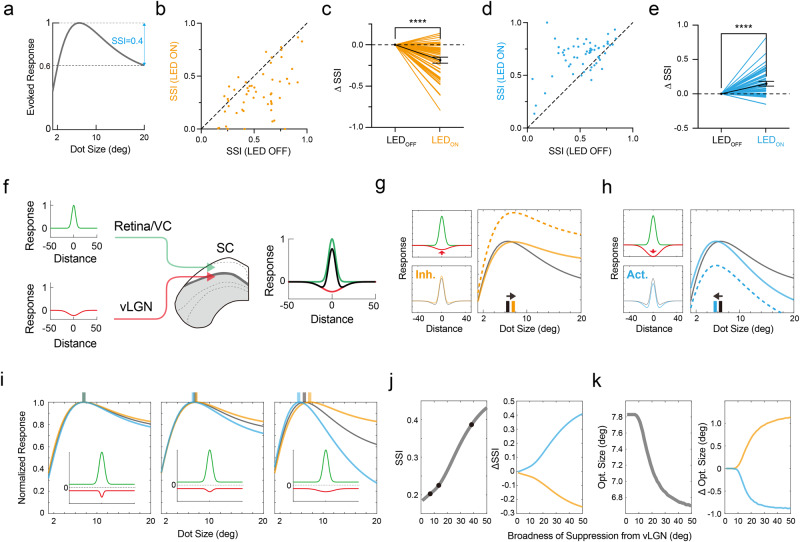
The vLGN input enhances surround suppression in SC. **a** Schematic of calculating surround suppression index (SSI) from the size tuning curve. **b** SSI in LED-ON vs. LED-OFF conditions for photo-inhibition of vLGN VGAT+ neurons. **c** Changes of SSI induced by the photo-inhibition. *n* = 56 neurons, ****p < 0.0001, two-tailed Wilcoxon matched-pairs signed rank test. Bar represents s.e.m. **d**, **e** Similar to (**b**, **c**) but for photo-activation of vLGN VGAT+ axons in SC. *n* = 64 neurons, ****p < 0.0001, two-tailed Wilcoxon matched-pairs signed rank test. **f** Schematic of the difference-of-Gaussian (DoG) model. A SC neuron integrates spatially sharp excitatory input (green curve) from retina and visual cortex and spatially broad inhibitory input (red curve) from vLGN. Black curve (right) represents the linearly summed input. **g** Suppression of vLGN input. Left, spatial tuning curves of excitation (green), inhibition (red) and the integrated response (lower) before (gray) and after (orange) photo-inhibition of vLGN input. Red arrow indicates a reduction of inhibition. Right, simulated size tuning of the SC neuron before (gray) vs. after (dashed orange; solid orange represents tuning curve after normalization) suppressing the inhibitory input. Vertical colored bars indicate the optimal size. Arrow marks the direction of the shift of optimal size. **h** Similar to (**g**) but for photo-activation (i.e., strengthening) of vLGN input (blue). **i** Effects of manipulating the broadness of vLGN input on SC size tuning. Gray, orange and blue represent tuning curves in the control, photo-inhibition and photo-activation conditions, respectively. Colored bars on top indicate the optimal size in different conditions. Inset, spatial tuning of excitation (green) and inhibition (red). **j** Effects of manipulating the spatial broadness of vLGN input on SSI of SC neurons (left) and on the change of SSI induced by photo-inhibition (orange) or photo-activation (blue) of vLGN input (right). The three black dots mark the conditions for the three plots in (**i**). **k** Similar to (**j**) but for effects on the optimal size. Data are presented as mean ± s.e.m. in (**c**, **e**). Source data are provided as a Source Data file.

The effects on size tuning and spatial RFs suggest that vLGN input may modulate the optimal size of SC neurons through a surround suppression mechanism. To evaluate the strength of surround suppression, we calculated a surround suppression index (SSI) (Fig. 6a). The photo-inhibition of the vLGN neurons or their axons in SC decreased the SSI of SC neurons (Fig. 6b, c and Supplementary Fig. 7l, m), while the photo-activation of their axons in SC increased the SSI (Fig. 6d, e). These data support the notion that vLGN input modulates the optimal size of spatial tuning of SC neurons by providing broad surround suppression.

To further illustrate how the broad suppression from vLGN could influence the optimal size of SC neurons, we employed a difference-of-Gaussians (DoG, analogous to “center-surround”) model (Fig. 6f). In this model, the SC neuron receives relatively sharp spatially tuned excitation from the retina and visual cortex. Considering that retinal and cortical inputs received by a SC neuron may vary in their relative strengths^44–46^, we applied spatial parameters of excitation that could vary between properties observed for RGC axons in SC^47^ and for V1 neurons (Supplementary Fig. 4d). As for inhibition, we used the observed vLGN spatial tuning parameters to simulate a broad negative input to SC. These inputs of opposite signs were summed linearly and then went through a nonlinear transformation to simulate the spike response in the target cell (see “Methods”). Firstly, we fixed the excitation and varied the strength of inhibition to simulate optogenetic manipulations of vLGN input. We found that adjusting the level of vLGN input could shift the optimal size while changing response amplitudes. When weakening the vLGN input, the response amplitude of the target cell was generally increased, and the optimal size became larger (Fig. 6g). Increasing the vLGN input, on the other hand, reduced the optimal size and response amplitude (Fig. 6h). These simulation results are in general consistent with our experimental observations. Secondly, we varied the spatial broadness of the vLGN input. When the vLGN input was narrower or similarly broad compared to excitation, manipulating its level had little effect on the preferred size or surround suppression of SC neuron responses (Fig. 6i, left and middle). Only when the vLGN input was spatially broader than excitation, could a shift in preferred size or a robust change in surround suppression be observed (Fig. 6i, right). The influences of the spatial broadness of inhibition from vLGN on SSI and optimal size are shown in Fig. 6j, k. As vLGN inhibition became broader, surround suppression became stronger (Fig. 6j, left), and optogenetic suppression and activation of vLGN input had a stronger effect on reducing and increasing SSI, respectively (Fig. 6j, right). In the meanwhile, the optimal size became smaller (Fig. 6k, left), and optogenetic suppression and activation of vLGN input had a stronger effect on enlarging and reducing the optimal size, respectively (Fig. 6k, right). It is worth noting that the effects on the optimal size tended to saturate when further increasing the broadness of vLGN inhibition, while those on surround suppression did not. Finally, we varied the spatial broadness of excitation and observed qualitatively similar effects with different excitatory tuning profiles (Supplementary Fig. 8). Together, our simulation results demonstrate that the spatially broad inhibition from vLGN can powerfully affect the size preference as well as surround suppression of responses in the target SC neurons.

### Contextual modulation of SC responses by vLGN input

**Fig. 7:**
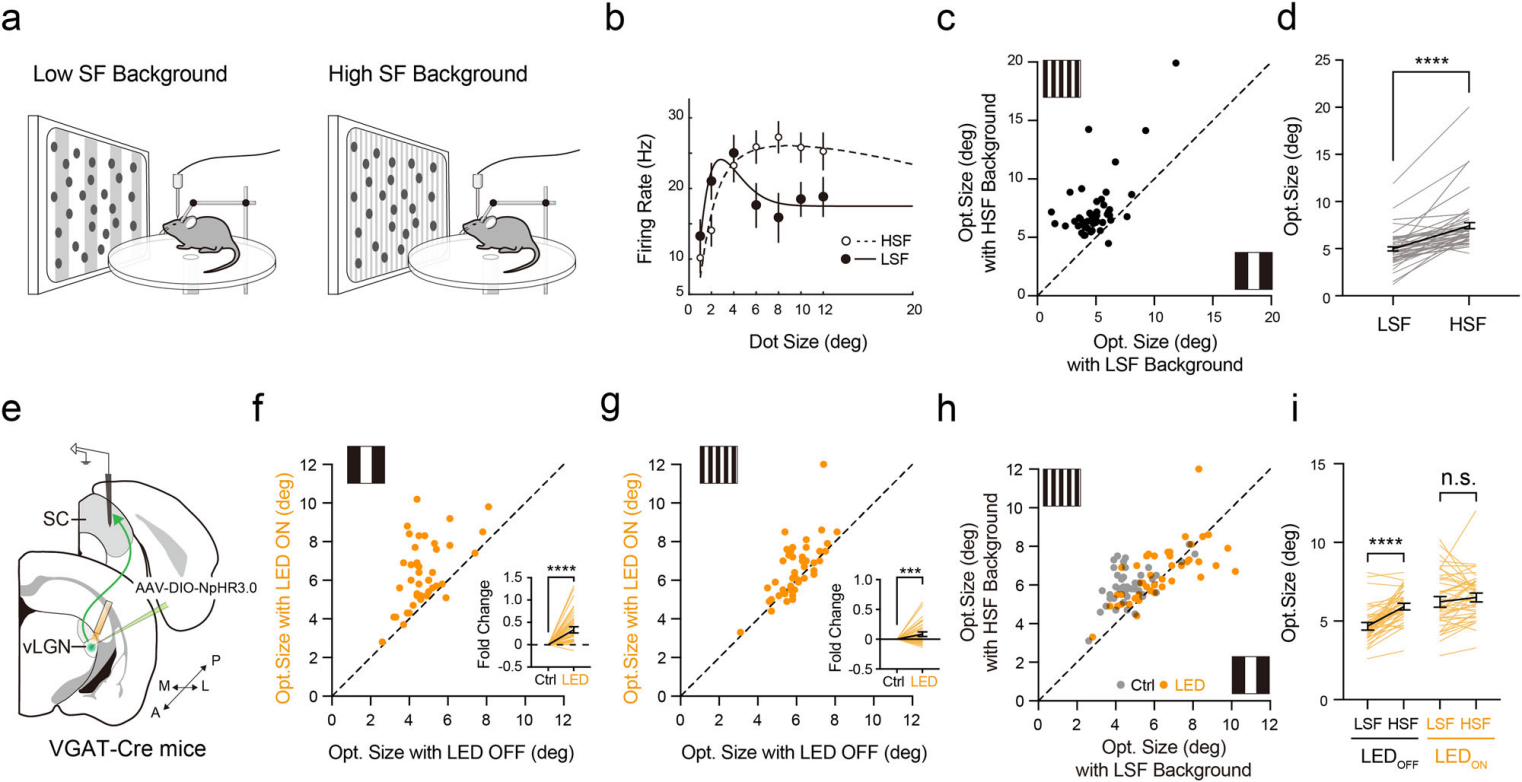
Context-dependent modulation effects of vLGN input. **a** Schematic of SC recording responding to moving dots over low- (left) or high-SF (right) grating background. **b** Size tuning of an example SC neuron under low- (solid line and black dots) or high-SF (dash line and hollow dots) background. *n* = 20 trials. **c** Optimal size in high-SF (HSF) vs. low-SF (LSF) background. **d** Comparison of optimal sizes between the two background conditions. *n* = 64 neurons, ****p < 1.0 × 10^−15^, two-tailed Wilcoxon matched-pairs signed rank test. Black line and bar represent mean ± s.e.m. **e** Schematic of SC recording with photo-inhibition of vLGN VGAT+ neurons. **f** Optimal size in LED-ON vs. LED-OFF condition with low-SF background. Inset, fold change of optimal size. *n* = 44 neurons, ****p = 7.3 × 10^−11^, two-tailed Wilcoxon matched-pairs signed rank test. **g** Similar to (**f**) but for high-SF background. *n* = 44 neurons, **p = 0.0041, two-tailed Wilcoxon matched-pairs signed rank test. **h** Optimal size in high- vs. low-SF background in the LED-OFF (gray) or LED-ON (orange) condition. **i** Comparison of optimal sizes between two SF backgrounds in LED-OFF and LED-ON conditions. *n* = 44 neurons, ****p = 2.9 × 10^−10^; n.s., *p* = 0.6694, two-tailed Wilcoxon matched-pairs signed rank test. Data are presented as mean ± s.e.m. in (**b**, **d**, **f**, **g** and **i**). Source data are provided as a Source Data file.

The broad spatial integration of vLGN neurons suggest that they may modulate SC responses in a context-dependent manner. To test this idea, we measured the size tuning of SC neurons by presenting moving dots over different backgrounds: slow drifting gratings at a low SF or a high SF (Fig. 7a). SC neurons exhibited different size tuning curves in the presence of the different backgrounds (Fig. 7b), with the optimal size significantly smaller under the low-SF than high-SF background (Fig. 7c, d). Since vLGN neurons prefer low-SF stimuli (Fig. 3p), we wondered whether this difference in optimal size could be attributed to a stronger surround suppression effect from vLGN in the low-SF background condition. To address this issue, we compared the optimal size without and with inhibiting vLGN VGAT+ neurons (Fig. 7e). Suppressing the vLGN neurons enlarged the optimal size in both low-SF and high-SF background conditions (Fig. 7f, g), but the difference in optimal size between the two background conditions disappeared (Fig. 7h, i). Similar effects were observed when we specifically silenced vLGN→SC axons (Supplementary Fig. 9). Together, our data support the notion that the vLGN modulation of SC processing is dependent on visual contexts.

### Cortical feedback only slightly enhances the visual response of vLGN neurons

**Fig. 8:**
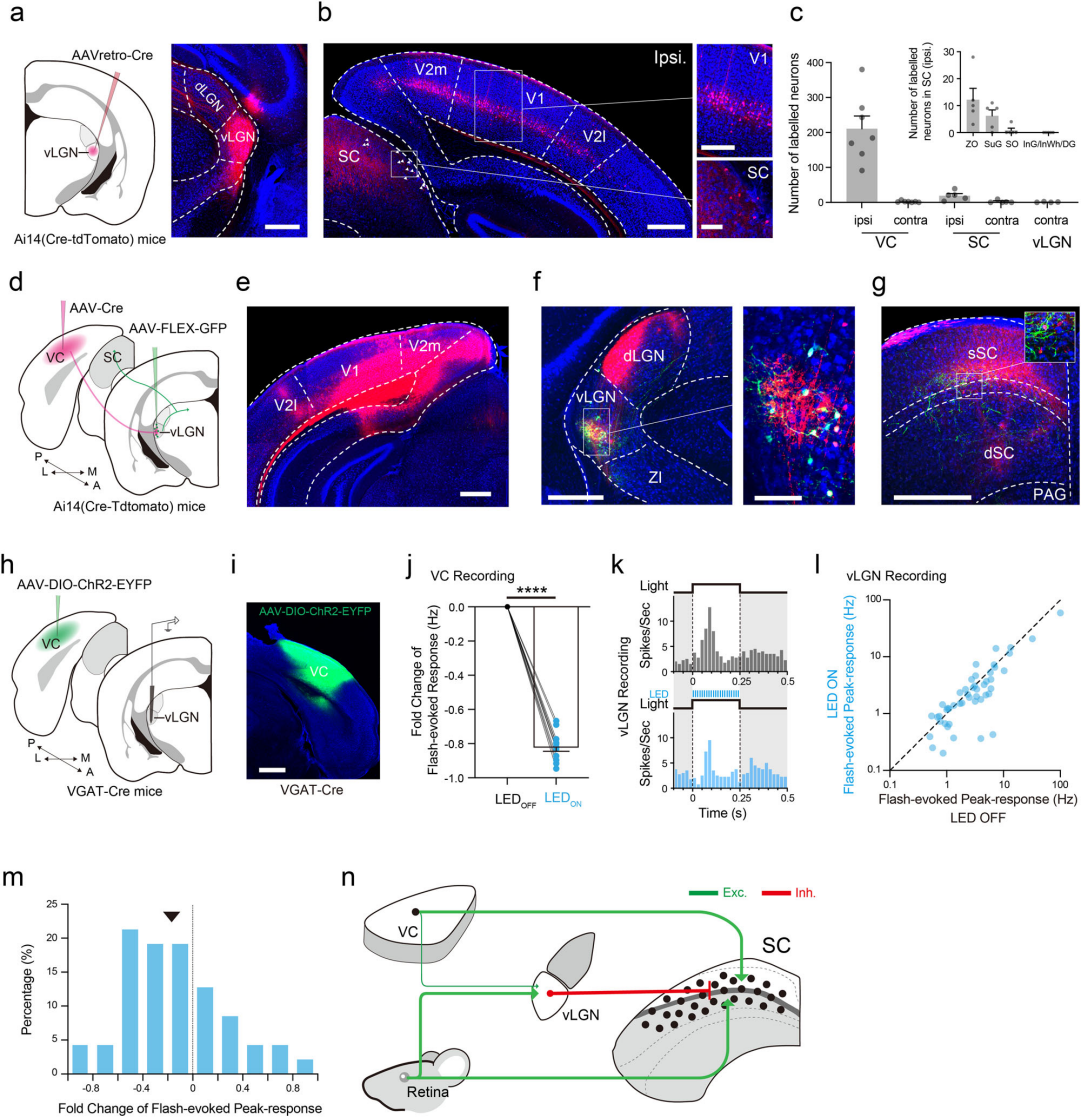
Influence of visual cortical input on vLGN activity. **a** Injection of AAVretro-Cre in vLGN of Ai14 mice. Right, expression at the injection site. Scale bar, 500 μm. **b** Retrogradely labeled neurons in different regions. Scale bar, 500 μm. Right, enlarged boxed areas. Scale bar, 250 μm (upper), 100 μm (lower). *n* = 3 replicates. **c** Numbers of retrogradely labeled neurons. Each dot represents one brain section. *n* = 7 sections for VC, 5 sections for SC, 4 sections for vLGN from 3 mice. Inset, numbers of retrogradely labeled neurons in different sublayers of ipsilateral SC. *n* = 5 sections from 3 mice. **d** Viral injection for transsynaptic tracing of VC-recipient vLGN neurons. **e** Expression at the injection site. Scale bar, 500 μm. **f** Transsynaptically labeled VC-recipient neurons in the ipsilateral vLGN (red and green). Right, enlarged boxed area. Scale bar, 500 μm (left), 100 μm (right). **g** Axon terminals of GFP-labeled VC-recipient vLGN neurons in SC. Scale bar, 500 μm. Inset, enlarged boxed area showing VC-recipient SC neurons in the lower part of sSC. Note that signal intensity was reduced for clarity. **e**–**g** *n* = 3 replicates. **h** Expressing ChR2 in VGAT+ neurons in VC. **i** Example image of ChR2 expression. *n* = 3 replicates. Scale bar, 1 mm. **j** Fold change in visually evoked firing rate of VC neurons induced by photo-stimulation. ****p = 3.0 × 10^−11^, two-tailed paired *t*-test. *n* = 11 neurons from 3 recording sites. **k** PSTHs of responses of an example vLGN neuron to the flash stimulus without (gray) and with (blue) optogenetic silencing of VC. **l** Flash-evoked firing rates in LED-ON vs. LED-OFF conditions. *p = 0.0218, two-tailed paired *t*-test. *n* = 47 neurons. **m** Distribution of fold changes. Arrowhead, median. **n** A circuit model for the long-range feedforward inhibition from retina through vLGN to SC. The retina-vLGN-SC pathway delivers broad feedforward suppression to SC neurons in parallel with retina-SC and VC-SC excitatory circuits. Green and red represent excitatory and inhibitory projections, respectively. Data are presented as mean ± s.e.m. in (**c** and **j**). Source data are provided as a Source Data file.

The vLGN receives excitatory input from visual cortex (VC) besides the retina^1,7,48^. We wondered whether this could contribute to the geniculo-collicular visual pathway. To answer this question, we first injected AAVretro-Cre into the vLGN of Ai14 (Cre-dependent tdTomato) mice and retrogradely labeled the inputs to vLGN (Fig. 8a). Consistent with previous studies^1,7,48^, we observed abundant vLGN-targeting neurons in deep layers (primarily layer 5) of visual cortical areas including V1 and V2 (Fig. 8b). Sparsely labeled neurons were also observed in the most superficial layers of SC (Fig. 8b, lower right) including the zonal layer (ZO) and superficial gray (SuG) (Fig. 8c, inset), confirming that the observed labeling of neurons in SO and InG by AAV1-Cre injection in vLGN (Fig. 4p) was due to bona fide anterograde transsynaptic labeling. Quantitation of labeled cell numbers showed that the ipsilateral VC is a prominent input source other than the retina (Fig. 8c). Next, we labeled cortico-recipient vLGN neurons by injecting AAV1-Cre into the VC and AAV encoding Cre-dependent GFP into the vLGN of Ai14 mice (Fig. 8d, e). The axons of those GFP-labeled cortico-recipient vLGN neurons (Fig. 8f) projected into SC, most strongly in the lower portion of sSC, where cortico-recipient SC neurons were most densely distributed, and to a lesser degree in the intermediate layers of SC (Fig. 8g). Thus, vLGN neurons can relay visual information partially from the VC to SC. To test the influence of this cortical feedback on vLGN responses, we expressed Cre-dependent ChR2 in the VC of VGAT-Cre mice^49–51^ (Fig. 8h) and compared flash-evoked responses of vLGN neurons without and with photo-inhibition of VC. We found that the optogenetic silencing of VC (Fig. 8i, j) slightly but significantly reduced the visually evoked response in vLGN (Fig. 8k–m). This moderate effect appears different from a recent slice recording study showing that retinal and cortical inputs to a subpopulation of vLGN neurons are potentially comparable in strength^48^, but suggests that the major visual input to drive the geniculo-collicular pathway is most likely from the retina, at least in our in vivo experimental conditions.

## Discussion

The vLGN is the major thalamic input source of SC^10^, with dLGN not projecting to SC. In contrast to geniculostriate and tectopulvinar visual pathways, the thalamocollicular pathway via vLGN has been poorly investigated. It is characterized by direct and vast retinal inputs^5,6,48,52^, substantial proportion of inhibitory projection neurons within vLGN^7–9^ and their specific influence on visual neurons in SC^19^. As part of the caudal prethalamus, vLGN has been implicated in an inhibitory switchboard for behavioral control^53^. In the present study, we have discovered a previously unrecognized role of the vLGN GABAergic projection in shaping the visuospatial processing of SC neurons. Our data demonstrated that vLGN neurons have very broad spatial receptive fields, low preferred spatial frequencies and poor orientation/direction selectivity. Through a surround suppression mechanism, the vLGN projection to SC can shift the preferred spatial frequency and optimal size of SC neurons, allowing them to better process higher spatial frequency information and smaller stimulus sizes. Furthermore, we found that the surround suppression effect of the vLGN input is transformed into its influence on a SC-dependent visually guided approaching behavior, i.e., to facilitate detection of small moving objects. Our results have thus provided concrete evidence that the long-range GABAergic thalamocollicular pathway can play an important role in fundamental visual information processing in SC-mediated visuomotor behaviors.

Since vLGN receives substantial direct input from the retina^5,6,48,52^, it has long been known to respond well to visual stimuli^35,36^. Previous studies have reported a notable bias of vLGN responses toward luminance increases or ON stimuli^35,37^, yet with a minority of neurons responding to OFF stimuli^6,34,36^. Tonic, phasic or both patterns of responses can be observed in vLGN responding to ON or OFF flash stimuli. Nevertheless, this structure has long been thought to play a major role in encoding luminance based on the observations that responses of “On-tonic” cells are well correlated with illuminance levels^1,7,36,37^ and that lesion of vLGN impairs the performance of discrimination of light intensities^54^. In the current study, we confirmed the ON-bias in vLGN responses but observed even more diverse response patterns, especially there existing a large number of ON-OFF cells. Therefore, the majority of vLGN neurons can respond to both ON and OFF stimuli. In addition, we found that vLGN neurons were robustly activated by moving dots and tuned to large dot sizes (Supplementary Fig. 6). These observations indicate that vLGN can encode diverse visual information far beyond illuminance levels. This notion is consistent with the fact that vLGN receives inputs from multiple types of retinal ganglion cells^52^, as well as from subcortical/cortical visual areas such as SC, V1 and V2^1,7,48^ (Fig. 8b).

Despite the fact that both the vLGN and dLGN receive prominent retinal inputs, vLGN neurons are distinct from dLGN neurons^55^, especially at the aspect of spatial tuning (Fig. 3o, p). Previously, it has been reported that visual RFs of vLGN neurons tend to be larger than dLGN neurons in various species such as rabbits^35^, cats^36^, rats^37^ and mice^6^. Our quantitative measurements of RF sizes confirmed that vLGN neurons had much broader RFs than not only dLGN but also SC neurons. This broader spatial integration is also reflected by their lower preferred SFs and larger optimal sizes in responding to grating and moving dot stimuli, respectively. As demonstrated by our modeling results (Fig. 6i–k), the broad spatial tuning of vLGN input is critical for enhancing the surround suppression in SC: increasing the SSI and reducing the optimal size of SC neurons. Morphological properties in that dendric arbors of individual vLGN neurons cover a much larger breadth than dLGN neurons^6^ can partially explain this feature of vLGN neurons. The RGCs projecting to vLGN and dLGN are distinct to some extent^2,52,56^, which might also contribute to the differential spatial processing features of the two thalamic areas.

It is worth mentioning that our current results have some discrepancies with previous studies. Anatomically, our anterograde tracing reveals projections of vLGN VGAT+ axons more strongly to the superficial layers of SC (Fig. 1k), whereas two previous studies^18,19^ focusing on the medial SC (mSC) demonstrate the strongest vLGN GABAergic projections to intermediate/deep layers (i.e., motor SC). However, one of these studies^18^ has shown weak projections to intermediate/deep layers in the lateral part of SC while relatively strong projections to the superficial SC, which is somewhat consistent with our results. It should be noted that vLGN is a vastly heterogenous brain region containing multiple sublaminae with enriched molecularly distinct GABAergic cell types^8^. Different experimental parameters such as the angle, depth and location of the injection pipette can affect the subpopulations of vLGN neurons infected. In addition, two areas neighboring vLGN, the intergeniculate leaflet (IGL) and ZI, contain GABAergic neurons which also project to SC^10,53^. In particular, IGL appears to provide some projections to the superficial SC^19^. Contamination of either of these areas may not be avoidable when using VGAT-Cre mice. This can also contribute to the variability in anatomical results among different studies. Nevertheless, we observed the strongest vLGN suppressive effects in SO and immediately below (Fig. 4e) and the densest labeling of vLGN-recipient neurons in SO (Fig. 4o), consistent with the specific vLGN influence on visual SC neurons^19^.

Differences in experimental parameters may also explain discrepancies at the functional level. For example, we found a larger proportion of On-Off vLGN neurons than previously observed^6^. In addition, while the two previous studies suggest that suppression of vLGN neurons increases anxiety and risk-avoidance behavior^18,19^, our real-time place preference test did not generate evidence that short-term inhibition of vLGN is associated with negative emotion-related aversion. As how vLGN and its neighboring structures such as IGL and ZI innervate SC circuits is not yet fully understood^53^, more future studies are needed to resolve these discrepancies, perhaps by using more refined cell-type-specific Cre lines or projection-based approaches.

Modulation of response properties by surround suppression is a fundamental mechanism throughout the visual system. It can be achieved through various combined circuit mechanisms including feedforward, lateral and feedback mechanisms^57^. A notable example is the corticothalamic feedback mediating surround suppression in dLGN via the thalamic reticular nucleus (TRN): by exciting inhibitory neurons in the visual sector of TRN, corticothalamic neurons in V1 layer 6 can provide indirect inhibition onto thalamic relay cells to enhance surround suppression in these cells^58^. In the cortex, besides the feedforward geniculostriate pathway^57^, the tectopulvinar pathway has also been found to contribute to surround suppression there by driving layer 1 inhibitory neurons^59^. In the present study, our results have revealed a previously unrecognized role of the thalamocollicular pathway in providing surround suppression to SC neurons. As proposed previously^53^, the GABAergic neurons in vLGN receive retinal input and send inhibitory output to SC, constructing a long-range feedforward inhibitory circuit (Fig. 8n). Due to its broad spatial integration property, the vLGN input can modulate the level of surround suppression and the spatial preference of SC neurons. In addition, vLGN neurons have poorer orientation selectivity than dLGN and SC neurons, allowing their input to enhance orientation selectivity of SC neurons through broad inhibition (Supplementary Fig. 7a–f). Importantly, the vLGN activity is sensitive to the visual context and thus provides a context-dependent modulation. While a previous imaging study has suggested that long-range lateral inhibitory inputs within sSC contribute to the surround suppression there^60^, based on our results, we propose that the GABAergic thalamocollicular pathway via vLGN might be an important external inhibitory input source contributing to the surround suppression in SC.

Surround suppression serves an important role in visual information processing. It has been shown to enhance neuronal feature selectivity such as orientation and direction selectivity^59,61^, as well as to facilitate figure-ground segregation^62^ and extraction of object boundaries^63^. Surround suppression can also increase the sparseness of stimulus representation and improve the efficiency of information transmission under natural scenes^64^. Consistent with these positive cognitive effects, we find that the vLGN input to SC can improve the animal’s ability to detect small moving objects. At the neuronal response level, activation of the vLGN input shifts the SC size preference toward a smaller size. This may allow recruitments of more SC neurons by a smaller stimulus size, even though the evoked firing rate of individual neurons might be reduced. In addition, the surround suppression can enhance the contrast of stimulus edges^65^, thus facilitating detection. Such effects together may contribute to the enhanced sensitivity to small moving objects of particular sizes when activating vLGN, resulting in improved behavioral performance. While our results are apparently different from the two previous studies on medial-SC-dependent defensive behavior in that activation of vLGN suppresses defensive reactions^18,19^, they are reminiscent of previous findings of a critical involvement of SC wide-field (WF) neurons in prey detection during prey capture^21,22^. The WF neurons are located roughly in SC sublaminae^51,66^ where the vLGN input exhibits the strongest inhibitory modulation (i.e., SO and immediately below). The large visual RFs of SC neurons at these laminar depths (Fig. 4l) also support that they are likely WF cells. Therefore, we postulate that the vLGN input can strongly modulate the activity of WF neurons, affecting the behavioral detection of small objects.

Altogether, we propose a circuit model (also see ref. ^53^) for the inhibitory modulation of SC visual activity by a retino-geniculo-collicular pathway mediated through vLGN (Fig. 8n). As a key node in this pathway, vLGN receives visual signals majorly from retinal input, while it can also receive some signals from visual cortices and subcortical nuclei. Visual processing in SC is then driven by excitatory retinal and cortical inputs^45,46^ and modulated by the inhibitory input from vLGN. Our study suggests that this external long-range, feedforward-like inhibitory circuit from the retina to midbrain serves to ensure a fast and powerful modulation of visuospatial integration/processing in SC and the related visuomotor behaviors.

## Methods

The surgeries and experiments were performed in the Zilkha Neurogenetic Institute (ZNI) at the University of Southern California (USC). Institutional Animal Care and Use Committee (IACUC) of USC approved all procedures used in this study.

### Animals

Male and female wild-type C57BL/6J and transgenic Vgat-IRES-Cre (Jackson Laboratories, RRID: IMSR_JAX:016962), Ai14 (Jackson Laboratories, RRID: MSR_JAX:007914) and Ai75 (Jackson Laboratories, RRID: IMSR_JAX:025106) were used in this study. Male and female adult (8–12 weeks old) mice were randomly assigned in electrophysiological recordings and anatomical experiments. Similar numbers of male and female mice were assigned in the same behavioral experiment. The animals were housed at 18–23 °C with 40–60% humidity in a 12-h light-dark cycle (6AM-6PM light).

### Viruses

AAVretro-Cre (1.5 × 10^14^ GC/ml, Vigene), AAV2/1-pEF1a-DIO-hChR2-eYFP (1.82 × 10^13^ GC/ml, UPenn vector core), AAV1-hSyn-Cre-WPRE (2.5 × 10^13^ GC /ml, UPenn vector core), AAV1-CAG-FLEX-GFP-WPRE (2 × 10^13^ GC/ml, UPenn vector core, Addgene 51502), AAV1-CAG-mCherry (4.8 × 10^13^ GC/ml, UPenn vector core), AAV9-EF1a-DIO-eNpHR3.0-EYFP (1.7 × 10^13^ GC/ml, UPenn vector core, Addgene 26966) and AAV2-hSyn-hM4D(Gi)-mCherry (3 × 10^13^ GC/ml, UPenn vector core, Addgene, 50475) were used in this study.

### Surgical procedures

Stereotaxic viral injections were performed at least 3 weeks prior to electrophysiological or behavioral tests. General anesthesia was induced (3%) and maintained (1–1.5%) with isoflurane in oxygen. Then the mouse was fixed on a stereotaxic frame (Kopf Instrument). After the head was cleaned and shaved, lidocaine gel (2%) was applied, and a small cut was made on the skin. Two craniotomy holes were drilled with a microdrill (RWD) in the skull above the target regions, vLGN (AP −2.5 mm, ML ±2.6 mm, DV −2.8 mm) or SC (AP −4.0 mm, ML ±1.0 mm, DV −0.8 mm). To silence cortical inputs, multiple injections were performed in the ipsilateral VC (AP −3.0/−4.0 mm, ML +2.5/+3.0 mm, DV −0.5 mm). Viral vectors were injected through a glass micropipette and controlled by an injector (Microinjection Syringe Pump, WPI) at a speed of 25–30 nl/min. Except for anatomy, all the viral injections were performed bilaterally. The volume for each injection was 50–200 nl based on the size of the target region. At least 10 min after the injection, the glass micropipette was retracted slowly. When all the injections were finished, the scalp was sutured. Buprenorphine (Sustained-Release, 0.1 mg/kg, once) and Ketoprofen (0.5 mg/kg, once per day in the following 3 days) was injected subcutaneously after the surgery. When recovered from anesthesia, the mouse was returned to its home cage and monitored in the following 3 days. The suture was removed 10–14 days after the surgery. After all the experiments, mice were euthanized by Euthasol (i.p.) injection followed by cervical dislocation to verify the viral expression and location.

For the optogenetic modulation in behavioral tests, optic cannulas were implanted into the target regions bilaterally 2 weeks after stereotaxic viral injections. The surgery procedure was similar to viral injection. Under the anesthesia, optical cannulas (NA: 0.39, RWD) were implanted bilaterally into the target regions, vLGN (AP −2.5 mm, ML ±3.3 mm, DV −2.2 mm, with a 20° angle to avoid dLGN) or SC (AP −4.0 mm, ML ±1.0 mm, DV −0.4 mm). The optical cannulas were fixed with dental cement. To avoid the light from optical cannulas into eyes, we applied black dental cement (Lang Dental, cat. no. 0206, 1506) on the surface of fixed cannulas and solidified the cement. After the surgery, the animal was allowed to recover for 1 week before behavioral tests.

For electrophysiological recordings in vivo, a preparation surgery was performed 3 days prior to the recording session. The surgery procedure was similar to viral injection. Under the anesthesia, a metal post for head fixation during the following recording experiment was mounted onto the skull of the animal and fixed with dental cements. A craniotomy was performed over recording regions (LGN, V1, or SC). After the surgery a silicon elastomer (Kwik-CAST, WPI) was applied to cover the surgical opening before recording experiments. For the recordings with optic modulation of V1 or vLGN, two optical cannulas were implanted into VC (AP −3.0/−4.0 mm, ML +2.5/+3.0 mm, DV −0.5 mm) or one in vLGN (AP −2.5 mm, ML ±3.3 mm, DV −2.2 mm, with a 20° angle to avoid dLGN) and fixed with dental cements during the surgery.

### Visual stimuli

The visual stimuli were generated by custom-made scripts using Psychophysics Toolbox (Psychtoolbox-3, PTB-3) in MATLAB (MathWorks), and displayed on a gamma-corrected LCD monitor (37 cm × 30 cm, ViewSonic, vp950b) refreshing at 60 Hz and positioned 17 cm from the animal eyes (~120° × 100° in the visual field). Luminance level was 22 ~ 27 cd/m^2^ in our experiments. A photosensor was used to record the visual stimuli for post hoc alignment and a data acquisition board (USB6001, National Instruments) was used to control the LED precisely with the visual stimuli. When no visual stimulus was given, a uniform gray screen of average luminance was presented as a blank stimulus. The visual flash stimulus which consisted of a 90-deg diameter white or black disc was presented for 250 ms with 250 ms intervals and 200 repetitions. The sparse noise stimulus was used to map the neuronal visual receptive field, in which one white or black square (10° or 20°) were conventionally presented for 250 ms with 250 ms intervals and 8 repetitions at each of 16 × 16 locations (5° spacing) on the screen in a pseudorandom sequence. Drifting gratings were full screen square or sinusoidal gratings drifting in 12 directions in a pseudorandom sequence. The drifting gratings were presented for 1 s with 1 s intervals and 20 repetitions. A set of drifting gratings with various spatial (0.01, 0.015, 0.02, 0.05, 0.1, 0.2 cycle/°) or temporal (0.5, 1, 2, 5, 10 cycle/s) frequencies were applied to measure neuronal preferred SF or temporal frequency. These drifting gratings were presented for 1–2 s with 1 s intervals and 10 repetitions. In the moving dots stimuli, 20–200 black patches of diverse sizes (1°, 2°, 4°, 6°, 8°, 10°, 20° diameter) moved in the horizontal directions (0° or 180°) at the speed of 50°/s and each condition was repeated 20 times. The dots kept static during the initial 200 ms and drifting in the following 500 ms with a following 500 ms blank. The densities of patches were adjusted based on their sizes to ensure a relatively constant luminance level. In the moving dots stimuli with different backgrounds, 0.4-contrast drifting gratings of 0.01 or 0.1 cycle/° and 2 cycle/s were added behind the moving dots. Foreground and background stimuli were presented simultaneously but moving in opposite directions.

In the innate approaching tests, one horizontally moving dot was presented on the screen in one of two end zones randomly chosen. The moving dot was presented 2 cm above the floor moving horizontally within a 7-cm range at a speed of 50°/s continuously until a “Correct” or “Incorrect” choice was made, or at the end of the test window (2 min). For trained approaching, during the training sessions, the size of the moving dot was kept constant (6° diameter) and randomly presented in one of the two end zones, while in the test session, size of the moving dot was varied (diameter: 1°, 2°, 3°, 4°, 5°, 6°, 7°) in a pseudorandom sequence.

### Electrophysiological recordings

Multichannel recording was performed in awake head fixed animals using the Open Ephys acquisition system. Mice were habituated to the head-fixation prior to recordings. For each animal, 1–2 recording sessions were conducted every day with each session lasting for no more than 1 h. Before the recording, the silicon seal was removed. A 64-channel silicone probe (NeuroNexus) was used to penetrate into the desired brain region. The signals were recorded at 30 kHz sampling rate and passed through a bandpass filter (0.3–3 kHz). Single-unit spikes were obtained by a semiautomatic spike sorting using Offline Sorter (Plexon), following our previous studies^59,67–69^. Single-unit activities were analyzed with customized scripts in MATLAB. After all recording sessions, a non-toxic fluorescent and lipophilic dye, DiI, was painted onto the electrode which was then inserted back into the recording location. At last, mice were euthanized to verify the recording location. For optrode recordings or the electrophysiological recordings with optogenetic modulation, fiber-coupled LEDs (Thorlabs) were used as light source and the light was delivered to the optrode or the optic cannula implanted in the brain. The LEDs were controlled by a customized LabVIEW program (National Instruments).

### Histology

Animals were deeply anesthetized using isoflurane (3–5%) and transcardially perfused with PBS and 4% paraformaldehyde (PFA). Brains were post-fixed overnight. Coronal brain sections (150 μm) were made using a vibratome (Leica Microsystems) and stained with a fluorescent Nissl stain (NeuroTrace 435/455, Thermo Fisher Scientific) at 4 °C overnight. Under a confocal microscope (Olympus), the brain sections were imaged to check the expression of virus, the location of electrode recordings, fiber implantations and viral injections, and the anterograde or retrograde tracing.

### Behavioral tests

All behavioral tests were conducted in a sound attenuation booth during the dark cycle of the mice.

#### Two-chamber real-time place preference (RTPP) test

To assess the functional effects of vLGN neurons on locomotion and emotion, mice were subjected to the place preference test with activating or silencing vLGN. The mouse was placed into a clear acrylic behavior box (45 cm × 30 cm × 30 cm) which was divided into two chambers. Behavioral data was recorded with a camera mounted above the box. LED was automatically close-loop controlled by customized software in Python 3.4 that detects the location of the animal in real-time^69,70^. For each trial, the mouse was initially placed in the non-stimulation chamber (LED OFF), and once the animal entered the stimulation chamber, blue light pulses (480 nm, 20 Hz, 5-ms pulse duration) were delivered to activate vLGN or continues amber light (590 nm) was turned on to silence vLGN. LED stimulation was terminated when the animal exited the chamber. The total duration of each test session was 20 min. Animals were returned to their home cage after each test session. The stimulation chamber was randomly assigned to each animal and balanced for the whole group.

#### Visually guided approaching test

To assess the functional role of vLGN in visual information processing, mice were subjected to a visually guided approaching test in an open-top acrylic box (30 cm × 60 cm × 30 cm). At one end of the acrylic box, it was divided into two chambers with a nontransparent acrylic board. All the walls of the acrylic box were nontransparent except the one at the end of the two chambers. A monitor was attached to the transparent wall to present the visual stimuli and the luminance level was kept at 20 ~ 27 cd/m^2^ in approaching tests. One camera was mounted above the box to monitor the locomotion of the mouse.

In the innate approaching behavioral tests, naïve mice were put into the box and habituated for 5–10 min prior to the test. Then, a 5° diameter black dot was presented in one of the two end zones randomly and moving horizontally within a 7-cm range at a speed of 50°/s once the mouse was moving into the initiation zone and facing the monitor. The choice of the animal was determined by the end zone where its whole body firstly entered. The visual stimulus was terminated as long as the animal entered either of the two end zones. If the mouse firstly entered the dot-presenting end zone, it would be a “Correct” trial. If the mouse entered the other end zone, it would be a “Incorrect” trial. If it did not enter either of the two end zones within 60 s, it would be scored as a “No-choice” trial and the visual stimulation was terminated. In the sham group, no visual stimulus was presented in the tests and we did similar quantifications of the three behavioral results. Thirty-eight mice (20 females and 18 males) were examined, and 23 mice (13 females and 10 males) were presented with the sham stimulation. To test the influence of dot contrast or size, different groups of mice (for dot contrasts, 14 females and 15 males; for dot sizes, 30 females and 27 males) were assigned to test dots of different contrasts and sizes, respectively. Tests for the same animal were performed in different days to avoid adaptation confound. To test the influence of chemogenetic silencing of SC, the same group of mice were tested with 5° diameter dot without and with i.p. injection of saline or CNO (20 min prior to the test) in different days. To test the influence of optogenetic silencing of vLGN or vLGN-SC axons, the same group of mice were tested with LED On or Off in different days. The test sequence was randomized with at least 24 h intervals. To investigate the habituation of innate approaching behavior, four consecutive trials with ~2-min intervals were performed in each animal.

In the trained approaching behavioral tests, a thin tube was added into each end zone to deliver water to the animal. After water restriction for about 36 h, the animal was then put into the box and performed the training session. Similarly, when the mouse moved into the initiation zone and was facing the monitor, a moving dot was presented on the monitor screen in one of the two end zones. If the mouse firstly entered the dot-presenting end zone, a drop of water (~20 µl) was delivered, visual stimulus was terminated, and it would be scored as a “Correct” trial. If not, no water was delivered and a timeout would be applied. After each Correct or Incorrect trial (plus timeout), the mouse had to return to the initiation zone and face the screen as to start the next trial (i.e., self-initiated). In each training day, the training session was continued until the number of Correct trials reached 50. The mouse therefore received about 1 ml water in total each day during training. When the success rate of the animal was higher than 80% in three consecutive days, the animal would be regarded as well trained. At the beginning of training, it usually took around 30 ~ 60 min for a session. Over days, training would take less trials and shorter time. For a relatively well-trained animal, one training session was usually completed within 60 trials (i.e., 60 stimuli), which only took ~15 min. For the test session, moving dots of seven different sizes were presented to the animal in a pseudorandom sequence and each condition was repeated at least eight times. The test trials with LED On and Off were performed in different days with at least 24 h interval.

### Optogenetic and chemogenetic manipulations

For the electrophysiological recording experiments, optical cannulas (Core diameter: 200 µm, NA: 0.28, RWD) were implanted into the target region 3 days prior to the recording session. During the recordings, the optical cannula was connected to a LED light source (470 or 590 nm, Thorlabs). The light power was set to be about 3–5 mW (measured from the fiber tip). To activate neuronal activity using ChR2, blue light pulses (470 nm, 5-ms pulse duration at 20 Hz) were delivered through the implanted fibers. To suppress neuronal activity using NpHR3.0, continuous amber light (590 nm) was delivered simultaneously with the visual stimulation. For the behavioral experiments, optical cannulas were implanted into the target region at least 1 week prior to the behavioral tests. Animals were habituated to the optic cable in the test box at least 2 days prior to the behavioral tests. In the electrophysiological recording experiments, the blue or amber light was started at the same time with visual stimuli and stopped when visual stimuli ended. In the activation experiments of behavioral tests, the blue LED was started when the moving dot was presented and stopped when visual stimulation was stopped. In the inhibition experiments of behavioral tests, the amber LED was started 0.1 s prior to the onset of visual stimulation. The LED stayed ON until the animal completed one approach trial (when visual stimulation also stopped), or for the whole duration of test window. For chemogenetic stimulation, animals expressing hM4D(Gi) received intraperitoneal injection of clozapine-N-oxide (CNO) (1 mg/kg) (or saline in control trials) 30 min before the behavioral tests.

### Computational model

To explore how the size tuning of SC neurons was shaped by the broad suppression from vLGN, we used a “difference-of-Gaussians (DoG)” model to simulate the activation and silencing of vLGN activity. In the DoG model, the SC neuron’s spatial tuning depended on a spatially sharp excitatory input and a broad inhibitory input. We applied excitatory spatial profiles that varied between experimentally observed spatial tuning profiles for RGC axons in SC and V1 neurons. As for inhibition, we used the observed spatial tuning parameter of vLGN neurons to simulate a broad negative input to SC. Through a linear summation of the two weighted inputs followed by a non-linear transformation, we obtained an integrated spatial tuning profile that was then fitted with the measured data recorded in SC. Thus, we generated a model which could integrate the two inputs and generate the size tuning of the output:

1 $${S}_{{E\; {{{{{\rm{or}}}}}}\; I}}=\frac{{{{{{{{\rm{RF}}}}}}\; {{{{{\rm{size}}}}}}}}_{E{{{{{{\rm{or}}}}}}}\, I}}{\sqrt{8{{{{\mathrm{ln}}}}}2}}$$

2 $$y={a}_{E}*{e}^{{\left(-\left(\frac{x}{{S}_{E}}\right)\right)}^{2}}-{a}_{I}*{e}^{{\left(-\left(\frac{x}{{S}_{I}}\right)\right)}^{2}}$$

3 $${y}_{{{{{{{\rm{out}}}}}}}}={p}^{\, y}+q$$

where ${{{{{{{\rm{RF}}}}}}\; {{{{{\rm{size}}}}}}}}_{{E\; {{{{{\rm{or}}}}}}\; I}}$ is the RF size of center excitation or surround suppression, which was based on the measured data of RGC axons in SC^47^, V1 neurons or vLGN neurons. ${S}_{{E\; {{{{{\rm{or}}}}}}\; I}}$ is the transformed sigma of the two Gaussians curves. ${a}_{E}$ and ${a}_{I}$ are the weights of the excitatory and inhibitory inputs, respectively. $p$ and $q$ are the variables in the nonlinear function, and ${y}_{{{{{{{\rm{out}}}}}}}}$ is the final spatial response curve which produced size tuning that fit well to the measured data of SC neurons (*r*^2^ ≥ 0.99).

The activation and silencing of vLGN were simulated by adjusting the weight of inhibitory input, which was determined by our observed vLGN activation and silencing effects on the evoked-responses of SC neurons.

### Data analyses

Animal tracking in the behavioral tests was performed with customed Python scripts^68–70^. Raw electrophysiological data were saved online, and spikes were detected offline using a thresholding algorithm. Detected spikes were then sorted using valley seeking and principal components analysis. All further analyses were conducted with customized scripts in MATLAB.

#### Spike sorting

Spike sorting was performed following our previous studies^59,67–69^. The raw signals from the 64-channel silicon probe were filtered through a 300–3000 Hz band-pass filter. The nearby four channels of the silicon probe were grouped as tetrodes. The spatially varying motion artifacts were removed by applying a local common average referencing (L-CAR) scheme. Spike detection and sorting was performed using the Plexon offline sorter (Dallas, Texas). Clusters with isolation distance >20 was considered as separate clusters. Spike clusters would be classified as single units only if the waveform SNR (Signal Noise Ratio) exceeded 4 (12 dB) and the inter-spike interval was longer than 1.2 ms for more than 99.5% of the spikes.

#### Visually evoked response

To classify the neuronal visual response pattern and quantify the response level, a light-ON or -OFF response was calculated based on the spikes within the 100–250 ms window after the onset of visual stimuli. The spontaneous activity within the 0–100 ms window before the onset of visual stimuli was subtracted. The unit of which visually evoked response was smaller than 1 spike/s was excluded in further analyses. The ratio of the evoked firing rate to the spontaneous firing rate was used to evaluate the responsivity. The ratio great than 0.5 was defined as a positive response, while the ratio less than −0.5 was defined as a negative response. The ratio between −0.5 and 0.5 was defined as non-responsive.

#### ON-OFF bias index

To quantify the neuronal preference to ON or OFF stimuli, we defined an On-Off bias index as follows:

4 $${{{{{{\rm{On}}}}}}}-{{{{{{\rm{Off}}}}}}}\,{{{{{{\rm{Bias}}}}}}}\,{{{{{{\rm{Index}}}}}}}=\frac{{R}_{{{{{{{\rm{ON}}}}}}}}-{R}_{{{{{{{\rm{OFF}}}}}}}}}{{R}_{{{{{{{\rm{ON}}}}}}}}+{R}_{{{{{{{\rm{OFF}}}}}}}}}$$

where ${R}_{{{{{{{\rm{ON}}}}}}}}$ and ${R}_{{{{{{{\rm{OFF}}}}}}}}$ represent firing rates evoked by ON and OFF stimuli, respectively.

#### Receptive fields (RFs) mapping

ON and OFF RFs were obtained based on the visually evoked responses to white and black stimuli, respectively. The evoked responses to the white or black square at each location on the screen were averaged across trials and then divided by their standard deviations to obtain the spatial map of evoked response *Z* score. Then, a 2D Gaussian filter was applied to the spatial maps to smooth the RF maps and RFs were defined as the area where the *Z* score was greater than 1. To better measure the size of RFs, the thresholded RF maps was fitted with a 2D elliptical Gaussian function. The sizes of RFs were quantified from the area of the fitted RF maps, which was transformed into a circle with the same area. The radius of the circle was defined as the RF size.

#### Spatial frequency tuning

The preferred spatial frequency was obtained by fitting Gaussian functions of logarithmic spatial frequency to each cell’s evoked responses to drifting gratings of different spatial frequencies as follows:

5 $${{{{{{\rm{Res}}}}}}}(x)=a{e}^{\big(-{\big(\frac{{\log }_{2}(x/0.01)-b}{c}\big)}^{2}\big)}$$

where *x* is the spatial frequency and Res(*x*) is the firing rate to the drifting grating of the spatial frequency. The preferred spatial frequency is calculated as follows:

6 $${{{{{{\rm{Pref}}}}}}}.{{{{{{\rm{SF}}}}}}}={0.01*2}^{b}$$

The bandwidth of spatial frequency tuning was measured as follows:

7 $${{{{{{\rm{SF}}}}}}}\,{{{{{{\rm{tuning}}}}}}}\,{{{{{{\rm{width}}}}}}}=2\sqrt{{{{{\mathrm{ln}}}}}2}*c$$

#### Direction and orientation selectivity

Direction tuning curves were measured based on neuronal evoked responses to drifting gratings at 12 directions within the 100–1000 ms window after the onset of visual stimuli and the spontaneous activity within the 0–500 ms window before the onset of visual stimuli was subtracted. Orientation tuning curves were then obtained by averaging the evoked responses to two opposite directions. To quantify the direction and orientation selectivity, we applied the conventional DSI and OSI as follows:

8 $${{{{{{\rm{DSI}}}}}}}=\frac{{{{{{{{\rm{RD}}}}}}}}_{{{{{{{\rm{pref}}}}}}}}-{{{{{{{\rm{RD}}}}}}}}_{{{{{{{\rm{null}}}}}}}}}{{{{{{{{\rm{RD}}}}}}}}_{{{{{{{\rm{pref}}}}}}}}+{{{{{\rm{RD}}}}}}_{{{{{{{\rm{null}}}}}}}}}$$

9 $${{{{{{\rm{OSI}}}}}}}=\frac{{{{{{{{\rm{RO}}}}}}}}_{{{{{{{\rm{pref}}}}}}}}-{{{{{{{\rm{RO}}}}}}}}_{{{{{{{\rm{orth}}}}}}}}}{{{{{{{{\rm{RO}}}}}}}}_{{{{{{{\rm{pref}}}}}}}}+{{{{{{{\rm{RO}}}}}}}}_{{{{{{{\rm{orth}}}}}}}}}$$

where ${{{{{{{\rm{RD}}}}}}}}_{{{{{{{\rm{pref}}}}}}}}$ and ${{{{{{{\rm{RD}}}}}}}}_{{{{{{{\rm{null}}}}}}}}$ are the responses to the preferred and null direction (180-deg from the preferred direction), ${{{{{{{\rm{RO}}}}}}}}_{{{{{{{\rm{pref}}}}}}}}$ and ${{{{{{{\rm{RO}}}}}}}}_{{{{{{{\rm{orth}}}}}}}}$ are the responses to the preferred and orthogonal orientation (90-deg from the preferred orientation).

#### Moving-dots size tuning

To characterize the neuronal size preference, the dot size tuning curves were measured based on the evoked responses to moving dots within the 100–1000 ms window after the onset of dot movements. The spontaneous firing rate calculated within the 0–100 ms window before the presentation of dots was subtracted. The moving-dots evoked responses were then fitted with a ratio of Gaussians (RoG) model as follows:

10 $${{{{{{\rm{Res}}}}}}}(x)=\frac{{{{{{{\rm{a}}}}}}}_{c}{{{{{{\rm{G}}}}}}}_{c}({{{{{\rm{x}}}}}})}{1+{{{{{{\rm{a}}}}}}}_{s}{{{{{{\rm{G}}}}}}}_{s}({{{{{\rm{x}}}}}})}$$

11 $${{{{{{\rm{G}}}}}}}_{c}({{{{{\rm{x}}}}}})={\left(\frac{2}{\sqrt{\pi }}{\int }_{0}^{x}{e}^{-{(\frac{y}{{w}_{c}})}^{2}}{dy}\right)}^{2}$$

12 $${{{{{{\rm{G}}}}}}}_{s}({{{{{\rm{x}}}}}})={\left(\frac{2}{\sqrt{\pi }}{\int }_{0}^{x}{e}^{-{(\frac{y}{{w}_{s}})}^{2}}{dy}\right)}^{2}$$

where *x* is the dot size, ${{{{{{\rm{a}}}}}}}_{c}$, ${{{{{{\rm{w}}}}}}}_{c}$, ${{{{{{\rm{a}}}}}}}_{s}$ and ${{{{{{\rm{w}}}}}}}_{s}$ are the amplitudes and widths of center and surround Gaussians. We constrained ${{{{{{\rm{a}}}}}}}_{c}$, ${{{{{{\rm{a}}}}}}}_{s}$ > 0 and ${{{{{{\rm{w}}}}}}}_{c}$ < ${{{{{{\rm{w}}}}}}}_{s}$.

Size tuning width was defined by the difference of the dot size between the first half of the peak response and the peak response as follows:

13 $${{{{{{\rm{Size}}}}}}}\,{{{{{{\rm{tuning}}}}}}}\,{{{{{{\rm{width}}}}}}}={{{{{{\rm{size}}}}}}}\left({{{{{{\rm{PeakRes}}}}}}}\right)-{{{{{{\rm{size}}}}}}}({{{{{{\rm{PeakRes}}}}}}}/2)$$

where ${{{{{{\rm{size}}}}}}}\left({{{{{{\rm{PeakRes}}}}}}}\right)$ is the dot size which evokes the maximum visual response and ${{{{{{\rm{size}}}}}}}({{{{{{\rm{PeakRes}}}}}}}/2)$ is the minimum dot size which evokes half of the maximum response.

#### Visually evoked responses

To compare visually evoked responses of SC neurons at different depths, we measured the normalized firing rate (${{{{{{\rm{Norm}}}}}}}.{{{{{{\rm{FR}}}}}}}$) as follows:

14 $${{{{{{\rm{Norm}}}}}}}.{{{{{{\rm{FR}}}}}}}=\frac{{R}_{{{{{{{\rm{mean}}}}}}}}-{R}_{{{{{{{\rm{spont}}}}}}}.}}{{R}_{{{{{{{\rm{spont}}}}}}}.}}$$

where ${R}_{{{{{{{\rm{mean}}}}}}}}$ and ${R}_{{{{{{{\rm{spont}}}}}}}.}$ are the average evoked and spontaneous firing rates, respectively.

The fold change was calculated to evaluate the response change caused by optogenetic manipulation as follows:

15 $${{{{{{\rm{Fold}}}}}}}\,{{{{{{\rm{Change}}}}}}}=\frac{{{{{{{{\rm{Norm}}}}}}}.{{{{{{\rm{FR}}}}}}}}_{{{{{{{\rm{LED}}}}}}}\, {{{{{{\rm{OFF}}}}}}}}-{{{{{{{\rm{Norm}}}}}}}.{{{{{{\rm{FR}}}}}}}}_{{{{{{{\rm{LED}}}}}}}\, {{{{{{\rm{ON}}}}}}}}}{{{{{{{{\rm{Norm}}}}}}}.{{{{{{\rm{FR}}}}}}}}_{{{{{{{\rm{LED}}}}}}}\, {{{{{{\rm{OFF}}}}}}}}}$$

where ${{{{{{{\rm{Norm}}}}}}}.{{{{{{\rm{FR}}}}}}}}_{{{{{{{\rm{LED}}}}}}\; {{{{{\rm{OFF}}}}}}}}$ and ${{{{{{{\rm{Norm}}}}}}}.{{{{{{\rm{FR}}}}}}}}_{{{{{{{\rm{LED}}}}}}\; {{{{{\rm{ON}}}}}}}}$ are the normalized visual responses of SC neurons with and without LED, respectively.

#### Surround suppression index

Following previous studies^59^, the strength of inhibition from the surrounding area of the RF was quantified by the surround suppression index (SSI) as follows:

16 $${{{{{{\rm{SSI}}}}}}}=\frac{{R}_{{{{{{{\rm{pref}}}}}}}}-{R}_{{{\mbox{large}}}-{{\mbox{siz}}}e}}{{R}_{{{{{{{\rm{pref}}}}}}}}}$$

where ${R}_{{{{{{{\rm{pref}}}}}}}}$ is the response to the dots of the optimal size and ${R}_{{{\mbox{large}}}-{{\mbox{siz}}}e}$ is the response to the dots of largest size tested (10°–20°).

#### Moving-dots detection tuning

In the visually guided approaching behavioral tests, the success rate was calculated for each dot size and then fitted into a Sigmoid function as follows:

17 $$P(D)=\frac{{{{{{\rm{a}}}}}}}{1+{e}^{-b*D}}+c$$

where *P* is the task performance to the moving dot of size *D*. To quantify the perceptual moving-dot size, dot size at half of the best success rate (${D}_{50}$) was defined as follows:

18 $$({P{DS}}_{50})=\frac{{P}_{\max }-{P}_{\min }}{2}$$

where ${P}_{\max }$ and ${P}_{s}$ are the maximum and minimum task performance. *P* is the performance to the moving dot of size ${{DS}}_{50}$.

#### Current source density analysis

To identify the recording depth in SC, we performed current source density (CSD) analysis^40,45^. The LFP signal was extracted from the raw signal by a low-pass filter (150 Hz cutoff). The discrete second derivative across the electrode sites was computed from the average LFP and smoothed with a Gaussian kernel. The CSD map shows a spatially series of sinks and sources of current during visual stimulation. The inflection point between the first current source and sink was used to mark the boundary between the superficial gray layer and optic layer of SC.

### Statistics

The sample size of recording units was determined by a prior power. Otherwise, sample sizes were selected based on our previous experience or related research. For animals with multiple assays, the sequence of assays was randomized. Investigators were not blind to group allocation or data collection, but the analyses were performed blind to the conditions of experiments. The statistical analysis was performed using Prism version 9 (GraphPad). The Chi square was used to determine whether there is a statistically significant difference in more than two conditions (dot contrast or size). The Fisher exact test was used to compare the behavioral performance from the same group of animals. Statistical comparison depends on the normality of data. The Kolmogorov–Smirnov normality test was used to test for normality. For one or two unpaired groups of non-normally distributed data, we used the Mann–Whitney test to determine the significance. Wilcoxon matched-pairs signed rank test was used for paired groups of non-normally distributed data. Kruskal–Wallis test and Dunn’s multiple comparison test were used for multiple groups of non-normally distributed data. For the normally distributed data, one or two samples *t*-tests were used to compare the data from one or two unpaired groups, and paired *t*-tests were used to compare data from the same units or animal. One-way ANOVA and Tukey’s multiple comparisons tests were used in comparisons of multiple groups of normally distributed data. F test was used to compare the variance of two groups. Data were displayed as mean ± s.e.m. if not otherwise mentioned. Box plots depicted the median, upper/lower quartile, and maximum/minimum.

### Reporting summary

Further information on research design is available in the Nature Portfolio Reporting Summary linked to this article.

### Supplementary information

Supplementary Information

Peer Review File

Reporting Summary

### Source data

Source Data

## Data Availability

The data generated in this study are provided in the Supplementary Information/Source Data file. Source data are provided with this paper.

## Code availability

The Python code for animal detection is available at https://github.com/GuangWei-Zhang/TraCon-Toolbox. Customized MATLAB scripts for electrophysiological recordings can be found at https://zenodo.org/records/10019073. Other code used in this study can be obtained from the corresponding authors upon request.
