# Supplementary material for: Enhancement and contextual modulation of visuospatial processing by thalamocollicular projections from ventral lateral geniculate nucleus
Source: Nat Commun. 2023 Nov 10;14:7278. doi: 10.1038/s41467-023-43147-9 (PMC10638288; doi:10.1038/s41467-023-43147-9)
Supplement: Supplementary file 1 — Supplementary Information [file 41467_2023_43147_MOESM1_ESM.pdf]

Supplementary Figures

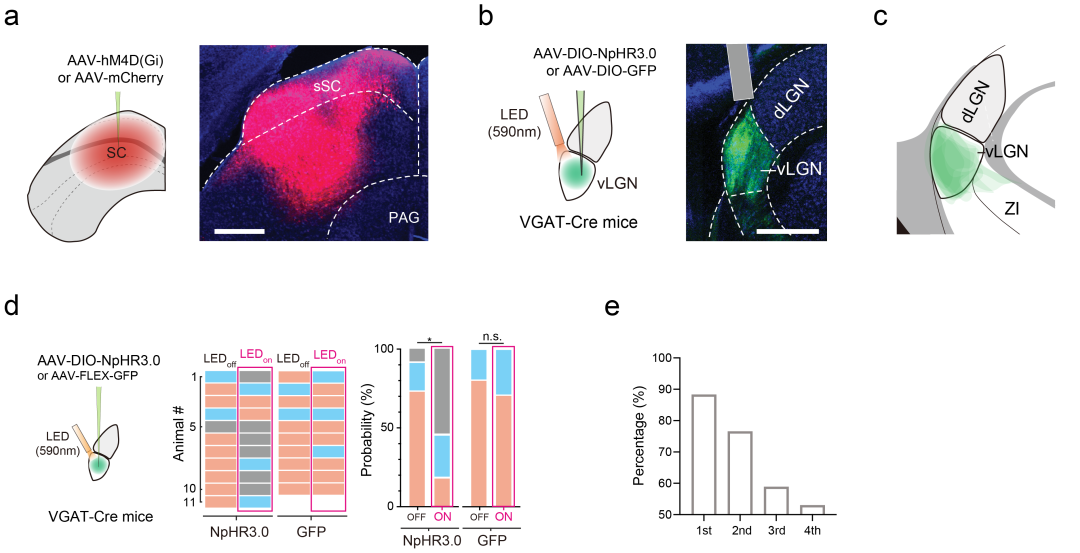

Supplementary Fig. 1. Additional results related to innate approach behavior. **a,** Example image showing the expression of AAV-hSyn-hM4D(Gi) in SC. Scale bar: 500 μm. PAG, the periaqueductal gray; sSC, superficial SC. **b,** Left, schematic of viral injection of AAV-DIO-NpHR3.0 in vLGN of VGAT-Cre mice. Right, example image showing the expression at the injection site. Scale bar: 500 μm. dLGN, the dorsal lateral geniculate nucleus of the thalamus. **c,** Superimposed color-coded viral expression in vLGN of VGAT-Cre mice. n = 11 mice. **d,** Left, schematic of optogenetic silencing of vLGN VGAT+ neurons by injecting AAV-DIO-NpHR3.0 into VGAT-Cre mice. Middle and right, comparison of approaching performance without and with LED illumination. n = 11 and 10 mice for NpHR3.0 and GFP control groups, respectively. *p = 0.03; n.s., not significant, p > 0.99, Fisher exact test. **e,** Probability of correct trials over four consecutive trials with 2-3 min intervals. n = 9 female and 8 male mice. Source data are provided as a Source Data file.

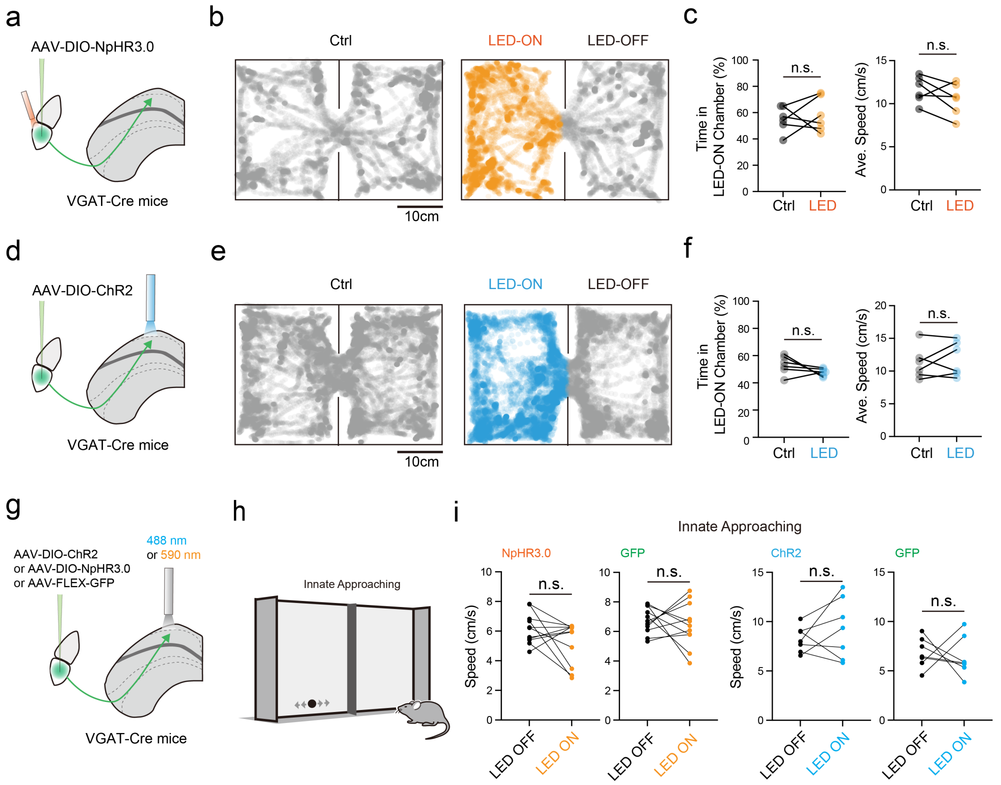

Supplementary Fig. 2. Additional results about minimum emotional effects of optogenetic manipulations. **a,** Schematic of optogenetic silencing of vLGN VGAT+ neurons. **b,** Locomotion tracking for an example mouse in a control (LED-Off) session and a LED-On session in the two-chamber place preference test. LED stimulation was applied (i.e. to silence vLGN neurons) whenever the mouse was in the "LED-On" chamber. **c,** Percentage of time spent (left) and average locomotion speed (right) in the designated "LED-On" chamber in the control condition and LED-On condition. n = 6 mice, n.s., not significant, p = 0.6302 and 0.1866, respectively, two-tailed paired t-test. **d-f,** Similar to **a-c** but for optogenetic activation of vLGN VGAT+ axon terminals in SC. n = 6 mice, p = 0.1749 and 0.4765, respectively, two-tailed paired t-test. **g,** Schematic of optogenetic manipulations of vLGN VGAT+ axons in SC. **h,** Schematic of innate approaching test. **i,** Left, average speed during approaching for NpHR3.0-expressing and GFP control groups. n = 11 and 11 mice, p = 0.1364 and 0.6239, respectively, two-tailed paired t-test. Right, average speed during approaching for ChR2-expressing and GFP control groups. n = 7 and 7 mice, p = 0.3331 and 0.7521, respectively, two-tailed paired t-test. Source data are provided as a Source Data file.

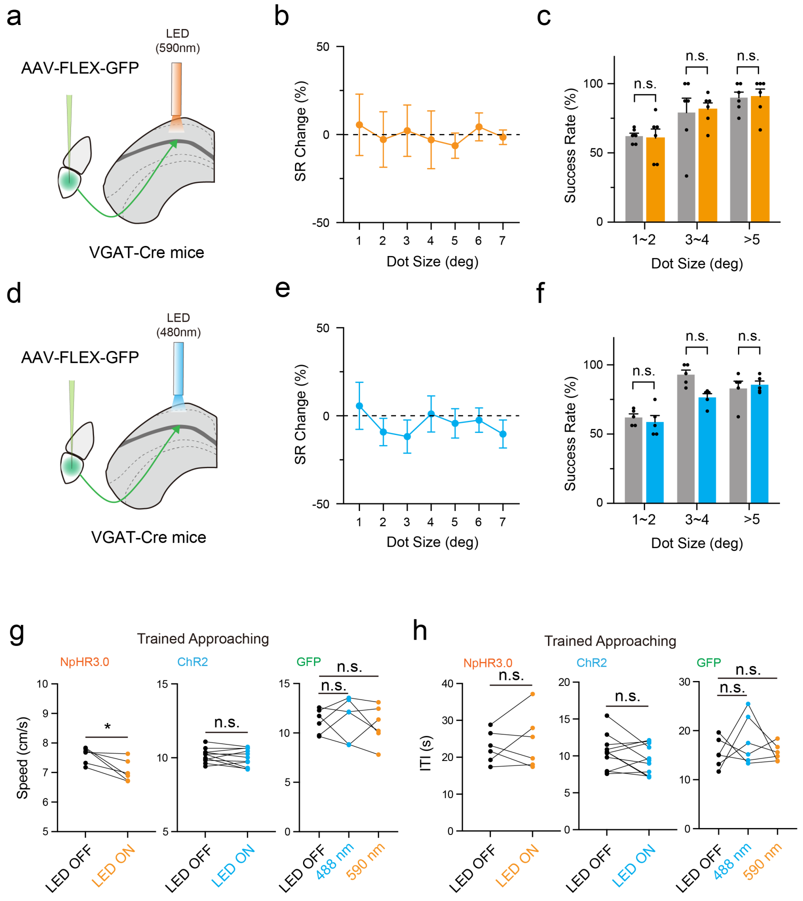

Supplementary Fig. 3. Control experiments for trained approaching behavior. **a,** Schematic of viral injection and amber light application in GFP control mice. **b,** Percentage changes in success rate (SR) at different dot sizes. Data are presented as mean values ± s.d. Bar represents s.d. **c,** Comparison of success rates between LED-Off (gray) and LED-On (orange) conditions for different dot size ranges. n = 36 sessions from 6 mice, n.s., $p > 0.05$, two-tailed paired t-test. Data are presented as mean values ± s.e.m. Error bars, s.e.m. **d-f,** Similar to **a-c** but for blue light application in GFP control mice. n = 30 sessions from 5 mice, n.s., $p > 0.05$, two-tailed paired t-test. **g,** Average speed for approaching without and with LED application for ChR2 (n = 11), NpHR3.0 (n = 6) and GFP (n = 6) mice. p = 0.021, 0.293, 0.931, 0.913, two-tailed paired t-test, one-way ANOVA and Tukey's multiple comparisons tests. **h,** Similar to g but for inter-trial-interval (ITI). p = 0.551, 0.175, 0.674, 0.999, two-tailed paired t-test, one-way ANOVA and Tukey's multiple comparisons tests. Source data are provided as a Source Data file.

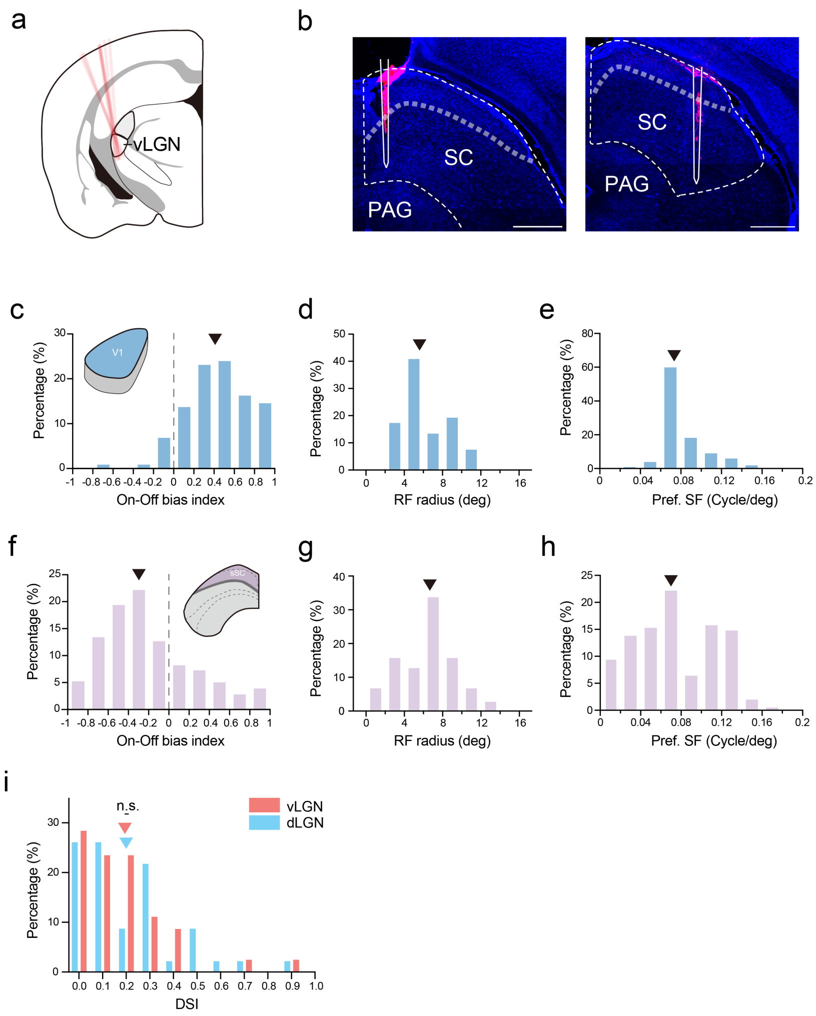

Supplementary Fig. 4. Additional results for visual response properties of V1, SC and LGN neurons. **a,** Tracks of the recording electrodes (red) in vLGN recording experiments. **b,** Example tracks for medial (left) and lateral (right) SC recording. Scale bar: 500 µm. **c,** Distribution of ON-OFF bias indices of V1 neurons. n = 117 units from 3 mice. **d,** Distribution of receptive field radiuses of V1 neurons. Arrowhead marks the median value. n = 51 units. **e,** Distribution of preferred spatial frequencies (Pref. SF) of V1 neurons. Arrowhead marks the median value. n = 99 units. **f-h,** Similar to **c-e** but for SC neurons. ON-OFF bias index, n = 537 units; RF radius, n = 96 units; Pref. SF, n = 204 units. **i,** Comparison of direction selectivity index (DSI) between dLGN (blue) and vLGN (red) neurons. dLGN vs. vLGN, n = 46, 81, p = 0.3262, two-tailed two-sample t-test. Arrowheads indicate the median values. Source data are provided as a Source Data file.

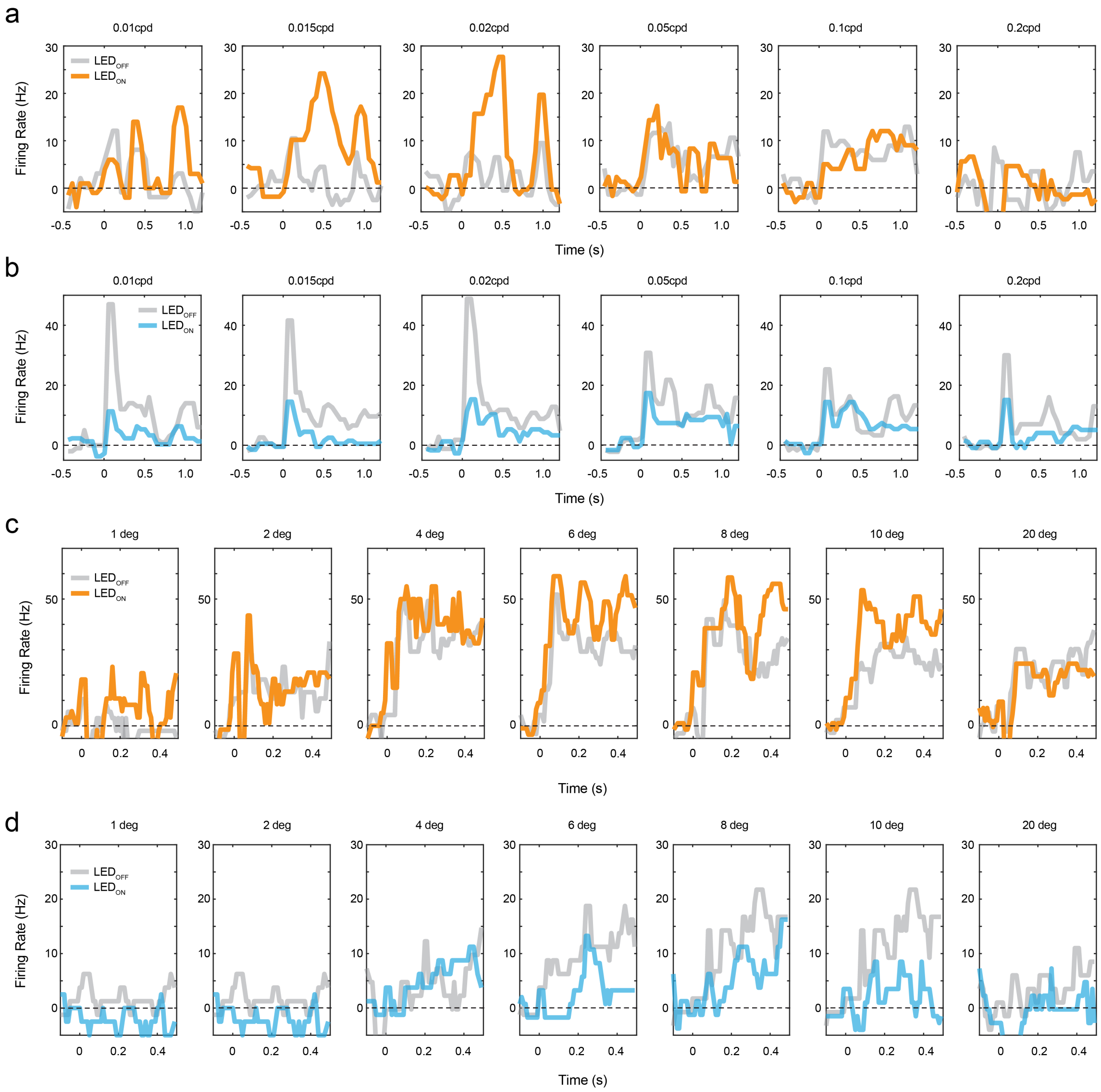

Supplementary Fig. 5. Changes of firing rates by optogenetic manipulations. a, PSTHs for spike responses without (gray) and with (orange) optogenetic silencing of vLGN neurons at different SFs, for the neuron shown in Fig. 5b. **b,** PSTHs for spike responses without (gray) and with (blue) optogenetic activation of vLGN-SC axons at different SFs, for the neuron shown in Fig. 5g. **c,** PSTHs for spike responses without (gray) and with (orange) optogenetic silencing of vLGN neurons at different dot sizes, for the neuron shown in Fig. 5d. **d,** PSTHs for spike responses without (gray) and with (blue) optogenetic activation of vLGN-SC axons at different dot sizes, for the neuron shown in Fig. 5i. Source data are provided as a Source Data file.

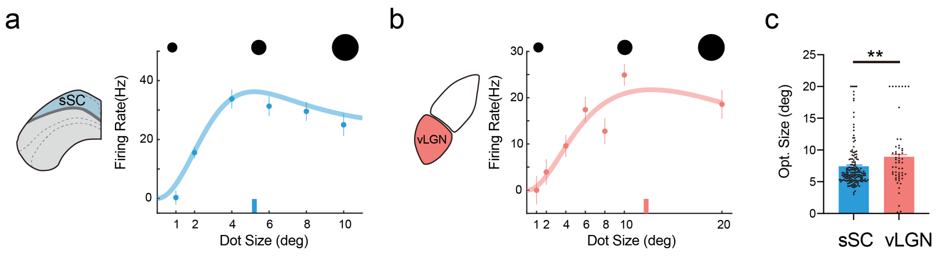

Supplementary Fig. 6. Size tuning of vLGN and dLGN neurons. a-b, Size tuning curves of two example neurons in SC (a) and vLGN (b). Vertical bars indicate the optimal size. **c,** Comparison of the optimal size between SC and vLGN. n = 167 and 54 neurons, respectively, **p = 0.0047, two-tailed Mann-Whitney test. Source data are provided as a Source Data file.

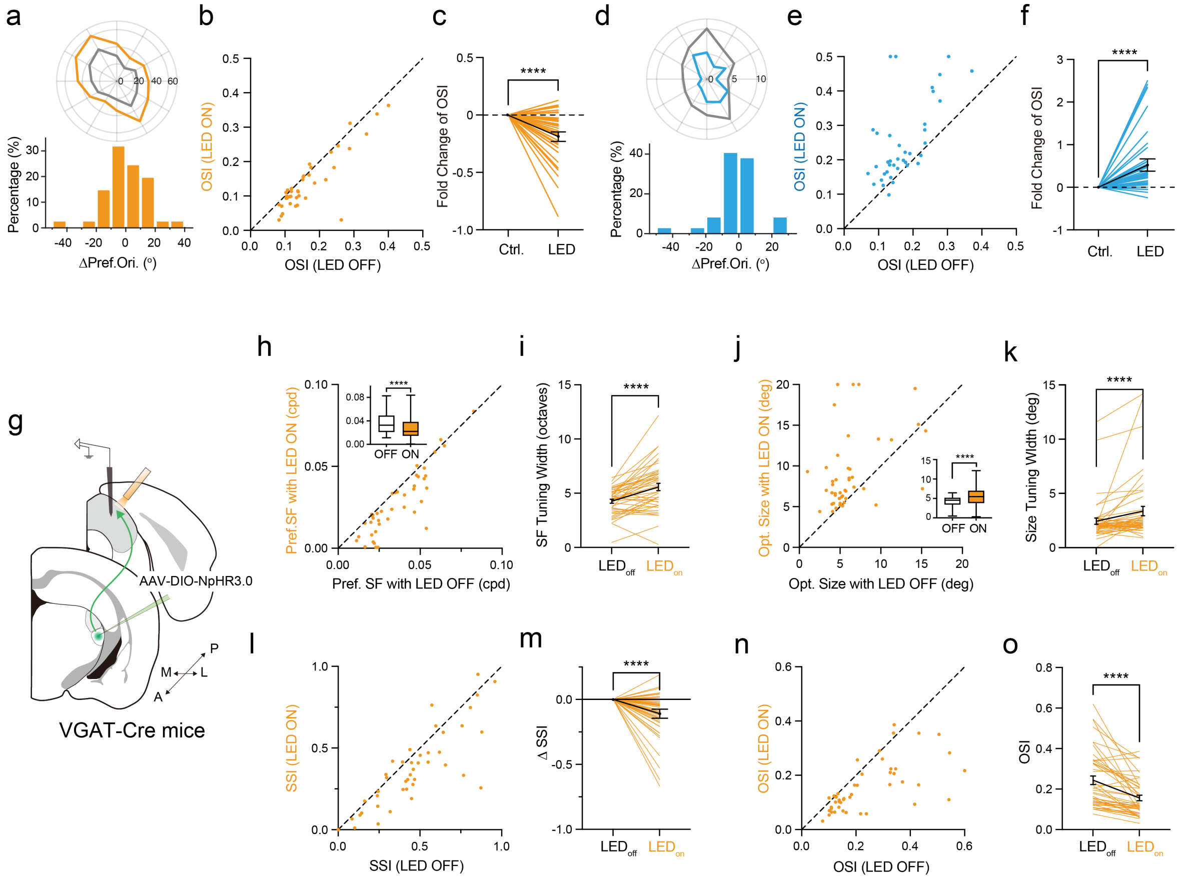

Supplementary Fig. 7. Modulation of SC orientation tuning and effects of silencing vLGN-SC axons. **a,** Top, polar plot of orientation tuning for an example neuron without (gray) and with (orange) silencing vLGN VGAT+ neurons. Bottom, distribution of changes in the preferred orientation (Pref. Ori.) of SC neurons by silencing vLGN neurons. **b,** Comparison of orientation selectivity index (OSI) of SC neurons with vs. without silencing of vLGN neurons. **c,** Fold change of OSI. n = 41 neurons, ****p < 0.0001, two-tailed Wilcoxon matched-pairs signed rank test. **d-f,** Similar to a-c but for activation of vLGN-SC axons. n = 37 neurons, ****p < 0.0001, two-tailed Wilcoxon matched-pairs signed rank test. **g,** Schematic of viral injection and silencing of vLGN-SC axons. **h,** Preferred orientation (Pref. Ori.) of SC neurons with vs. without silencing vLGN-SC axons. Dashed line is the unity line. Inset, mean Pref. Ori. in LED-Off and LED-On conditions. n = 43 neurons, ****p < 0.0001, two-tailed Wilcoxon matched-pairs signed rank test. **i,** Comparison of SF tuning bandwidths in LED-Off and LED-On conditions. Black symbol represents mean ± s.e.m. n = 43 neurons, ****p < 0.0001, two-tailed paired t-test. **j-k,** Similar to **h-i** but for preferred size and size tuning bandwidth. n = 46 neurons, ****p < 0.0001, two-tailed Wilcoxon matched-pairs signed rank test. **l,** Comparison of surround suppression indices (SSIs) in LED-On and LED-Off conditions. **m,** Changes in SSI. n = 46 neurons, ****p < 0.0001, two-tailed Wilcoxon matched-pairs signed rank test. **n-o,** Comparison of OSIs in LED-On and LED-Off conditions. n = 45 neurons, ****p < 0.0001, two-tailed Wilcoxon matched-pairs signed rank test. **h-j**, Center lines indicate the median, limits indicate the upper/lower quartiles and whiskers represent the minimum/maximum. Source data are provided as a Source Data file.

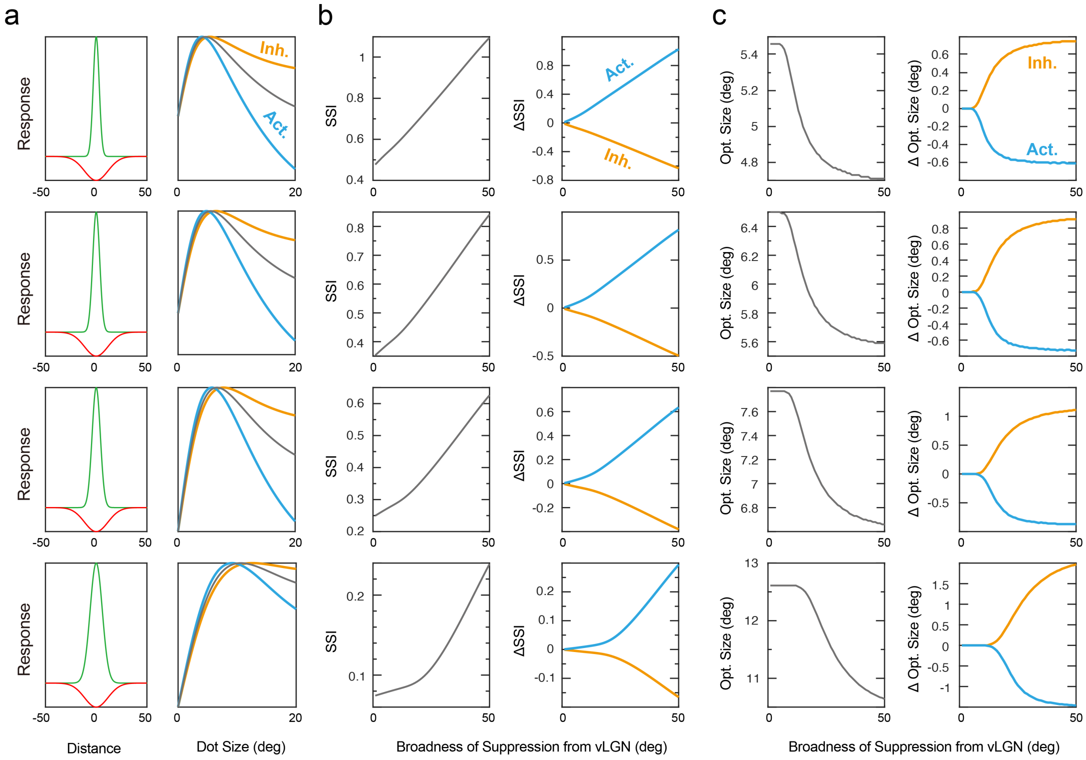

Supplementary Fig. 8. Modeling of size tuning at different excitatory spatial profiles. **a,** Left, spatial profiles of excitatory (green) and inhibitory (red) inputs to a SC neuron. Right, size tuning in the control condition (gray) and after activation (blue) or suppression (orange) of vLGN input. **b,** SSI (left) and changes in SSI by optogenetic manipulations (right) at different spatial broadnesses of vLGN input. **c,** Optimal size (left) and changes in optimal size by optogenetic manipulations (right) at different spatial broadnesses of vLGN input. For each row, the excitatory input profile is fixed as depicted by the green curve in (a). Source data are provided as a Source Data file.

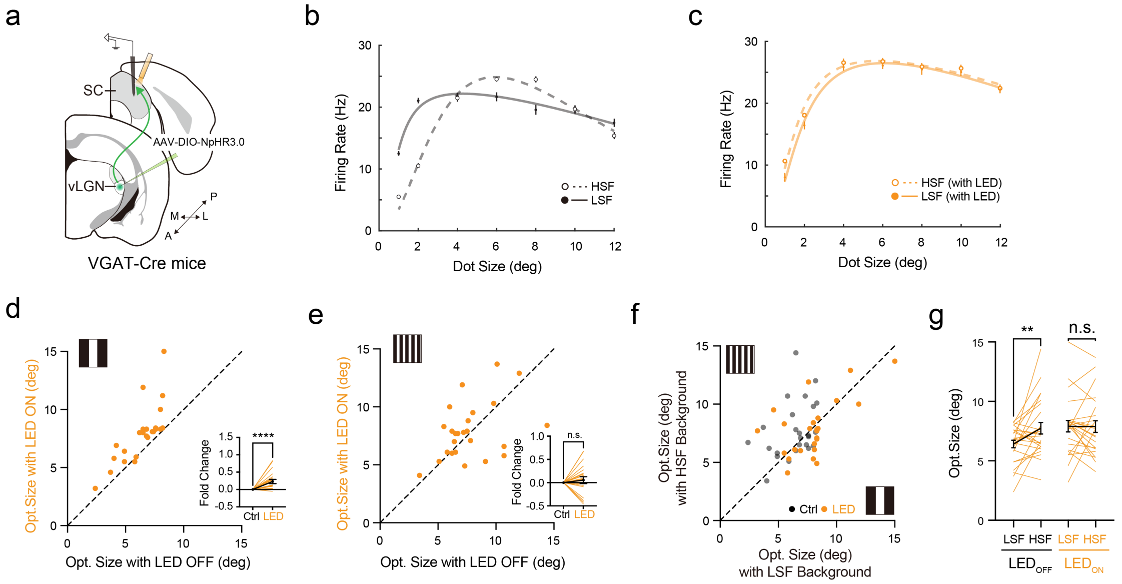

Supplementary Fig. 9. The vLGN-SC projection modulates SC optimal size in a context-dependent manner. **a,** Schematic of silencing vLGN-SC axons and recording in SC. **b-c,** Size tuning of an example SC neuron under low- (solid line and black dots) or high-SF (dashed line and hollow dots) background in the control condition (b) and when silencing vLGN-SC axons (c). Data are presented as mean values ± s.e.m. **d,** Optimal size in the LED-On vs. LED-Off condition under low-SF background. Inset, fold change of optimal size. n = 25 neurons, ****p < 0.0001, two-tailed Wilcoxon matched-pairs signed rank test. Bar represents s.e.m. **e,** Optimal size in the LED-On vs. LED-Off condition under high-SF background. n = 25 neurons, p = 0.300, two-tailed paired t-test. **f,** Optimal size with high-SF (HSF) vs. low-SF (LSF) background in the LED-Off (gray) or LED-On (orange) condition. **g,** Comparison of optimal sizes between HSF and LSF backgrounds in LED-Off and LED-On conditions. n = 25 neurons, **p = 0.009; n.s., not significant, p = 0.956, two-tailed paired t-test. Black symbol represents mean ± s.e.m. Source data are provided as a Source Data file.
