# Supplementary material for: Enhancement and contextual modulation of visuospatial processing by thalamocollicular projections from ventral lateral geniculate nucleus
Source: Nat Commun. 2023 Nov 10;14:7278. doi: 10.1038/s41467-023-43147-9 (PMC10638288; doi:10.1038/s41467-023-43147-9)
Supplement: Supplementary file 3 — Reporting Summary [file 41467_2023_43147_MOESM3_ESM.pdf]

nature portfolio

Corresponding author(s): Huizhong W. Tao

Last updated by author(s): 10/20/2023

Reporting Summary

Nature Portfolio wishes to improve the reproducibility of the work that we publish. This form provides structure for consistency and transparency in reporting. For further information on Nature Portfolio policies, see our Editorial Policies and the Editorial Policy Checklist.

Statistics

For all statistical analyses, confirm that the following items are present in the figure legend, table legend, main text, or Methods section.

n/a	Confirmed	
☐	☒	The exact sample size (*n*) for each experimental group/condition, given as a discrete number and unit of measurement
☐	☒	A statement on whether measurements were taken from distinct samples or whether the same sample was measured repeatedly
☐	☒	The statistical test(s) used AND whether they are one- or two-sided<br>*Only common tests should be described solely by name; describe more complex techniques in the Methods section.*
☐	☒	A description of all covariates tested
☐	☒	A description of any assumptions or corrections, such as tests of normality and adjustment for multiple comparisons
☐	☒	A full description of the statistical parameters including central tendency (e.g. means) or other basic estimates (e.g. regression coefficient) AND variation (e.g. standard deviation) or associated estimates of uncertainty (e.g. confidence intervals)
☐	☒	For null hypothesis testing, the test statistic (e.g. *F*, *t*, *r*) with confidence intervals, effect sizes, degrees of freedom and *P* value noted<br>*Give P values as exact values whenever suitable.*
☒	☐	For Bayesian analysis, information on the choice of priors and Markov chain Monte Carlo settings
☒	☐	For hierarchical and complex designs, identification of the appropriate level for tests and full reporting of outcomes
☒	☐	Estimates of effect sizes (e.g. Cohen's *d*, Pearson's *r*), indicating how they were calculated

Our web collection on statistics for biologists contains articles on many of the points above.

Software and code

Policy information about availability of computer code

Data collection	We used the Open Ephys acquisition system and a NeuroNexus 64-channel silicone probe to record electrophysiological signals. We used an Olympus confocal microscope (FluoView1000) to acquire imaging data. Behavioral data were recorded with a video camera. We used a customized Python software for real-time animal detection as to trigger optogenetic stimulation.
Data analysis	We used customized Python codes for animal detection and behavioral analysis. Codes are available at https://github.com/ GuangWei-Zhang/ TraCon-Toolbox. For in vivo electrophysiological data, we used Offline Sorter (x64V4, Plexon) for spike sorting. We used customized MATLAB scripts for electrophysiological data analysis, which can be found at https://zenodo.org/records/10019073.. We used ImageJ for image analysis. Statistics were performed using GraphPad (v8.0.2).

For manuscripts utilizing custom algorithms or software that are central to the research but not yet described in published literature, software must be made available to editors and reviewers. We strongly encourage code deposition in a community repository (e.g. GitHub). See the Nature Portfolio guidelines for submitting code & software for further information.

Data

Policy information about availability of data

All manuscripts must include a data availability statement. This statement should provide the following information, where applicable:

- Accession codes, unique identifiers, or web links for publicly available datasets
- A description of any restrictions on data availability
- For clinical datasets or third party data, please ensure that the statement adheres to our policy

Source data for all figures of this study are available from the Source Data file published along the manuscript. Raw data will be available from the corresponding authors upon reasonable requests.

Research involving human participants, their data, or biological material

Policy information about studies with human participants or human data. See also policy information about sex, gender (identity/presentation), and sexual orientation and race, ethnicity and racism.

Reporting on sex and gender	N/A
Reporting on race, ethnicity, or other socially relevant groupings	N/A
Population characteristics	N/A
Recruitment	N/A
Ethics oversight	N/A

Note that full information on the approval of the study protocol must also be provided in the manuscript.

Field-specific reporting

Please select the one below that is the best fit for your research. If you are not sure, read the appropriate sections before making your selection.

☒ Life sciences ☐ Behavioural & social sciences ☐ Ecological, evolutionary & environmental sciences

For a reference copy of the document with all sections, see nature.com/documents/nr-reporting-summary-flat.pdf

Life sciences study design

All studies must disclose on these points even when the disclosure is negative.

Sample size	When it is possible, a prior power analysis was used to determine sample sizes. Otherwise, sample sizes were selected based on previous experience from related research and literature (Hoy JL et al., 2019; Ciftcioglu UM et al., 2020; Salay LD et al., 2021; Fratzl A et al., 2021). Sample size for each experiment is described in the figure legend.
Data exclusions	Data were not included from animals if there was mistargeting of viral injection or misplacement of fiber implantation.
Replication	All results in this study, including behavioral tests, in vivo electrophysiological recording, optogenetic and pharmacological experiments were replicated in multiple cohorts/animals, independently. For anatomical images, experiments were replicated in at least three animals. For electrophysiology, recordings were performed from at least two different animals.
Randomization	Animals were randomly assigned to control and experimental groups, and tested in random order.
Blinding	The experimenters were not blind to the animal allocation to experiments. However, the procedure of behavioral test and data collection were automatically controlled by the computer software and the experimenters were blind to the sequence of visual stimulation. In addition, in analysis of behavioral data the investigators were blind to the conditions of experiments as data obtained under different conditions were pooled together for an automatic batch analysis with computer software.

Reporting for specific materials, systems and methods

We require information from authors about some types of materials, experimental systems and methods used in many studies. Here, indicate whether each material, system or method listed is relevant to your study. If you are not sure if a list item applies to your research, read the appropriate section before selecting a response.

Materials & experimental systems

n/a	Involved in the study	
☒	☐	Antibodies
☒	☐	Eukaryotic cell lines
☒	☐	Palaeontology and archaeology
☐	☒	Animals and other organisms
☒	☐	Clinical data
☒	☐	Dual use research of concern
☒	☐	Plants

Methods

n/a	Involved in the study	
☒	☐	ChIP-seq
☒	☐	Flow cytometry
☒	☐	MRI-based neuroimaging

Animals and other research organisms

Policy information about studies involving animals; ARRIVE guidelines recommended for reporting animal research, and Sex and Gender in Research

Laboratory animals	Male and female wild-type C57BL/6J and transgenic Vgat-IRES-Cre (Jackson Laboratories, RRID: IMSR_JAX:016962), Ai14 (Jackson Laboratories, RRID: MSR_JAX:007914) and Ai75 (Jackson Laboratories, RRID: IMSR_JAX:025106) were used in this study. Male and female adult (8-12 weeks old) mice were randomly assigned to each experiment at about equal numbers. The animals were housed at 18–23℃ with 40–60% humidity in a 12-hr light-dark cycle (6AM-6PM light).
Wild animals	The study did not involve wild animals.
Reporting on sex	Both male and female mice were used in this study. No obvious difference was observed between sexes.
Field-collected samples	The study did not involve sample collected from the field.
Ethics oversight	Animal experiments were conducted in accordance with the guidelines for the care and use of laboratory animals of US National Institutes of Health (NIH), and under protocols approved by Institutional Animal Care and Use Committee at University of Southern California.

Note that full information on the approval of the study protocol must also be provided in the manuscript.
