# Supplementary material for: Enhancement and contextual modulation of visuospatial processing by thalamocollicular projections from ventral lateral geniculate nucleus
Source: Nat Commun. 2023 Nov 10;14:7278. doi: 10.1038/s41467-023-43147-9 (PMC10638288; doi:10.1038/s41467-023-43147-9)
Supplement: Supplementary file 2 — Peer Review File [file 41467_2023_43147_MOESM2_ESM.pdf]

nature portfolio

# Peer Review File

## Enhancement and contextual modulation of visuospatial processing by thalamocollicular projections from ventral lateral geniculate nucleus

**Open Access** This file is licensed under a Creative Commons Attribution 4.0 International License, which permits use, sharing, adaptation, distribution and reproduction in any medium or format, as long as you give appropriate credit to the original author(s) and the source, provide a link to the Creative Commons license, and indicate if changes were made. In the cases where the authors are anonymous, such as is the case for the reports of anonymous peer reviewers, author attribution should be to 'Anonymous Referee' followed by a clear attribution to the source work. The images or other third party material in this file are included in the article's Creative Commons license, unless indicated otherwise in a credit line to the material. If material is not included in the article's Creative Commons license and your intended use is not permitted by statutory regulation or exceeds the permitted use, you will need to obtain permission directly from the copyright holder. To view a copy of this license, visit http://creativecommons.org/licenses/by/4.0/.

REVIEWER COMMENTS

Reviewer #1 (Remarks to the Author):

In this study Li et al., investigate the contribution of vLGN projection to the superior colliculus in the context of visually driven behavior. The authors developed a visual assay based on the animal's curiosity and ability to detect a small moving dot and manipulated SC activity or vLGN neurons. They also performed anatomical tracings and recordings in the vLGN. The authors claim the vLGN processes more refined visual information than previously suggested through projections to the superior colliculus. Though the implication of the vLGN seems clear, the specificity of the thalamocollicular projections is less clear cut. Of concern is the fact that GABAergic neurons in an adjacent region, the zona incerta, have also been implicated in hunting and curiosity like behaviors, which renders the interpretation of vLGN silencing experiments more ambiguous. While the authors selectively activate vLGN terminals in the SC, could a similar approach be used to silence those same terminals (though terminal inactivation presents its own technical challenges and interpretation caveat)? This would disambiguate the possibility that either projection to other brain regions than the SC are responsible for the behavior or ZI projection are responsible (ZI only seems to project to the deeper layers of the SC). The authors also suggest that vLGN inputs affect cells located in the most ventral part of the visual layers of the superior colliculus and exert their action through a surround suppression mechanism.

General comment: please provide absolute values throughout experiments and number of animals. Methods would be greatly improved by being more specific to each experiment.

Specific comments:

Figure 1C: Success rate in sham is below 50%. Does this mean that most of the animals don't go all the way to the screen?

Line 99: Implication of the visual layers of the SC: In "The vLGN plays a critical role in SC-dependent visually guided approaching behavior", the authors manipulate SC activity using a chemogenetic approach which results in an impaired performance in the behavior test. Since most neurons in the SC are inhibitory, is silencing neurons without discrimination actually silencing the SC? What promoter was used? The AAV listed in the method is pAAV-hSyn-DIO-hM4D(Gi)-mCherry (Addgene, 44362), which is Cre dependent. Which driver was used? "Impairing SC activity" instead of silencing would be more appropriate. Furthermore, there is a lack of consistency across silencing methods, with chemogenetic or optogenetic approaches used interchangeably. Please justify.

Line 108: "These results demonstrate that the vLGN plays a critical role in the SC-dependent visually guided approaching behavior." These results demonstrate that impairing the SC or the vLGN results in behavioral defects, but not the link between these two structures.

Line 109: Though the aversive effect of the stimulus was tested, the effect on overall locomotion was not addressed.

Line 116: Injection spans many layers of the SC, which could be involved in other modalities than visual processing

Line 117: Presumably a type, AAVretro-Cre instead of AAVretro-GFP?

Line 128: "Our anatomical data suggest that vLGN neurons, by receiving retinal input, may robustly modulate visually evoked activity in SC, including the lateral part of SC serving the low visual field" This statement is too strong, there is no evidence here that the neurons that project to the SC are retinorecipient.

Figure 2: Maintaining the same dot contrast (0.3) for both vLGN activation and silencing would have made comparison easier.

Line 140: The initiation and duration of LED activation is not described in the main text or method. Does the silencing start when the trial is initiated? Does it stay ON for the whole duration of the trail?

Line 148: In the vLGN to SC terminal activation, could the animal be more aroused or motivated? Was there a GFP control group for this experiment?

Line 153 to 197: please provide absolute numbers throughout the description of vLGN visual response properties to facilitate comparison with previous studies. Mean and SD of pref SF, OSI, RF size and so on.

Figure 4A: Recording were performed in the medial SC while the behavior observed is in the lower part of the visual field.

Figure 4h: left panel, is this the average spike rate?

Figure 4P: Legend stipulates that quantification was made in 2 animals so what do the 5 data points represent? Slices?

Line 228: “We made recordings mostly from the lateral part of SC”. All illustration and electrode track show recordings in the medial part of the SC

Line 229: The SF at which the strongest effect are observed seem to be around 0.01cpd, which correspond to 50 degrees of visual angle. Drifting gratings in the methods are described as square or sinusoidal and presented for 1sec, without indication of the temporal frequency. These parameters would probably result in a full field ON or OFF stimulus. This would suggest an effect on contrast sensitivity rather than spatial sensitivity.

Line 250: Please add numerical values. For example, what was the average RF size change?

Line 265: It seems inappropriate to use V1 spatial tuning parameters as driver of SC visual responses when retinal inputs are known to be the major driver

Line 288: The low SF background is described in method as either 0.01 or 0.1. This variation in parameter could make a significant different since it would result in alternating on/off flashes at 0.01cpd.
Figure 8: A denser labeling from VC to SC is expected with the AAV-Cre. The injection site seems pretty unspecific.

Reviewer #2 (Remarks to the Author):

In this manuscript, the authors introduce a new behavioral paradigm for measuring the innate approach behavior to moving block dots, and the dependence of this behavior on the vLGN. Next, they train mice on this same behavior by water reward. They show that the trained behavior is modulated by activity in the vLGN, and can be modified by activating the gabaergic input from the vLGN to the SC. Next, in head-fixed animals, they show that the vLGN is most responsive to relatively large dots, both light-onset and light-offset, but with a bias towards light onset, and have a low preferred spatial frequency. Modulating vLGN activity or its input to the SC, modulates the size tuning and surround suppression in the SC. They present a model that the input from the vLGN provides surround suppression to the SC. This modulation by the vLGN is dependent on the visual context. Finally, they show that the visual cortex does not provide the major drive for response in the vLGN.

The manuscript contains a nice set of beautiful experiments with an original paradigm connecting circuit and behavior. The results are clear and well-presented. The power of each experiment is

more than sufficient to draw a conclusion for the specific question tested in the experiment. Together they lead to new and interesting insight into the circuitry behind visuomotor behavior.

The authors conclude in the abstract, "via visually-driven long-range feedforward inhibition, vLGN serves to refine and contextually modulate visuospatial processing in SC-mediated visuomotor behaviors". I think this specific conclusion is warranted, but one set of experiments appears to be missing from the manuscript. In Figure 1, the authors show that manipulating vLGN activity changes the innate behavior to enter the compartment with the moving dots. In Figure 2, they show that the connection from the vLGN to SC is important in trained approach behavior. The experiment that the connection from the vLGN to SC is important in the innate behavior shown in Figure 1 appears to be missing. Considering all the other nice experiments that were done, it would be good if this experiment would be included, or explained in the manuscript why this is not possible, or argued in the manuscript to what extent the conclusions drawn from the manipulations done for the behavioral paradigm used in Figure 2 are likely to apply to the paradigm presented in Figure 1.

Another question that I have is about the model. The effect of silencing vLGN (Fig. 2f) or activating the connection of vLGN to SC (Fig. 2j) primarily seems to be an overall change of the success rate, rather than a change in the D50. The model for explaining the effect of vLGN on SC responses, however, appears to primarily show an influence on the optimal size, and not on the maximum response of individual neurons. It predicts a change in the response for large stimuli, but not for optimal or small stimuli, if I understand it correctly. Does the model allow the authors to go one step further and explain how the vLGN influences the behavioral success rate?

Next to these larger questions, I have set of very small comments.

L50: "elucidate mystery of the vLGN" is missing "the"

L93: Was the second trial, shown in Fig 1b, measured in a second session, on another day, or in the same session as the first trial? I assume it was done in a second session, based on the methods, but perhaps this can be added explicitly to the results text or figure caption.

L133: Do the authors have data on how quickly the mice adapted?

L118: "SC-projecting vLGN neurons were most densely distributed in the lateral half of the nucleus" The viral injection in the image was done in the medial SC. Would it be more accurate to say use mSC-projecting vLGN neurons, or was this independent of the SC injection location?

L353: "Extended Fig. 3" should be "Extended Fig. 4"

L352-354 "Also, we found that vLGN neurons were robustly activated by moving dots and tuned to large dot sizes (Extended Fig. 3). These observations indicate that vLGN can encode diverse visual information far beyond illuminance levels" But the moving dots would also change the illuminance levels, so an area purely encoding illuminance levels could respond similarly. Perhaps rephrase or include responses to gratings in the argument.

L828. For reproducing the results, it would be nice to mention the luminance level.

L847-853: Upon first reading, it was not clear to me that this was about the water-reward training paradigm used for Figure 2 and not the paradigm used for Figure 1, which follows later in the description of the behavioral paradigms. This was a bit confusing. Perhaps it can be clarified or re-arranged.

L847-853: How long did the training and test sessions take? How many stimuli were shown? Did it mouse have single training and test session?

L875: "Olmpus" should be Olympus.

L901: What was the extent over which the stimulus moved before reversing?

L908: After the visual stimulation was terminated. When was a new trial started?

L916: “48-hr water restriction”. Was the mouse not given any access to water in this period? If so, can this be added explicitly for clarification. Was there also water restriction during the training period following this first 48-hr restriction period? If so, was water given to the animals that did not make enough successful tests, or was a different procedure followed?

L958: The form p^y is an unusual choice of the non-linearity. Is this indeed correct or was y^p intended?

L963: “We constrained a_c, a_s >0.” I do not see the variables a_c and a_s in the preceding equations. Please check.

L1005: There is a spurious ‵.’ in front of the b in the equation.

Reviewer #3 (Remarks to the Author):

In this study, Li and colleagues investigated the influence of the ventral lateral geniculate nucleus (vLGN) on visual processing in the superior colliculus (SC) of mice. The vLGN is an understudied inhibitory prethalamic area involved associated with non-image-forming vision that has recently come into focus as a brain region that mediates visually evoked defensive behaviours (Salay and Huberman, 2021; Fratzl et al., 2021).

The authors of this study find that manipulating vLGN projections to the SC bidirectionally affects both instinctive and learned visually guided approach behaviour and that this effect on behaviour could be explained by the specific and contextual modulation of visuospatial response properties of SC neurons by vLGN projections.

This study is novel, timely and contributes to our understanding of visual pathways involving the vLGN, which have so far been less studied than the canonical thalamocortical pathway.

The authors have employed a wide array of state-of-the-art techniques, including behavioural manipulation experiments in freely moving mice, anatomical tracing experiments and extracellular electrophysiological recordings in different visual brain areas to study the role of vLGN in visual processing.

Extracellular electrophysiological recordings in both the vLGN and the SC are interesting and while they confirm previous results regarding the visual properties of vLGN neurons and the depth-dependent inhibitory effect of vLGN projections on SC neurons, they go beyond the published literature and convincingly show that vLGN can have a more nuanced and specific influence on SC computations depending on the exact visual stimuli presented.

Specifically, vLGN input modulates the size tuning curves of SC neurons, possibly through a surround inhibition mechanism. The authors also describe a visual contextual modulation of SC responses by the vLGN, however the data shown need some clarification (see below).

The behaviour experiments in Figures 1 and 2 need more work. As detailed below, the authors claim that manipulating vLGN circuits changes the perception of a visual stimulus inducing a difference in a decision-making task. However, in Figure 1, it is so far unclear if execution or perception are affected. Moreover, in Figure 2, effect sizes seem partially inconsistent and it is not clear if they are not partially driven by the choice of a specific data fitting model.

A more thorough discussion regarding the influence of vLGN (and other prethalamic) areas on SC computations could improve the manuscript.

Major

85-115 (Figure 1): This behaviour has three possible outcomes: correct decision, wrong decision, and non-decision (trial aborted). It would be important to show all three outcomes separately, as it is known that SC (and vLGN manipulation) can affect behaviour execution. To conclude on the effect of manipulation on visual discrimination, only completed trials should be compared as it is so far unclear if vLGN/SC manipulation does not just increase trial abortion rate instead of affecting visual stimulus discrimination.
Also, the extended data Figure 1 does not fully address this issue and the conclusion on no "emotional" involvement of vLGN circuits and therefore the necessity of vLGN affecting only visual processing is not very convincing, especially as different groups, in particular Salay and Huberman, 2021 and Fratzl et al., 2021 have recently shown - in comparable settings - that vLGN manipulation has strong behavioural effects (e.g., in the plus maze and open maze). The authors mention the difference in axonal distribution of vLGN->SC projections compared to previous studies, and suggest that these could be explained by the previous works having inadvertently targeted ZI instead. There are many alternative reasons for why this could be the case. The vLGN consists of many different types of inhibitory neurons, so slight differences in injection site could explain differences in projection target. Further, different viruses used could have different tropism for different cell types. The injections shown in Fratzl et al, 2021, for example, look as localised as the ones the authors present here. If the authors wish to make this claim publicly, a thorough anatomical rendering of their injections (along the AP axis of the vLGN) would be necessary to corroborate this, as well as a comparative injection of the rostral ZI instead. The above given reasons may be similarly likely reason than ZI 'contamination', which exists also in this study. (see also comments below).

L144-147 Figure 2: It's not clear why the authors performed an inhibition experiment of vLGN somata to show the decrease in success rate, and compare this to the increase of performance by specifically stimulating axons in the SC (which could well activate vLGN neurons through backpropagating action potentials). The interpretation of data from Figure 2F is misleading. While vLGN inhibition indeed appears to consistently reduce correct performance of the task, there does not seem to be any convincing size dependency. When ignoring the first data point of 1 deg where animals perform at chance level (50%) and which is likely below detection threshold (1 deg), it becomes apparent the vLGN inhibition mostly just downshifts performance by a more or less constant value (~10%). The fitted 50% thresholds lay in both groups between 1 and 2 and no stimuli have been probed close to these values to confirm that vLGN inhibition induces an actual change in slope besides just downshifting overall performance. Without additional datapoints close to the thresholds (1.2,1.4,1.6, 1.8 deg for example) it is unclear if the threshold change is real or just an artifact of the fitting method. P-values just below 0.05 could easily change when a single point from a single animal was different at 5 deg for example. Similarly for Figure 2G it might be easier to see the raw (average trace) per mouse instead of the fit, which might be not particularly robust given that most of the change appears to happen within 2 points (1 and 2 deg). Also, surprising that the effect in 2H is much stronger than in 2F (even more apparent when comparing 2L to 2J). This points to some lack of robustness of the fitting model due to lack of data (at critical points). Overall, Figure2J is more convincing, given that the ceiling effect is avoided to the lower contrast stimulus. However, the fits on single animals (Figure 2L) still don't look convincing and the large difference in DS50% between single animals and average fit concerning. Given all those experiments were performed in a paired setting (within animal controls), it might be easier to look at the difference in success rate for each animal at each stimulus size and identify for which stimulus size the difference is the largest.

Figure 3: The analysis of visual response properties is well executed, and is in line with the results of already published work by Ciftcioglu et., 2020 and others. The authors further find that the proportion of OnOff cells is larger than previous reported. They could discuss what the reasons for this could be (again, vLGN is a vastly heterogenous brain region with many different cell types, so sth as simple as the penetration angle could in theory make a huge difference).

Figure 7C, F-H: The dots in none of these plots appear consistent. For example, in 7F (low SF LED on) there are 7 points at exactly 20 deg, while in 7H (low SF LED on, same condition in principle) there are 0. None of the groups seem to match their equivalent group in a different panel. Are

these different neurons, was the data analysed differently or were different experiments performed for each panel? Also the average values seem significantly different (in the example mentioned around 10-12 degs in 7F vs 7.5 in 7G,H). Please clarify.

Minor

L85-115 (Figure 1): It is unclear why SC inhibition is compared to vLGN inhibition (and not vLGN excitation) given that vLGN is mostly inhibitory and vLGN activation decreases SC activity (as shown later in the paper and in Fratzl et al., 2021). Particularly surprising given that in extended data figure 1 both vLGN stimulation and inhibition experiments are shown. Does vLGN stimulation not induce a consistent behavioural effect in the approach experiment? Is a bidirectional effect expected (inhibition or excitation) or is vLGN manipulation just expected to disrupt visual discrimination.

L117: AAVretro-GFP? Should be AAVretro-Cre?

L123-129: The authors mention that GABAergic projections from the vLGN predominantly terminate in the most superficial SC layers, which seems at least partially different from what Salay and Huberman, 2021 and Fratzl et al., 2021 have shown regarding additional projections to the intermediate SC layers. Please discuss possible reasons, see also above.

The authors claim that this discrepancy is most likely due to a more prominent contamination of zona incerta (ZI) neurons in the former mentioned papers. While this remains a valid hypothesis, Fratzl et al., 2021 proposed (supplemental figures) that the most superficial projections could arise from intergeniculate nucleus (IGL) neurons (not mentioned here at all). Also, in the present Figure 1K, projections in deeper SC layers and PAG can still be seen, even if the fluorescence appears less bright than in sSC.
Moreover, throughout the manuscript there seems to be some variability in the shown projection patterns (possibly due to normal variability in the extent of contamination in ZI and IGL), for example in Figure 4B many more axons in the deeper layers can be seen (exactly at the recording site of the probe, consistent with the pattern described by Salay and Huberman, 2021 and Fratzl et al., 2021). Similarly, in Figures 4M-O, the authors show that most SC neurons receiving direct input from vLGN are located significantly deeper than the shown axonal projections in Figure 1K. While extensive apical dendrites (line 222) are possible, the authors should also acknowledge the possibility of direct axonal vLGN projections to the intermediate SC layers, which could also explain the strongest effect of vLGN manipulation on intermediate SC circuits (Figure 4P).

This is also corroborated by Figures 8D-G, where authors show that vLGN neurons receiving V1 input (which likely represent the majority of vLGN neurons according to Govindaiah et al., 2023 and Sumitomo et al.,1979) project significantly deeper than what is shown in Figure 1K.

Finally, it is also possible that some axons in the sSC are labelled due to the retrograde transport of AAV1 (for example Zingg et al., 2017 Figure S1) labelling directly retinal ganglion cells which are known to project extensively in the most superficial SC layer (including VGAT+ RGCs (Cai et al., 2021)). This could be ruled out by retinal histology.

Given the yet not fully cohesive picture of how vLGN and the other caudal prethalamic nuclei IGL and ZI (Fratzl and Hofer, 2022) innervate SC circuits, it could be considered adding a specific section in the discussion of this manuscript.
L139: VGT+ ->VGAT+

L206-210 (Figure 4): Worth mentioning that the result that the effect of vLGN stimulation on SC visual responses being strongest in intermediate layers is not novel but as been shown by Fratzl et al., 2021 (Figure 4), where they performed a nearly identical experiment.

L216-217: It is not novel that SC neurons in deeper layers have larger receptive fields. It has been shown several times before (for example Lee et al., 2020 Figure 3).

L228 (Figure 4 and 5): The authors mention that they mostly record in the lateral SC, however,

the histology shown Figure 4B is quite medial, at most central SC, but not lateral.

Figure 5: Results presented in Figure 5 are very interesting and convincing. The size dependent effect of vLGN manipulation might even be more convincing if in additional panels the spike difference between laser and no laser could be shown (for example in supplemental) dependent on the size of the stimulus (more or less the difference between the traces in Figure 5B, 5D etc.).
Figure 6A: The schematic for the SSI is useful, however, it would be good to add to which actual SSI value this corresponds (presumably around 0.5?)

Figures 6C-6E: Not clear if the fold change is more meaningful than a simple SSI difference, given that small baseline values will increase to uninterpretable large values.

Figure 7: Maybe helpful to add a plot similar to 7B for the LED ON conditions.

Figures 8H-L: Please add to panel what experiment was recorded in cortex vs which ones in vLGN.

L307-310: Unclear how neurons labelled in SC after retroAAV injection in vLGN confirm the vLGN anterograde tracing results. This is the opposite experiment, and regardless it is known that SC projects back to vLGN.

L313-315: See comment regarding vLGN projections to intermediate SC layers.

L318-322: Please comment on how this result is consistent with Govindaiah et al., 2023, who found driver-like input from cortex to vLGN.

L414-419: This model has been proposed before, for example by Fratzl and Hofer, 2022 (their Figure 6B-C is very similar to the here presented Figure 8M). The authors should at least cite to acknowledge.

**Response to Reviewer #1**:

*In this study Li et al., investigate the contribution of vLGN projection to the superior colliculus in the context of visually driven behavior. The authors developed a visual assay based on the animal's curiosity and ability to detect a small moving dot and manipulated SC activity or vLGN neurons. They also performed anatomical tracings and recordings in the vLGN. The authors claim the vLGN processes more refined visual information than previously suggested through projections to the superior colliculus. Though the implication of the vLGN seems clear, the specificity of the thalamocollicular projections is less clear cut. Of concern is the fact that GABAergic neurons in an adjacent region, the zona incerta, have also been implicated in hunting and curiosity like behaviors, which renders the interpretation of vLGN silencing experiments more ambiguous. While the authors selectively activate vLGN terminals in the SC, could a similar approach be used to silence those same terminals (though* ***terminal inactivation*** *presents its own technical challenges and interpretation caveat)? This would disambiguate the possibility that either projection to other brain regions than the SC are responsible for the behavior or ZI projection are responsible (ZI only seems to project to the deeper layers of the SC). The authors also suggest that vLGN inputs affect cells located in the most ventral part of the visual layers of the superior colliculus and exert their action through a surround suppression mechanism.*

*General comment: please provide absolute values throughout experiments and number of animals. Methods would be greatly improved by being more specific to each experiment.*

We thank the reviewer for all the advices. We agree with the reviewer on the importance of establishing the specificity of the functional role of the vLGN-SC projection. To this end, we have performed additional experiments of optogenetically inhibiting the axon terminals from vLGN VGAT+ neurons in SC. In general, effects similar to the cell-body silencing were observed, including those on behavioral performance (**Fig.1g**, **Fig.2e-i**), SF tuning, size tuning, surround suppression, orientation tuning (**Extended Data Fig.7g-o**) and context dependency (**Extended Data Fig.9**). As suggested by the reviewer, we have added all the necessary values in the Results section, and more details for each specific experiment in the Methods.

*Specific comments*:

*Figure 1C: Success rate in sham is below 50%. Does this mean that most of the animals don't go all the way to the screen?*

In the revised **Fig. 1a,b**, we have separately labeled three outcomes: 1) "Correct trial", approaching the screen of visual stimulus (mocked, zero-sized dot for the sham group); 2) "Incorrect trial", approaching the wrong screen; 3) "No-choice", staying out of either end zone within the test window. In the sham group, no choice was made in a high percentage of trials (43%, **Fig. 1b**). In trials with choices made, animal randomly approached either screen. We have clarified this point in the text (page 5, line 95).

*Line 99: Implication of the visual layers of the SC: In "The vLGN plays a critical role in SC-dependent visually guided approaching behavior", the authors manipulate SC activity using a chemogenetic approach which results in an impaired performance in the behavior test. Since most*

*neurons in the SC are inhibitory, is silencing neurons without discrimination actually silencing the SC? What promoter was used? The AAV listed in the method is pAAV-hSyn-DIO-hM4D(Gi)-mCherry (Addgene, 44362), which is Cre dependent. Which driver was used? "Impairing SC activity" instead of silencing would be more appropriate. Furthermore, there is a lack of consistency across silencing methods, with chemogenetic or optogenetic approaches used interchangeably. Please justify.*

We apologize for the confusion. In the experiment of chemogenetic silencing of SC, we used AAV-hSyn-hM4D(Gi)-mCherry, which is not Cre-dependent. This has been clarified in the text (page 5, line 102). The reviewer has made a good point about the majority of SC neurons being inhibitory (we have now cited the paper by Liu et al., Neuron 2023). We have followed the reviewer's suggestion to change the phrase to "impairing SC activity" or "disruption of SC activity" (page 6, line 104).

As SC is a considerably large and thick structure, to explore its general functional role, we considered chemogenetics as a more effective approach since optogenetics may only affect limited layers. For vLGN terminal manipulations, we then used optogenetics (with optic fiber placed above SC) since we knew that vLGN axons projected most strongly to the superficial layers of SC based on our data.

*Line 108: "These results demonstrate that the vLGN plays a critical role in the SC-dependent visually guided approaching behavior." These results demonstrate that impairing the SC or the vLGN results in behavioral defects, but not the link between these two structures.*

To better establish the link, we performed additional experiments with terminal silencing of vLGN VGAT+ axons in SC. This projection-specific manipulation also reduced the performance of innate approaching behavior, while the same photo-stimulation had no effect in GFP control mice (new **Fig. 1g**).

*Line 109: Though the aversive effect of the stimulus was tested, the effect on overall locomotion was not addressed.*

We analyzed the locomotion speed in the place preference test and did not find a difference between LED ON and control conditions (**Extended Data Fig. 2c,f**, right panel). Similarly, inactivation or activation of vLGN-SC terminals did not change the approaching speed (**Extended Data Fig. 2i**).

*Line 116: Injection spans many layers of the SC, which could be involved in other modalities than visual processing.*

We agree with the reviewer that in the SC injection experiment modalities other than visual function (e.g. motor) could be affected. Here, the only purpose of the experiment is to show that the behavior is SC-dependent, and we have not claimed that the effect was due to impaired visual processing. We have now further clarified this point in the text.

*Line 117: Presumably a typo, AAVretro-Cre instead of AAVretro-GFP?*

Thanks for catching this error. It should be "AAVretro-Cre", and we have made the correction (page 7, line 128).

*Line 128: "Our anatomical data suggest that vLGN neurons, by receiving retinal input, may robustly modulate visually evoked activity in SC, including the lateral part of SC serving the low visual field" This statement is too strong, there is no evidence here that the neurons that project to the SC are retinorecipient.*

We have removed "by receiving retinal input".

*Figure 2: Maintaining the same dot contrast (0.3) for both vLGN activation and silencing would have made comparison easier.*

In our new experiments of silencing vLGN-SC axons, we have used moving dots at 0.3 contrast (new **Fig. 2e-i**).

*Line 140: The initiation and duration of LED activation is not described in the main text or method. Does the silencing start when the trial is initiated? Does it stay ON for the whole duration of the trail?*

The photo-inhibition was started 0.1s before the onset of dot movements. It stayed ON until mouse made the approach (when visual stimulation also stopped), or for the whole duration of the test window. The photo-activation was started at the same time of dot movements. We have now clarified these details in the Methods (page 41, line 1039).

*Line 148: In the vLGN to SC terminal activation, could the animal be more aroused or motivated? Was there a GFP control group for this experiment?*

This is a good question, since excitatory inputs (such as visual and auditory) to SC is expected to enhance arousal or motivated behavior. Here, we found that terminal inactivation of GABAergic vLGN input to SC did not change the approaching speed or the inter-trial-interval (ITI) of the self-initiated task, neither did the terminal activation (**Extended Data Fig. 3g-h**). This suggests that activity of the vLGN-SC projection does not affect arousal or motivation.

We have now added the GFP control group for both terminal inactivation and activation experiments. Basically, in the GFP control mice, the success rate was not significantly affected by blue or amber light (**Extended Data Fig. 3a-f**).

*Line 153 to 197: please provide absolute numbers throughout the description of vLGN visual response properties to facilitate comparison with previous studies. Mean and SD of pref SF, OSI, RF size and so on.*

We have provided all the requested absolute numbers in the Results.

*Figure 4A: Recording were performed in the medial SC while the behavior observed is in the lower part of the visual field.*

We would like to clarify that in Fig.4, both the medial and lateral parts of SC were recorded and we found similar inhibitory effects (page 10, line 216; page 11, line 226). We have included representative images of electrode tracks in medial as well as lateral SC (**Extended Data Fig. 4b**). The recordings in Fig. 5-7 were from the lateral SC. To avoid confusion, the illustrations in Fig.5 have been modified to show a more lateral position of the electrode.

*Figure 4h: left panel, is this the average spike rate?*

It is the peak firing rate normalized by the spontaneous firing rate. This has been clarified in the figure and its legend (page 31, line 739).

*Figure 4P: Legend stipulates that quantification was made in 2 animals so what do the 5 data points represent? Slices?*

Thanks for catching this inconsistency. Yes, 5 sections from 2 animals. This has been corrected (page 32, line 754).

*Line 228: "We made recordings mostly from the lateral part of SC". All illustration and electrode track show recordings in the medial part of the SC.*

The illustrations in Fig. 5 have been modified to show a more lateral position of the electrode to avoid confusion. We have also included a new image to show representative recording track in the lateral SC (**Extended Data Fig. 4b**, right panel).

*Line 229: The SF at which the strongest effect are observed seem to be around 0.01cpd, which correspond to 50 degrees of visual angle. Drifting gratings in the methods are described as square or sinusoidal and presented for 1sec, without indication of the temporal frequency. These parameters would probably result in a full field ON or OFF stimulus. This would suggest an effect on contrast sensitivity rather than spatial sensitivity.*

The visual presentation screen covered 120 deg horizontally and 100 deg vertically, thus there could be $\geqslant$ two cycles of drifting gratings presented at any moment. This is thus different from a full field ON or OFF stimulus. We have added this detail in the Methods (page 37, line 916). In addition, the TF used to measure the Pref. SF was 2 cycles/sec (i.e. two cycles were applied).

*Line 250: Please add numerical values. For example, what was the average RF size change?*

Numerical values have been added. The RF size was increased by 18.0% ± 6.1% when vLGN was suppressed, and decreased by 46.0% ± 6.4% with vLGN-SC axons activated (page 12, line 264).

*Line 265: It seems inappropriate to use V1 spatial tuning parameters as driver of SC visual responses when retinal inputs are known to be the major driver.*

The reviewer has raised a good point. We have varied the excitatory tuning parameters between what have been observed for RGC axons in SC and for V1 neurons (page 13, line 286). Qualitatively, similar effects were generated across the range of excitatory spatial parameters (new **Extended Data Fig. 8**).

*Line 288: The low SF background is described in method as either 0.01 or 0.1. This variation in parameter could make a significant different since it would result in alternating on/off flashes at 0.01cpd.*

As we have explained earlier, there were ≥ two cycles of drifting gratings presented at any moment, which is different from alternating On/Off flashes.

*Figure 8: A denser labeling from VC to SC is expected with the AAV-Cre. The injection site seems pretty unspecific.*

In the previous Fig.8g, in order to show postsynaptically labelled soma more clearly, we had intentionally lowered the intensity of signals.  We have switched to the original high-intensity image to show that there was indeed fairly dense labeling of VC axons in SC (see new **Fig. 8g**, cell body labeling now shown in the inset).

**Response to Reviewer #2**:

*…The manuscript contains a nice set of beautiful experiments with an original paradigm connecting circuit and behavior. The results are clear and well-presented. The power of each experiment is more than sufficient to draw a conclusion for the specific question tested in the experiment. Together they lead to new and interesting insight into the circuitry behind visuomotor behavior.*

We thank the reviewers for all the comments.

*The authors conclude in the abstract, "via visually-driven long-range feedforward inhibition, vLGN serves to refine and contextually modulate visuospatial processing in SC-mediated visuomotor behaviors". I think this specific conclusion is warranted, but one set of experiments appears to be missing from the manuscript. In Figure 1, the authors show that manipulating vLGN activity changes the innate behavior to enter the compartment with the moving dots. In Figure 2, they show that the connection from the vLGN to SC is important in trained approach behavior. The experiment that the connection from the vLGN to SC is important in the innate behavior shown in Figure 1 appears to be missing. Considering all the other nice experiments that were done, it would be good if this experiment would be included, or explained in the manuscript why this is not*

*possible, or argued in the manuscript to what extent the conclusions drawn from the manipulations done for the behavioral paradigm used in Figure 2 are likely to apply to the paradigm presented in Figure 1.*

We agree with the reviewer that it is important to show a role of the vLGN-SC connection in innate approaching behavior. We have now performed additional experiments of silencing vLGN VGAT+ axon terminals in SC. This projection-specific manipulation also reduced the performance of innate approaching behavior (new **Fig. 1g**).

*Another question that I have is about the model. The effect of silencing vLGN (Fig. 2f) or activating the connection of vLGN to SC (Fig. 2j) primarily seems to be an overall change of the success rate, rather than a change in the D50. The model for explaining the effect of vLGN on SC responses, however, appears to primarily show an influence on the optimal size, and not on the maximum response of individual neurons. It predicts a change in the response for large stimuli, but not for optimal or small stimuli, if I understand it correctly. Does the model allow the authors to go one step further and explain how the vLGN influences the behavioral success rate?*

We apologize for any confusion caused. In fact, there was also an effect on the maximum response of individual neurons. In **Fig. 6g,h**, the dashed curves showed the tuning curves of the actual response levels after activation/suppression and the colored solid curves showed the tuning curves after normalization (so that the shape of the tuning curve is better compared to the LED-Off condition).

The model only predicts changes in visual processing functions of individual neurons. However, from individual neurons to behavioral performance is a great leap and many factors can affect the population coding that eventually determines the behavioral success rate. It will require a lot more efforts to understand these factors. In the Discussion, we only briefly presented our idea of how the changes in visual processing of individual neurons might contribute to the improved success rate (page 21).

"At the neuronal response level, activation of the vLGN input shifts the SC size preference towards a smaller size. This may allow recruitments of more SC neurons by a smaller stimulus size, even though the evoked firing rate of individual neurons might be reduced. In addition, the surround suppression can enhance the contrast of stimulus edges [56], thus facilitating detection. Such effects together may contribute to the enhanced sensitivity to small moving objects of particular sizes when activating vLGN, resulting in improved behavioral performance."

*Next to these larger questions, I have sets of very small comments.*

*L50: "elucidate mystery of the vLGN" is missing "the"*

Thanks for pointing out this mistake. We have corrected it in the manuscript.

*L93: Was the second trial, shown in Fig 1b, measured in a second session, on another day, or in the same session as the first trial? I assume it was done in a second session, based on the methods, but perhaps this can be added explicitly to the results text or figure caption.*

It was the second session tested at least 5 min after the completion of the first on the same day. We have clarified this issue in the figure legend (page 29, line 667).

*L133: Do the authors have data on how quickly the mice adapted?*

We have added new data to show the adaptation/habituation (**Extended Data Fig. 1e**). Four trials were tested within a day, with 2-3 min inter-trial intervals. The success rate was reduced over trials ($1^{st}$ trial, 88.2%; the $2^{nd}$ trial, 76.5%, the $3^{rd}$ trial, 58.8%, the $4^{th}$ trial, 52.9%. N = 17 mice).

*L118: "SC-projecting vLGN neurons were most densely distributed in the lateral half of the nucleus" The viral injection in the image was done in the medial SC. Would it be more accurate to say use mSC-projecting vLGN neurons, or was this independent of the SC injection location?*

This is a good point. We have performed more experiments and examined images for a more lateral SC injection. Indeed, the distribution of vLGN neurons appears somewhat different from medial injection (new **Fig.1i**). So, we have removed the statement that "…most densely distributed in the lateral half of the nucleus".

*L353: "Extended Fig. 3" should be "Extended Fig. 4".*

The figure sequence has been changed, now it is "Extended Fig. 6".

*L352-354 "Also, we found that vLGN neurons were robustly activated by moving dots and tuned to large dot sizes (Extended Fig. 3). These observations indicate that vLGN can encode diverse visual information far beyond illuminance levels" But the moving dots would also change the illuminance levels, so an area purely encoding illuminance levels could respond similarly. Perhaps rephrase or include responses to gratings in the argument.*

We have now clarified that in the experiments to measure the tuning for dot size, the overall luminance was designed to be same across different dot sizes (by adjusting dot density). Besides, static dots were presented for 200 ms prior to their movements for measuring the baseline activity. Thus, the visually evoked response was to the movement of dots during which the luminance level was relatively constant (page 38, line 934).

*L828. For reproducing the results, it would be nice to mention the luminance level.*

Thanks for this helpful advice. We have added the luminance level into the Methods (page 37, line 917). The luminance level was 22~27 cd/$m^2$ in our experiments.

*L847-853: Upon first reading, it was not clear to me that this was about the water-reward training paradigm used for Figure 2 and not the paradigm used for Figure 1, which follows later in the description of the behavioral paradigms. This was a bit confusing. Perhaps it can be clarified or re-arranged.*

Thanks for pointing out this issue. We have clarified that the procedures were for trained approaching (page 41, line 1011). In addition, we have directly labeled Fig.1a as "innate approaching" and Fig.2a as "trained approaching" for clarity.

*L847-853: How long did the training and test sessions take? How many stimuli were shown? Did it mouse have single training and test session?*

For each training day, training session continued until the number of correct trials reached 50. Since the success rate was low at the beginning, and it usually took around 30~60 min. Over days, training would take less trials and shorter time. For a relatively well-trained animal, one training session was usually completed within 60 trials (i.e. 60 stimuli), which only took ~15 min (page 41, line 1022). Each mouse had multiple training and test sessions.

*L875: "Olmpus" should be Olympus.*

Corrected.

*L901: What was the extent over which the stimulus moved before reversing?*

The moving dot moved horizontally within a 7-cm range on the screen (page 40, line 995).

*L908: After the visual stimulation was terminated. When was a new trial started?*

The trial was kind of self-initiated. After the previous trial (or plus timeout), as long as the animal went back to the initiation zone and was facing the screen, a new trial was started (page 41, line 1019).

*L916: "48-hr water restriction". Was the mouse not given any access to water in this period? If so, can this be added explicitly for clarification. Was there also water restriction during the training period following this first 48-hr restriction period? If so, was water given to the animals that did not make enough successful tests, or was a different procedure followed?*

The animal was not given any access to water during the initial 48-hr water restriction. Then, during a training session ~20 µL water was given in each "Correct trial" until the correct trial number reached 50. The animal thus received about 1mL water in total each day during the training (page 41, line 1020).

*L958: The form p^y is an unusual choice of the non-linearity. Is this indeed correct or was y^p intended?*

This is correct. We found that the *p^y* function could generate size tuning curves that fit very well to our experimental data ($R^2 > 0.99$). We have also tried *y^p*, which fit to the experimental data not as well. We have added the justification to the Methods (page 43, line 1073).

*L963: "We constrained a_c, a_s >0." I do not see the variables a_c and a_s in the preceding equations. Please check.*

They are variables in equation (10). We have removed the mentioning of variables at irrelevant places.

*L1005: There is a spurious '.' in front of the b in the equation.*

Corrected.

**Response to Reviewer #3**:

*In this study, Li and colleagues investigated the influence of the ventral lateral geniculate nucleus (vLGN) on visual processing in the superior colliculus (SC) of mice. The vLGN is an understudied inhibitory prethalamic area involved associated with non-image-forming vision that has recently come into focus as a brain region that mediates visually evoked defensive behaviours (Salay and Huberman, 2021; Fratzl et al., 2021).*

*The authors of this study find that manipulating vLGN projections to the SC bidirectionally affects both instinctive and learned visually guided approach behaviour and that this effect on behaviour could be explained by the specific and contextual modulation of visuospatial response properties of SC neurons by vLGN projections.*

*This study is novel, timely and contributes to our understanding of visual pathways involving the vLGN, which have so far been less studied than the canonical thalamocortical pathway.*

*The authors have employed a wide array of state-of-the-art techniques, including behavioural manipulation experiments in freely moving mice, anatomical tracing experiments and extracellular electrophysiological recordings in different visual brain areas to study the role of vLGN in visual processing.*

*Extracellular electrophysiological recordings in both the vLGN and the SC are interesting and while they confirm previous results regarding the visual properties of vLGN neurons and the depth-dependent inhibitory effect of vLGN projections on SC neurons, they go beyond the published literature and convincingly show that vLGN can have a more nuanced and specific influence on SC computations depending on the exact visual stimuli presented.*

We thank the reviewer for all the comments.

*Specifically, vLGN input modulates the size tuning curves of SC neurons, possibly through a surround inhibition mechanism. The authors also describe a visual contextual modulation of SC responses by the vLGN, however the data shown need some clarification (see below).*

*The behaviour experiments in Figures 1 and 2 need more work. As detailed below, the authors claim that manipulating vLGN circuits changes the perception of a visual stimulus inducing a difference in a decision-making task. However, in Figure 1, it is so far unclear if execution or perception are affected. Moreover, in Figure 2, effect sizes seem partially inconsistent and it is not clear if they are not partially driven by the choice of a specific data fitting model.*

*A more thorough discussion regarding the influence of vLGN (and other prethalamic) areas on SC computations could improve the manuscript.*

*Major*

*85-115 (Figure 1): This behaviour has three possible outcomes: correct decision, wrong decision, and non-decision (trial aborted). It would be important to show all three outcomes separately, as it is known that SC (and vLGN manipulation) can affect behaviour execution. To conclude on the effect of manipulation on visual discrimination, only completed trials should be compared as it is*

*so far unclear if vLGN/SC manipulation does not just increase trial abortion rate instead of affecting visual stimulus discrimination.*

This is a good point. We have now separately profiled the three possible outcomes for silencing SC (new **Fig. 1f**) or silencing the vLGN-SC projection (new **Fig. 1g**). Both manipulations increased the number of no-decision trials, but more pronouncedly the number of incorrect trials. Thus, if only choice-made trials were considered, the success rate would still decrease. The new result further supports the notion of poorer visual discrimination after silencing the vLGN-SC projection.

*Also, the extended data Figure 1 does not fully address this issue and the conclusion on no "emotional" involvement of vLGN circuits and therefore the necessity of vLGN affecting only visual processing is not very convincing, especially as different groups, in particular Salay and Huberman, 2021 and Fratzl et al., 2021 have recently shown - in comparable settings - that vLGN manipulation has strong behavioural effects (e.g., in the plus maze and open maze). The authors mention the difference in axonal distribution of vLGN->SC projections compared to previous studies, and suggest that these could be explained by the previous works having inadvertently targeted ZI instead. There are many alternative reasons for why this could be the case. The vLGN consists of many different types of inhibitory neurons, so slight differences in injection site could explain differences in projection target. Further, different viruses used could have different tropism for different cell types. The injections shown in Fratzl et al, 2021, for example, look as localised as the ones the authors present here. If the authors wish to make this claim publicly, a thorough anatomical rendering of their injections (along the AP axis of the vLGN) would be necessary to corroborate this, as well as a comparative injection of the rostral ZI instead. The above given reasons may be similarly likely reason than ZI 'contamination', which exists also in this study. (see also comments below).*

We agree with the reviewer and have revised the text accordingly. We have acknowledged that differences in experimental parameters such as the angle and location of injection pipette may cause the variability in results among different studies. Contaminations of ZI or IGL, which may not be avoidable when using VGAT-Cre mice, may also contribute to the variability. We have added a section in Discussion to discuss this issue (page 18, line 401).

*L144-147 Figure 2: It's not clear why the authors performed an inhibition experiment of vLGN somata to show the decrease in success rate, and compare this to the increase of performance by specifically stimulating axons in the SC (which could well activate vLGN neurons through backpropagating action potentials). The interpretation of data from Figure 2F is misleading. While vLGN inhibition indeed appears to consistently reduce correct performance of the task, there does not seem to be any convincing size dependency. When ignoring the first data point of 1 deg where animals perform at chance level (50%) and which is likely below detection threshold (1 deg), it becomes apparent the vLGN inhibition mostly just downshifts performance by a more or less constant value (~10%). The fitted 50% thresholds lay in both groups between 1 and 2 and no stimuli have been probed close to these values to confirm that vLGN inhibition induces an actual change in slope besides just downshifting overall performance. Without additional datapoints close to the thresholds (1.2,1.4,1.6, 1.8 deg for example) it is unclear if the threshold change is real or just an artifact of the fitting method. P-values just below 0.05 could easily change when a single*

*point from a single animal was different at 5 deg for example. Similarly for Figure 2G it might be easier to see the raw (average trace) per mouse instead of the fit, which might be not particularly robust given that most of the change appears to happen within 2 points (1 and 2 deg). Also, surprising that the effect in 2H is much stronger than in 2F (even more apparent when comparing 2L to 2J). This points to some lack of robustness of the fitting model due to lack of data (at critical points). Overall, Figure2J is more convincing, given that the ceiling effect is avoided to the lower contrast stimulus. However, the fits on single animals (Figure 2L) still don't look convincing and the large difference in DS50% between single animals and average fit concerning. Given all those experiments were performed in a paired setting (within animal controls), it might be easier to look at the difference in success rate for each animal at each stimulus size and identify for which stimulus size the difference is the largest.*

The reviewer has raised several excellent points, and we have made changes following the reviewer's suggestion. First of all, we have performed the vLGN-SC terminal silencing experiments, using the same visual contrast level (0.3) as in the activation experiments for a better comparison. As shown in the new **Fig. 2f-g**, silencing the vLGN-SC terminals also reduced the performance, which exhibited more convincingly a size dependency.

Second, instead of curve fitting, we have now compared the difference between LED-On and LED-Off conditions across different dot sizes and found that the largest effect occurred within a range of 3°-5° dot sizes (new **Fig. 2h,m**). Therefore, the vLGN input most strongly affects the processing of specific small sizes.

*Figure 3: The analysis of visual response properties is well executed, and is in line with the results of already published work by Ciftcioglu et., 2020 and others. The authors further find that the proportion of OnOff cells is larger than previous reported. They could discuss what the reasons for this could be (again, vLGN is a vastly heterogenous brain region with many different cell types, so sth as simple as the penetration angle could in theory make a huge difference).*

We agree that experimental parameters such as the penetration angle can contribute to differences in results among different studies. We have added discussions about this issue (page 19, line 419).

*Figure 7C, F-H: The dots in none of these plots appear consistent. For example, in 7F (low SF LED on) there are 7 points at exactly 20 deg, while in 7H (low SF LED on, same condition in principle) there are 0. None of the groups seem to match their equivalent group in a different panel. Are these different neurons, was the data analysed differently or were different experiments performed for each panel? Also the average values seem significantly different (in the example mentioned around 10-12 degs in 7F vs 7.5 in 7G,H). Please clarify.*

The apparent inconsistency was due to separate single-unit sorting for different pairs of stimulus conditions in our previous analysis, although the data were from the same set of experiments. Now we have pooled all the recording data before the sorting to ensure that the same units are compared across different pairs of conditions. Data are now consistent across panels (see new **Fig. 7f-h**). Fig. 7c was from a different set of experiments.

*Minor*

*L85-115 (Figure 1): It is unclear why SC inhibition is compared to vLGN inhibition (and not vLGN excitation) given that vLGN is mostly inhibitory and vLGN activation decreases SC activity (as shown later in the paper and in Fratzl et al., 2021). Particularly surprising given that in extended data figure 1 both vLGN stimulation and inhibition experiments are shown. Does vLGN stimulation not induce a consistent behavioural effect in the approach experiment? Is a bidirectional effect expected (inhibition or excitation) or is vLGN manipulation just expected to disrupt visual discrimination.*

We had not intended to compare SC inhibition to vLGN inhibition. The purpose of SC inhibition experiments is only to demonstrate that the approaching behavior is SC-dependent.

We do not think that the SC silencing has to be comparable to vLGN excitation either, since the former experiment can lead to a complete silencing of activity in SC across layers while the latter at most moderately reduces firing rates in some specific sublayers of SC. Activation of vLGN is expected to improve visual discrimination based on our results. While we did not observe a significant change in the success rate of innate approach in our early vLGN stimulation experiment (data not shown), this could possibly due to a ceiling effect of using a high stimulus contrast in that experiment.

*L117: AAVretro-GFP? Should be AAVretro-Cre?*

Corrected.

*L123-129: The authors mention that GABAergic projections from the vLGN predominantly terminate in the most superficial SC layers, which seems at least partially different from what Salay and Huberman, 2021 and Fratzl et al., 2021 have shown regarding additional projections to the intermediate SC layers. Please discuss possible reasons, see also above.*

*The authors claim that this discrepancy is most likely due to a more prominent contamination of zona incerta (ZI) neurons in the former mentioned papers. While this remains a valid hypothesis, Fratzl et al., 2021 proposed (supplemental figures) that the most superficial projections could arise from intergeniculate nucleus (IGL) neurons (not mentioned here at all). Also, in the present Figure 1K, projections in deeper SC layers and PAG can still be seen, even if the fluorescence appears less bright than in sSC.*

The reviewer has made a good point about IGL. In our added discussion, we have acknowledged the possibility of IGL axons contributing to superficial projections (page 19, line 413). We would like to point out that although we did see vLGN projections to deeper SC layers, they were mostly in the medial part of SC. This is somewhat consistent with the image in Salay and Huberman, 2021 paper (their Figure 5D) where projections to deep SC are nearly absent in the lateral SC while those to superficial SC are still relatively strong in the lateral SC. As we have discussed now, different experimental parameters can contribute to differential anatomical as well as functional observations (page 18-19).

*Moreover, throughout the manuscript there seems to be some variability in the shown projection patterns (possibly due to normal variability in the extent of contamination in ZI and IGL), for example in Figure 4B many more axons in the deeper layers can be seen (exactly at the recording site of the probe, consistent with the pattern described by Salay and Huberman, 2021 and Fratzl et al., 2021). Similarly, in Figures 4M-O, the authors show that most SC neurons receiving direct input from vLGN are located significantly deeper than the shown axonal projections in Figure 1K. While extensive apical dendrites (line 222) are possible, the authors should also acknowledge the possibility of direct axonal vLGN projections to the intermediate SC layers, which could also explain the strongest effect of vLGN manipulation on intermediate SC circuits (Figure 4P).*

We agree with the reviewer and have acknowledged that vLGN axons also project directly to intermediate SC layers (page 16, line 343).

*This is also corroborated by Figures 8D-G, where authors show that vLGN neurons receiving V1 input (which likely represent the majority of vLGN neurons according to Govindaiah et al., 2023 and Sumitomo et al.,1979) project significantly deeper than what is shown in Figure 1K.*

We would like to point out that in Fig. 8d-g (now **Fig. 8f-g**), there was no significant contamination of IGL axons (see the image in Fig.8f) since the injection in vLGN was very local. Nevertheless, we observed the densest projections in the ventral portion of sSC. References mentioned have been cited.

*Finally, it is also possible that some axons in the sSC are labelled due to the retrograde transport of AAV1 (for example Zingg et al., 2017 Figure S1) labelling directly retinal ganglion cells which are known to project extensively in the most superficial SC layer (including VGAT+ RGCs (Cai et al., 2021). This could be ruled out by retinal histology.*

This is a good point. We reason if GABAergic retinal ganglion cells were retrogradely labeled, we would be able to see strong labeling in dLGN. However, there was not much labeling in dLGN (see **Fig. 1k**), indicating that contamination of VGAT+ RGCs was unlikely.

*Given the yet not fully cohesive picture of how vLGN and the other caudal prethalamic nuclei IGL and ZI (Fratzl and Hofer, 2022) innervate SC circuits, it could be considered adding a specific section in the discussion of this manuscript.*

Following the reviewer's suggestion, we have added a specific section in the Discussion (page 18-19). We acknowledge that how vLGN and other caudal prethalamic nuclei innervate SC circuits is still not fully understood and that future studies by using more refined cell-type-specific Cre lines or projection-based approaches are needed.

*L139: VGT+ ->VGAT+*

Corrected.

*L206-210 (Figure 4): Worth mentioning that the result that the effect of vLGN stimulation on SC visual responses being strongest in intermediate layers is not novel but as been shown by Fratzl et al., 2021 (Figure 4), where they performed a nearly identical experiment.*

The result of Fratzl et al. study has been referenced here (page 11, line 225).

*L216-217: It is not novel that SC neurons in deeper layers have larger receptive fields. It has been shown several times before (for example Lee et al., 2020 Figure 3).*

The result of Lee et al. study has been referenced here (page 11, line 233).

*L228 (Figure 4 and 5): The authors mention that they mostly record in the lateral SC, however, the histology shown Figure 4B is quite medial, at most central SC, but not lateral.*

The results in Fig. 4 contained both the lateral and medial SC recordings and this has been clarified in the text (page 10, line 215). Representative images of electrode tracks for both medial and lateral recordings are now shown in **Extended Date Fig. 4b**. For Fig.5, the recording was from the lateral part of SC. We have now modified the illustrations in Fig. 5 (more lateral position of the electrode) to avoid confusion.

*Figure 5: Results presented in Figure 5 are very interesting and convincing. The size dependent effect of vLGN manipulation might even be more convincing if in additional panels the spike difference between laser and no laser could be shown (for example in supplemental) dependent on the size of the stimulus (more or less the difference between the traces in Figure 5B, 5D etc.).*

Following the reviewer's suggestion, we have now compared the PSTH traces between LED-On and LED-Off conditions across different stimulus sizes (new **Extended Data Fig. 5**).

*Figure 6A: The schematic for the SSI is useful, however, it would be good to add to which actual SSI value this corresponds (presumably around 0.5?)*

The corresponding SSI value (0.4) has been added in the **Fig. 6a**.

*Figures 6C-6E: Not clear if the fold change is more meaningful than a simple SSI difference, given that small baseline values will increase to uninterpretable large values.*

We have switched to plot ΔSSI in **Fig. 6c**, **6e**.

*Figure 7: Maybe helpful to add a plot similar to 7B for the LED ON conditions.*

We have added a plot for the LED On condition in the **Extended Data Fig. 9c**.

*Figures 8H-L: Please add to panel what experiment was recorded in cortex vs which ones in vLGN.*

We have added labels in now **Fig. 8j-k** to indicate the site of recording.

*L307-310: Unclear how neurons labelled in SC after retroAAV injection in vLGN confirm the vLGN anterograde tracing results. This is the opposite experiment, and regardless it is known that SC projects back to vLGN.*

The AAVretro injection in vLGN only labeled very superficial SC neurons (Fig.8b), indicating that SO and InG neurons do not project to vLGN. The latter neurons were labeled by injecting AA1-Cre in vLGN (Fig.4o), thus indicating bona fide anterograde labeling.

*L313-315: See comment regarding vLGN projections to intermediate SC layers.*

We have revised the text and acknowledged the fact that vLGN axons project to intermediate layers of SC (page 16, line 343).

*L318-322: Please comment on how this result is consistent with Govindaiah et al., 2023, who found driver-like input from cortex to vLGN.*

We have commented on the result of Govindaiah et al., 2023 study (page 16, line 349). Using slice whole-cell recording, they demonstrated that retinal and cortical connections to a subpopulation of vLGN neurons are potentially comparable in strength. Here, we found that in *in vivo* conditions visually evoked responses of vLGN neurons are mainly driven by retinal input.

*L414-419: This model has been proposed before, for example by Fratzl and Hofer, 2022 (their Figure 6B-C is very similar to the here presented Figure 8M). The authors should at least cite to acknowledge.*

We have cited Fratzl and Hofer 2022 study here (page 22, line 482).

REVIEWERS' COMMENTS

Reviewer #1 (Remarks to the Author):

General comments:

Most of the comments have been addressed and additional experiments have been added, strengthening some of the findings and narrowing potential interpretations. In addition, the discussion has been greatly improved and includes new points thus providing context to the current findings. However, the manuscript would greatly benefit from additional justifications, further proofreading and consistency as mentioned below:

Specific comments for figures:
Figure 1:
Figure 1a: Incorret
Figure 1C: same animals/trials as b? Maybe label "Successful trials (%)" instead of percentage?
Figure 1D and E: Animal group size are inconsistent (57 vs 29)

Figure 2:
Figure 2F and K: displaying individual data points or using a violin plot would help visualize the performance distribution.
Figure 2I and N: It is unclear what the data points correspond to. If it was average across animals, there should be 6 and 9, it was sessions (which should have roughly the same occurrence of conditions), it should be 36 and 54. So what are we looking at?

Figure 3: Number of animals only mentioned up to e. Why were not all the neurons in e, 320, included in f, n=167? Group size are highly variable, for example panel 3n: n = 167, 60, 117 and 537 neurons. How many mice per group then?

Figure 4:
Except for 4j, all fold changes are between -1 and +1, could the method briefly (one sentence) describe how the fold change was calculated? The following is assumed:
Normalized FR= Peak_response-Peak_spontaneous
Fold changes = (NormFR_LEDOFF - NormFR_LEDON) / NormFR_LEDOFF
Are all firing rates peak or average? (only mentioned for h, line 743)
Figure 4m: Ai75 Floxed TdTomato?

Figure 5: What is the proportion of cells that are unaffected?

Figure 7: Effects are less conclusive with the silencing being restricted to SC projecting vLGN axon manipulation (line 325).

Figure 8N: Is there evidence that these inputs converge onto the same cell? Especially the retinal inputs? It might be more appropriate to have these connections target the SC rather than a single point which seems to represent a single neuron.

Specific comments for main text:
Line 129: densely?
Line 137-139: They results show that they are anatomically connected, nothing can be inferred about visually evoked activity from anatomical tracings
Line 235: Ai75?
Line 439: 8m?
Line 475: 8m?
Line 868: pAAV?

Reviewer #2 (Remarks to the Author):

The authors addressed all my comments and answered all my questions to my full satisfaction. I think the paper gives a novel and important contribution to our knowledge of the neural circuitry of visuomotor behaviors.

I spotted one remaining typo. In Fig 1a, “Incorret” should be “Incorrect”.

Reviewer #3 (Remarks to the Author):

Dear authors, dear editors,

The authors have answered all open questions with new experiments, re-analysis and manuscript changes, I have no further comments. I congratulate the authors on a beautiful study and fully support its publication.

**Response to Reviewer #1:**

*General comments:*

*Most of the comments have been addressed and additional experiments have been added, strengthening some of the findings and narrowing potential interpretations. In addition, the discussion has been greatly improved and includes new points thus providing context to the current findings. However, the manuscript would greatly benefit from additional justifications, further proofreading and consistency as mentioned below:*

*Specific comments for figures:*

*Figure 1:*

*Figure 1a: Incorret*

Done. Corrected to "Incorrect".

*Figure 1C: same animals/trials as b? Maybe label "Successful trials (%)" instead of percentage?*

Yes, these are the same animals/trials as in **b**. We have changed the y-axis label to "Successful trials (%)" following the reviewer's suggestion.

*Figure 1D and E: Animal group size are inconsistent (57 vs 29)*

We have explained the group sizes in Methods (page 26). As we have shown in Extended Data Fig. 1e, the animals would quickly become adapted to the dot stimulation in innate approaching tests. To avoid the confounding adaptation effect on behavioral performance, we used two groups of mice in dot contrast and dot size tests. Since we wanted to carefully measure the dots size tuning in the following experiments, we included a larger number of mice in the test of dot diameters.

*Figure 2:*

*Figure 2F and K: displaying individual data points or using a violin plot would help visualize the performance distribution.*

Individual data points have now been plotted in F**ig. 2f**, **2k** to show distribution.

*Figure 2I and N: It is unclear what the data points correspond to. If it was average across animals, there should be 6 and 9, it was sessions (which should have roughly the same occurrence of conditions), it should be 36 and 54. So what are we looking at?*

Previously, we grouped the success rates for each dot size range (therefore, two-three data points from each animal). Since this may cause confusion, we have plotted the average success rate within each dot size range in revised **Fig. 2i** and **2n**. Now, the number of data points is consistent with the number of animals.

*Figure 3: Number of animals only mentioned up to e. Why were not all the neurons in e, 320, included in f, n=167? Group size are highly variable, for example panel 3n: n = 167, 60, 117 and 537 neurons. How many mice per group then?*

We have specified animal numbers for all recording experiments in the figure legend (vLGN, n = 7 mice; dLGN, n = 3 mice; V1, n = 2 mice; SC, n = 10 mice). For **Fig. 3f**, we only included neurons showing excitatory responses to both On and Off stimulation (167 out of 320) to calculate the ocular bias index. The ocular bias of other neurons is explicit from illustration in **Fig. 3e**.

*Figure 4:*

*Except for 4j, all fold changes are between -1 and +1, could the method briefly (one sentence) describe how the fold change was calculated? The following is assumed:*

*Normalized FR= Peak_response-Peak_spontaneous*

*Fold changes = (NormFR_LEDOFF - NormFR_LEDON) / NormFR_LEDOFF*

*Are all firing rates peak or average? (only mentioned for h, line 743)*

We have clarified how visual responses are quantified in Methods (page 32):

$$\text{Norm. FR} = (\text{Average\_response} - \text{Average\_spontaneous}) / \text{Average\_spontaneous}$$

$$\text{Fold Change} = (\text{Norm.FR\_LED}_{\text{OFF}} - \text{Norm.FR\_LED}_{\text{ON}}) / \text{Norm.FR\_LED}_{\text{OFF}}$$

*Figure 4m: Ai75 Floxed TdTomato?*

We have changed the labeling to "Floxed tdTomato" in **Fig. 4m**.

*Figure 5: What is the proportion of cells that are unaffected?*

All recorded cells were included in Fig.5. Readers can read from **Fig. 5c**, **e**, **h**, **j** to appreciate how many cells were unaffected.

*Figure 7: Effects are less conclusive with the silencing being restricted to SC projecting vLGN axon manipulation (line 325).*

The effect of silencing the terminals may appear smaller than silencing the cell bodies but is still statistically significant (with two stars, **Fig. 7i**). We have changed the wording (line 323).

*Figure 8N: Is there evidence that these inputs converge onto the same cell? Especially the retinal inputs? It might be more appropriate to have these connections target the SC rather than a single point which seems to represent a single neuron.*

This is a good point. We have illustrated many SC cells in revised **Fig. 8n** to avoid the impression that different projections converge onto the same cell.

*Specific comments for main text:*

*Line 129: densely?*

We have removed "densely".

*Line 137-139: They results show that they are anatomically connected, nothing can be inferred about visually evoked activity from anatomical tracings.*

We have revised the sentence about visually evoked activity (line 136).

*Line 235: Ai75?*

Yes, it is "Ai75".

*Line 439: 8m?*

Corrected to "**Fig. 8n**" (line 434).

*Line 475: 8m?*

Corrected to "**Fig. 8n**" (line 465).

*Line 868: pAAV?*

Corrected to "AAV2" (line 499).

**Response to Reviewer #2:**

*The authors addressed all my comments and answered all my questions to my full satisfaction. I think the paper gives a novel and important contribution to our knowledge of the neural circuitry of visuomotor behaviors.*

*I spotted one remaining typo. In Fig 1a, "Incorret" should be "Incorrect".*

We have corrected it to "Incorrect".

Reviewer #3 has no further comments.
